# Simplification to Abacavir/Lamivudine + Atazanavir Maintains Viral Suppression and Improves Bone and Renal Biomarkers in ASSURE, a Randomized, Open Label, Non-Inferiority Trial

**DOI:** 10.1371/journal.pone.0096187

**Published:** 2014-05-13

**Authors:** David A. Wohl, Laveeza Bhatti, Catherine B. Small, Howard Edelstein, Henry H. Zhao, David A. Margolis, Edwin DeJesus, Winkler G. Weinberg, Lisa L. Ross, Mark S. Shaefer

**Affiliations:** 1 AIDS Clinical Trials Unit, University of North Carolina at Chapel Hill, Chapel Hill, North Carolina, United States of America; 2 AIDS Healthcare Foundation, Beverly Hills, California, United States of America; 3 New York Medical College, Valhalla, New York, United States of America; 4 Alameda County Medical Center, Oakland, California, United States of America; 5 GlaxoSmithKline, Research Triangle Park, North Carolina, United States of America; 6 Orlando Immunology Center, Orlando, Florida, United States of America; 7 Kaiser Foundation Health Plan of Georgia, Inc, Atlanta, Georgia, United States of America; 8 ViiV Healthcare, Research Triangle Park, North Carolina, United States of America; University of Ottawa, Canada

## Abstract

**Objective:**

Simplification of antiretroviral therapy in patients with suppressed viremia may minimize long-term adverse effects. The study’s primary objective was to determine whether abacavir/lamivudine + atazanavir (ABC/3TC+ATV) was virologically non-inferior to tenofovir/emtricitabine + atazanavir/ritonavir (TDF/FTC+ATV/r) over 24 weeks in a population of virologically suppressed, HIV-1 infected patients.

**Design:**

This open-label, multicenter, non-inferiority study enrolled antiretroviral experienced, HIV-infected adults currently receiving a regimen of TDF/FTC+ATV/r for ≥6 months with no history of virologic failure and whose HIV-1 RNA had been ≤75 copies/mL on 2 consecutive measurements including screening. Patients were randomized 1∶2 to continue current treatment or simplify to ABC/3TC+ATV.

**Methods:**

The primary endpoint was the proportion of patients with HIV-RNA<50 copies/mL at Week 24 by the Time to Loss of Virologic Response (TLOVR) algorithm. Secondary endpoints included alternative measures of efficacy, adverse events (AEs), and fasting lipids. Exploratory endpoints included inflammatory, coagulation, bone, and renal biomarkers.

**Results:**

After 24 weeks, ABC/3TC+ATV (n = 199) was non-inferior to TDF/FTC+ATV/r (n = 97) by both the primary analysis (87% in both groups) and all secondary efficacy analyses. Rates of grade 2–4 AEs were similar between the two groups (40% vs 37%, respectively), but an excess of hyperbilirubinemia made the rate of grade 3–4 laboratory abnormalities higher in the TDF/FTC+ATV/r group (30%) compared with the ABC/3TC+ATV group (13%). Lipid levels were stable except for HDL cholesterol, which increased significantly in the ABC/3TC+ATV group. Bone and renal biomarkers improved significantly between baseline and Week 24 in patients taking ABC/3TC+ATV, and the difference between groups was significant at Week 24. No significant changes occurred in any inflammatory or coagulation biomarker within or between treatment groups.

**Conclusions:**

After 24 weeks, simplification to ABC/3TC+ATV from TDF/FTC+ATV/r maintained viral suppression was well-tolerated, and led to improvements in bone and renal biomarkers and HDL cholesterol.

**Trial Registration:**

ClinicalTrials.gov NCT01102972

GlaxoSmithKline Clinical Study Register #113734

## Introduction

Atazanavir (ATV) is a well-tolerated protease inhibitor (PI) whose plasma concentration and genetic barrier to resistance are improved by coadministration with low-dose ritonavir (RTV) [1], [2]. However, use of RTV is associated with higher rates of gastrointestinal adverse events, elevated lipid levels and, when given with ATV, an increased risk of indirect hyperbilirubinemia [1], [3]–[5]. As an inhibitor of the CYP3A metabolic pathway, RTV also interacts with many drugs and co-administration is contraindicated for a number of antiarrhythmics, ergot derivatives, sedative/hypnotics, and statins, among others. [4].

Numerous induction-maintenance strategies in which RTV is discontinued once viral suppression has been achieved have been investigated and have been found to be effective [5]–[13]. Treatment guidelines from the US Department of Health and Human Services (DHHS) endorse these types of regimen simplifications, citing the benefits of reduced pill burden, enhanced tolerability, improved quality of life, and decreased risk of some long-term toxicities [14].

The primary objective of the study was to determine whether abacavir/lamivudine + atazanavir (ABC/3TC+ATV) was virologically non-inferior to tenofovir/emtricitabine + atazanavir/ritonavir (TDF/FTC+ATV/r) over 24 weeks in a population of virologically suppressed HIV-1 infected patients with no history of prior virologic failure whose virus was currently suppressed on a regimen of TDF/FTC plus ATV/r) one of four preferred first-line regimens in the DHHS guidelines [14]. An induction-maintenance strategy is particularly well-suited to ATV, which is licensed in the United States for use either unboosted (daily dose of ATV 400 mg) or boosted (daily dose of ATV 300 mg and RTV 100 mg) [5]. However, since TDF/FTC is not recommended for use with unboosted ATV because of a drug-drug interaction that results in decreased concentrations of ATV [5], patients in this trial who were randomized to discontinue RTV simultaneously switched to a nucleoside reverse transcriptase inhibitor (NRTI) backbone of abacavir sulfate/lamivudine (ABC/3TC).

This paper presents the primary endpoint of the study (percentage of patients maintaining viral suppression at Week 24) and associated secondary analyses including immunologic response, virologic efficacy, HIV-1 genotypic and phenotypic resistance patterns in patients who experienced confirmed virologic failure as well as the tolerability and safety of these regimens, including changes from baseline in fasting lipids and biomarkers associated with cardiovascular, bone, and renal health.

## Methods

The ASSURE study (EPZ113734; NCT01102972) was a prospective, randomized, multicenter, open-label, phase IV study in HIV-infected, HLA-B*5701-negative patients ≥18 years of age who were virologically suppressed (defined as HIV-1 RNA ≤75 copies/mL on 2 consecutive occasions; screening and ≥28 days prior) on a once-daily regimen of TDF/FTC (300 mg/200 mg) and ATV/r (300 mg/100 mg) for ≥6 months immediately prior to screening.

The study design, outcome measures and results summary are also available on the ClinicalTrials.gov site (http://clinicaltrials.gov; registration number is NCT01102972) as well as on the GlaxoSmithKline Clinical Trials registry (http://www.gsk-clinicalstudyregister.com; GSK ID number 113734). The CONSORT Checklist has been included as supplementary material (**Checklist S1**).

The full study protocol has been included as supplementary material (**Protocol S1**), and contains the full listing of all inclusion and exclusion criteria. The main inclusion criteria included a requirement that enrolled patients were ART-experienced, HIV-1 infected and ≥18 years of age and receiving a once-daily regimen of TDF/FTC (300 mg/200 mg)+ATV/r (300 mg/100 mg) for at least 6 months prior to the first day of Screening. TDF/FTC+ ATV/r must have been the subject’s initial regimen or first or second switch regimen. Initial regimen was defined as the first regimen received by a previously antiretroviral-naïve patient and any change of ART whether of a single drug or multiple drugs simultaneously was considered a regimen switch. Patients must not have switched due to virologic failure. If TDF/FTC+ ATV/r was the patient’s first or second switch regimen, then the subject should have received any of the following prior regimens: any currently licensed non-nucleotide reverse transcriptase inhibitor (NNRTI) in combination with either TDF/FTC or zidovudine (ZDV)/3TC; a ritonavir-boosted PI in combination with TDF/FTC or ZDV/3TC. Alternative prior regimens not listed above were allowed on a case-by-case basis. Each patient must have been virologically suppressed (defined as HIV-1 RNA≤75 copies/mL) at two consecutive time points on TDF/FTC+ATV/r which had to include the Screening visit and one additional visit at least 28 days prior to Screening. The original protocol was amended on 07 September 2010 to define virologic suppression as HIV-1 RNA≤75 copies/mL; for patients enrolled prior to that date, the original protocol required suppression of HIV-1 RNA to ≤50 copies/m.

The main patient exclusion criteria included having active CDC Clinical Category C disease, ongoing clinically relevant hepatitis and/or chronic hepatitis B infection (HBsAg+), being HLA-B*5701 positive, prior abacavir exposure, creatinine clearance <50 ml/min via the Cockroft-Gault method, and known hypersensitivity to any component of the study drugs. Patients were also deemed ineligible if enrolled in one or more investigational drug protocols within 30 days of screening, if they had been immunized within 30 days prior to first dose of any study drugs or had been treated with or had an anticipated need during the study for any medications which could interact with the study medications according to the product labeling. Exclusion criteria also included treatment with immune-modulating agents within the prior 90 days, immunization with an HIV-1 immunotherapeutic vaccine, or undergoing treatment with radiation therapy or cytotoxic chemotherapeutic agents (or having an expected need for the latter during the study). Any verified Grade 4 laboratory abnormality at screening was generally exclusionary unless the investigator provided a compelling explanation, as was any other laboratory abnormality or medical condition detected at screening that the investigator determined would have precluded the patient’s participation in the study. While a baseline genotype was not required for study entry, patients were deemed ineligible if they had known HIV genotyping results that indicated the presence of any mutation in the reverse transcriptase or protease regions that was associated with resistance to any study drug and included the following reverse transcriptase mutations: K65R, K70E, L74V, M184I/V, or Y115F, a combination of two or more thymidine analog mutations: M41L, D67N, K70R, K219Q or E that must include a change at either L210 or T215, or three or more of the following HIV-1 protease mutations associated with atazanavir resistance: D30, V32, M36, M46, I47, G48, I50, I54, A71, G73, V77, V82, I84, N88, and L90. There were no restrictions on screening CD4+ cell count. Females of child-bearing potential were eligible if they had negative pregnancy tests at screening and baseline and agreed to use ≥1 protocol-prescribed method of contraception throughout the study.

### Ethics Statement

The protocol (**Protocol S1**) was approved by the institutional review board (IRB) used by the 44 participating study sites. These IRBs included: Chesapeake Research Review, Inc., University (U) of Tennessee IRB, Alameda County Medical Centre IRB; Michigan State U Biomedical and Health IRB; Minneapolis Human Subjects Research Committee; Georgetown U IRB; Western IRB; St. Michaels Medical Center IRB, New York Medical College IRB, Kaiser Permanente Center for Health Research IRB and the Office of Human Research Ethics U of North Carolina IRB. All patients provided written informed consent to participate in the study.

### Design and Intervention

After stratification by prior ART experience (TDF/FTC+ATV/r as initial regimen or as first/second switch regimen), eligible patients were randomized 2∶1 to simplify to their regimen to once-daily fixed dose combination of ABC/3TC 600 mg/300 mg; (ViiV Healthcare, Research Triangle Park, NC) plus ATV 600 mg (Bristol-Myers Squibb, Princeton, NJ) or remain on the fixed dose combination of TDF/3TC (Gilead Sciences, Foster City, CA) plus ATV boosted with RTV (AbbVie, Chicago, IL). Randomization (by parallel group, stratified, and centralized with a fixed block size of 3) and study drug provisioning were performed by GlaxoSmithKline’s Randomization and Medication Ordering System (RAMOS), which uses interactive voice-response technology.

### Procedures and Assessments

Patients were evaluated at screening, baseline, and weeks 2, 4, 12, and 24 with routine chemistry, hematology, and immunology tests conducted at each assessment. The concentrations of specific biomarkers (bone alkaline phosphatase, parathyroid hormone, C-terminal telopeptide, osteocalcin, urine β2 microglobulin/creatinine ratio, high-sensitivity C reactive protein [hs-CRP], interleukin-6, and D-dimer) were evaluated at baseline and Week 24 only. Biomarker data was log-transformed prior to analysis and the change from baseline was assessed using geometric mean ratios with 95% confidence intervals. HIV-RNA concentrations were measured by the Abbott RealTime HIV-1 Assay. Adverse events (AEs) and laboratory toxicities were graded using the 2004 Division of AIDS Toxicity Grading Scale. All suspected ABC hypersensitivity events were reported as serious adverse events (SAEs). Glomerular filtration rates (GFR) were estimated using the modification of diet in renal disease (MDRD) formula [15]. Smoking status and presence of diabetes were assessed at baseline and Week 24 for Framingham Risk Score calculation [16] and treatment emergent changes in fasting total cholesterol, HDL, LDL, and triglycerides were assessed using the Division of AIDS lipid toxicity grading system [17].

Viral genotypes and phenotypes were performed at Monogram Biosciences Inc. (South San Francisco, California, USA). Genotypic mutations were defined according to International AIDS Society-USA Guidelines [2].

All other laboratory tests were performed centrally by Quest Diagnostics (Van Nuys, California, USA). Laboratory staff at Monogram and Quest performing assessments were blinded as to patient treatment assignment and sample labels included no information on assigned treatment regimen.

### Study Populations

The Intent-To-Treat (ITT) population consisted of all enrolled subjects randomized in the study, regardless of what treatment was actually received or the eventual outcome of study participation. A modified ITT population, ITT-Exposed, including all subjects who are in the ITT population and received at least one dose of study drug, was the primary population for efficacy analyses. The safety population consisted of all randomized subjects with the exception of those with documented evidence of not having consumed any investigational product. The virologic failure population for the virology analysis included all subjects who met the protocol virologic failure criterion which was defined as plasma HIV-RNA ≥400 copies/mL on 2 consecutive occasions (confirmed virologic failure). The Observed dataset contains all data collected while subjects were in the study. No missing values were imputed.

### Outcome Measures

The primary efficacy endpoint of this study was the proportion of patients with plasma HIV-1 RNA<50 copies/mL at Week 24 by the Time to Loss of Virologic Response (TLOVR) algorithm stratified by prior ART experience (TDF/FTC+ATV/r as initial regimen or as first/second switch regimen). The analysis was based on the intent-to-treat (ITT) exposed population, which included all patients exposed to at least 1 dose of any study drug. A study responder at week 24 was defined as a patient who had achieved confirmed (2 consecutive) plasma HIV-1 RNA<50 copies/mL and remained suppressed (no confirmed viral rebound measurements) by week 24 as defined by TLOVR algorithm. All others, including those who did not complete week 24 assessments, were defined as study non-responders.

Secondary endpoints measured at Week 24 included the percentage of patients with HIV-RNA<50 copies/mL as measured by the missing/discontinuation equals failure (MD = F) analysis and in the Observed dataset, the percentage of patients with HIV-RNA<400 copies/mL as measured by TLOVR, MD = F and Observed analyses, the change from baseline in CD4+ cell count, time to virologic failure, detection of genotypic and phenotypic resistance at the time of virologic failure, change from baseline in fasting lipid profiles (total cholesterol, LDL, HDL, triglycerides), grade 2–4 AEs, and all SAEs. There were two exploratory endpoints: (1) change from baseline in neurocognition scores, which will be discussed in a separate manuscript; and (2) change from baseline in eight cardiovascular/inflammation, bone, and renal biomarkers.

### Statistical Analyses

A total of 300 patients were planned for the study. Three hundred patients with a 2∶1 ratio would provide over 90% power to assess the non-inferiority of ABC/3TC+ATV over TDF/FTC-ATV/r at the 0.05 level of significance. This sample size calculation assumed a virologic response rate (proportions of patients with HIV-1 RNA<50 copies/mL) of 89% in both treatment groups and a non-inferiority margin of 12%. Non-inferiority was defined as a 2-sided 95% confidence interval (CI) that excluded differences as large as 12% in the direction of inferiority of ABC/3TC+ATV group. The non-inferiority margin of 12% is standard in such non-inferiority trials of ART and is accepted by the FDA. To establish non-inferiority, the 2-sided 95% CI should lie entirely to the right of the value of −12%.

All the statistical comparisons were based on 2-sided significance level of 0.05. Virologic response rates were compared using a Cochran-Mantel-Haenszel test stratified by initial ART regimen. Continuous variables were assessed by Wilcoxon rank-sum test, whereas binary responses were compared by Fisher’s exact test. The statistical analyses were performed using SAS Version 9.1 (SAS Institute Inc., Cary, NC) on a UNIX platform.

## Results

### Patient Characteristics and Accountability

**Figure 1:**
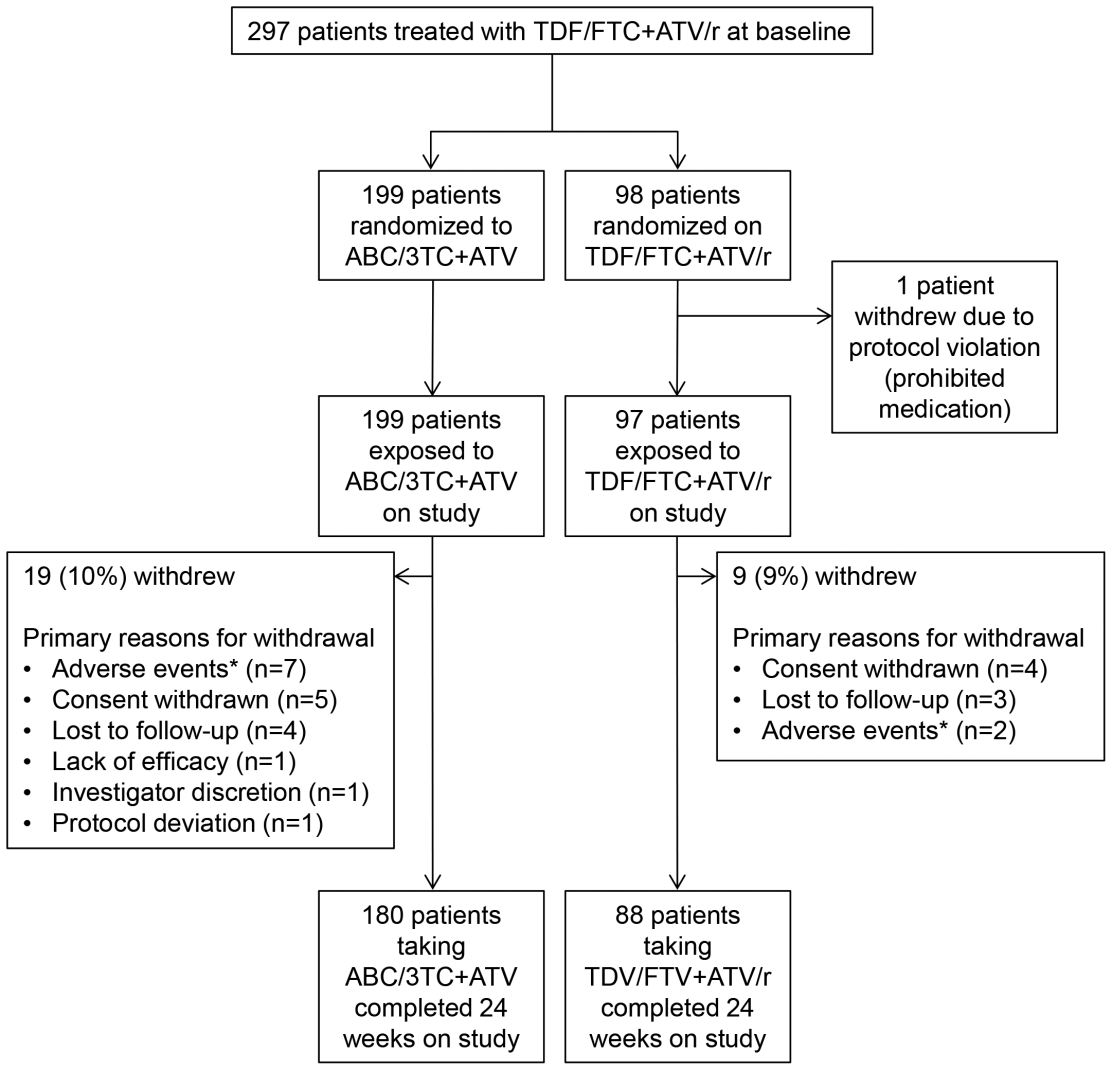
Patient disposition. Primary reasons for withdrawal were listed by the investigator on the case report form. *All adverse events leading to study discontinuation were grade 1 or 2, with the exception of a grade 3 lipase increase in one patient continuing on TDF/FTC+ATV/r.

This study enrolled 297 patients from clinical sites the United States and Puerto Rico; 1 patient randomized to the TDF/FTC+ATV/r was withdrawn due to a protocol violation prior to receiving any study medication such that the exposed population included 199 simplifying to ABC/3TC+ATV and 97 continuing TDF/FTC+ATV/r (Figure 1). Patients were recruited between April 2010 through December 2011, and the last 24-week analysis visit occurred in June, 2012. Most (79%) were male (Table 1), with 34% of patients being of African-American/African heritage and 26% declaring themselves to be Hispanic/Latino. The median baseline CD4+ cell count was 491.5 cells/µL. Median time on ART prior to enrollment was 978 days for the ABC/3TC+ATV group and 1106 days for the TDF/FTC+ATV/r group.

**Table 1:** Baseline patient demographics and characteristics.

	ABC/3TC+ATV (N = 199)	TDF/FTC+ATV/r (N = 97)
Median age, years (range)	44 (21–66)	42 (20–68)
Male, n (%)	155 (78)	79 (81)
Race, n (%)		
African American/African Heritage	65 (33)	37 (38)
White/Caucasian/European Heritage	122 (61)	55 (57)
Other	12 (6)	5 (5)
Hispanic or Latino Ethnicity, n (%)	51 (26)	26 (27)
Median plasma HIV-1 RNA, log_10_ copies/mL	1.59	1.59
<50 copies/mL, n (%)	192 (96)	93 (96)
50 to 74 copies/mL, n (%)	2 (1)	2 (2)
≥75 copies/mL, n (%)	5 (3)	2 (2)
Median CD4+ cell count, cells/µL (range)	492 (77–1196)	480 (108–1479)
<200 cells/µL, n (%)	14 (7)	6 (6)
CDC classification for HIV infection, n (%)		
Category A	136 (68)	67 (69)
Category B	26 (13)	13 (13)
Category C	37 (19)	17 (18)
Hepatitis C coinfection, n (%)	18 (9)	8 (8)
Median time on prior ART, days (range)	978 (177–4830)	1106 (199–7078)

ART = antiretroviral therapy.

Ninety-one percent (268/296) of randomized patients in the overall ITT-E population completed 24 weeks on study (Figure 1). Two patients in the ABC/3TC+ATV treatment group and 3 patients in the TDF/FTC+ATV/r treatment group who completed 24 weeks on study were allowed to switch to an alternate regimen during this period for appropriate patient management as specified in the protocol (**Protocol S1**). The most common reasons for study withdrawal were consent withdrawn (3% [9/296]), adverse events (3% [9/296]), and lost to follow-up (2% [7/296]).

### Non-inferiority Analysis

The primary study endpoint was the proportion of patients with HIV-1 RNA<50 copies/mL at Week 24 by the TLOVR analysis. Following randomization, 86.9% (173/199) of patients in the ABC/3TC+ATV treatment group successfully maintained HIV-1 RNA below 50 copies/mL at Week 24 compared to 86.6% (84/97) of patients taking TDF/FTC+ATV/r. The adjusted treatment difference was 0.33%, and the two sided 95% CI (−7.97% to 8.64%) stratified by prior ART excluded the predefined non-inferiority margin of −12%, demonstrating the non-inferiority of the ABC/3TC+ATV simplification regimen to the TDF/FTC+ATV/r continuation regimen.

### Secondary Virologic and Immunologic Response Analyses

Secondary analyses of HIV-1 RNA<50 copies/mL were similarly robust, with an Observed analysis response rate of 95% (171/181) vs. 98% (87/89) and an MD = F analysis response rate of 85% (169/199) vs. 88% (85/97), respectively, for the ABC/3TC+ATV and TDF/FTC+ATV/r groups. The TLOVR analysis of HIV-1 RNA<400 copies/mL also supported non-inferiority, with response rates of 88.4% (176/199) in the ABC/3TC+ATV group vs. 86.6% (84/97) for the TDF/FTC+ATV/r group, with an adjusted TLOVR treatment difference of 1.84% (95% CI: −6.33,10.02).

The mean increase in CD4+ cell count from baseline to Week 24 was significantly larger (*P = *0.013) for the ABC/3TC+ATV group (+48 cells/µL) compared with the TDF/FTC+ATV/r group (+8 cells/µL). At Week 24, mean CD4+ cell counts were 569 and 562 cells/µL for ABC/3TC+ATV and TDF/FTC+ATV/r, respectively.

### Virology Results

HIV-1 genotypic and phenotypic analysis was performed if a patient met confirmed virologic failure by having an HIV-RNA ≥400 copies/mL on 2 consecutive occasions. Confirmed virologic failure occurred in 2 patients switched to ABC/3TC+ATV and 1 patient continuing on TDF/FTC+ATV/r. Virus from 2 of the patients (1 on ABC/3TC+ATV and 1 on TDF/3TC+ATV/r) had full phenotypic susceptibility to all drugs tested, with no reverse transcriptase mutations and a few minor protease mutations (D60E from the patient on ATC/3TC+ATV/r; I62V, L10I, and V77I from the patient on TDF/FTC+ATV/r). The second patient taking ABC/3TC+ATV experienced initial rebound at Week 2 with virologic failure confirmed at Week 4, concomitant with site reports of the patient experiencing adherence issues. Virus from this patient had reduced phenotypic susceptibility to multiple drugs, including ABC, 3TC and ATV, and numerous reverse transcriptase (M41L, L74V, K103N, M184V, L210W, and T215Y) and protease (L10F, K20I, M46I, I54V, I62V, L63P, A71T, G73T, I84V, and L90M) mutations. The patient reported previous treatment with nevirapine plus TDF/FTC for >8 years before switching to TDF/FTC+ATV/r, but prior viral resistance was unknown because the patient had no HIV-1 genotypes available for review at screening.

### Safety and Tolerability Results

Rates of AEs of moderate or greater severity (grade 2–4) were similar between the two groups (40% [79/199] for ABC/3TC+ATV and 37% [36/97] for TDF/FTC+ATV/r; Table 2), with only upper respiratory tract infection observed in ≥5% of patients in either treatment group (4% [5/199] for ABC/3TC+ATV and 6% [6/97] for TDF/FTC+ATV/r). There were few grade 2–4 treatment-related AEs in either group (8% [16/199] for ABC/3TC+ATV and 5% [6/97] for TDF/FTC+ATV/r). Five patients experienced grade 2–4 cardiac disorders, but none were considered to be drug related. In the TDF/FTC+ATV/r group, one patient had grade 2 palpitations. In the ABC/3TC+ATV group, two patients had grade 2 cardiomyopathy, one patient had grade 2 coronary artery disease, and one patient (a former smoker with diabetes, hyperlipidemia and a family history of cardiovascular disease) had a grade 4 acute inferior myocardial infarction.

**Table 2:** Adverse events (AEs) and laboratory abnormalities.

	ABC/3TC+ATV (N = 199)	TDF/FTC+ATV/r (N = 97)
	n (%)	n (%)
**Grade 2–4 AEs**		
Any	79 (40)	36 (37)
**Occurring in ≥5% of patients in either treatment group**		
Upper respiratory tract infection	7 (4)	6 (6)
**Treatment related** *	16 (8)	6 (6)
Nausea	4 (2)	2 (2)
Abnormal dreams	2 (1)	0
Dizziness	2 (1)	0
**Severe or grade 3–4 AEs**		
Any†	21 (11)	10 (10)
**Treatment related**	0	2 (2)
Blood bilirubin increased	0	1 (1)
Lipase increased	0	1 (1)
**Serious AEs**		
Any‡	16 (8)	5 (5)
**Treatment related**	1 (1)	0
Drug hypersensitivity	1 (1)	0
**AEs leading to study withdrawal**	7 (4)	2 (2)
Rash	2 (1)	0
Nausea	2 (1)	0
**Treatment-emergent laboratory abnormalities**		
Any grade 2–4 event§	75 (38)	58 (60)
Any grade 3–4 event§	35 (13)	29 (30)
Treatment-emergent grade 3–4 events§		
Total bilirubin§	8 (4)	24 (25)
Lipase	5 (3)	2 (2)
Inorganic phosphorus	5 (3)	0
Creatine kinase	4 (2)	3 (3)
Glucose	4 (2)	1 (1)
LDL cholesterol calculation	0	2 (2)
Aspartate amino transferase	1 (<1)	0
Total neutrophils	1 (<1)	0

*Listing includes all events occurring in >1 patient.

† No single event occurred in >3 patients.

‡ No single event occurred in >2 patients.

§ *P*<0.001 using Fisher’s exact test.

AEs leading to study withdrawal affected 4% (7/197) of patients simplified to ABC/3TC+ATV and 2% (2/97) of patients continuing on TDF/FTC+ATV/r, with only rash and nausea reported in >1 patient. Suspected ABC hypersensitivity was reported for 1 subject who was not withdrawn from the study but restarted his original TDF/FTC +ATV/r regimen with resolution of signs and symptoms.

**Figure 2:**
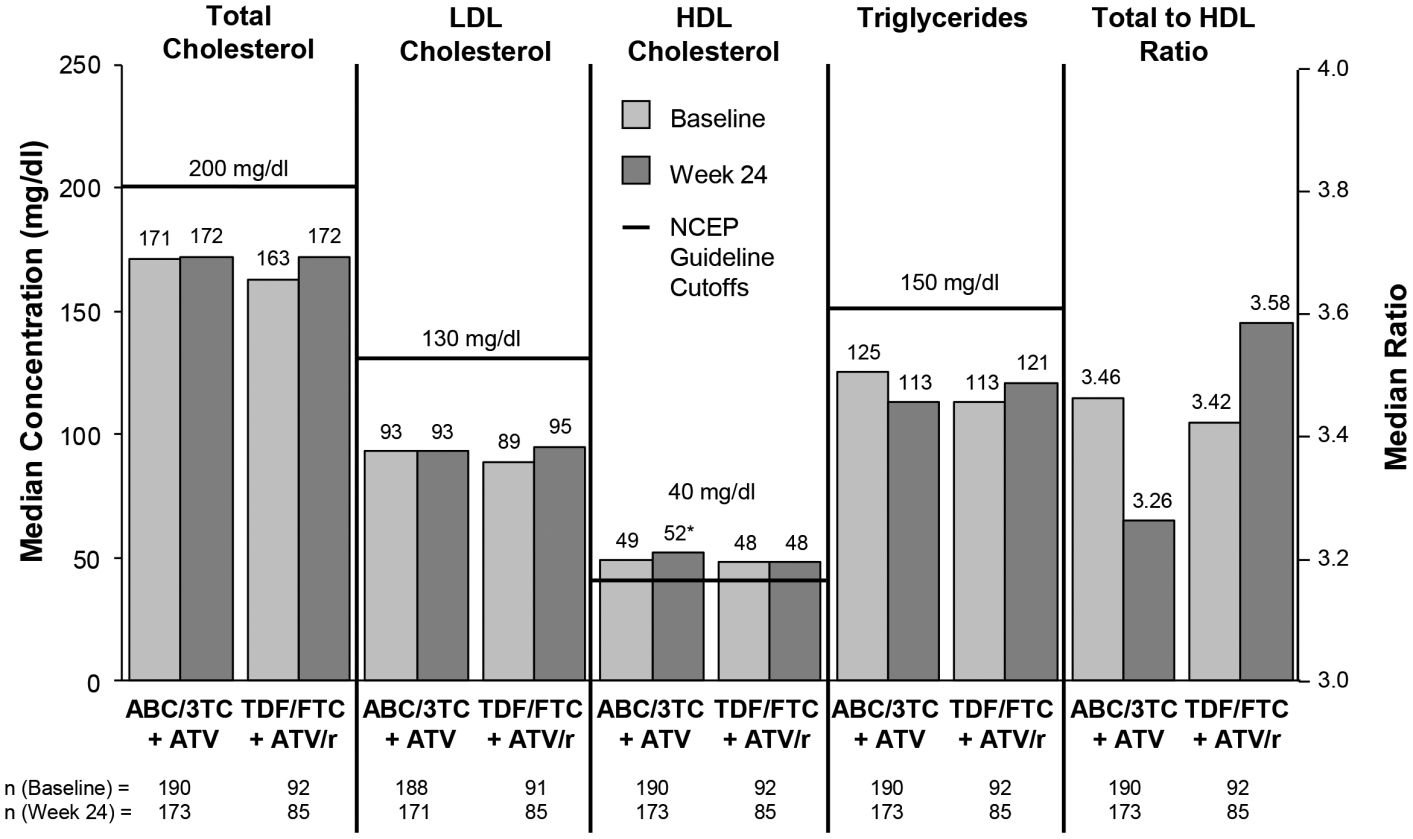
Median fasting lipid parameters with guideline cutoffs from the National Cholesterol Education Project (NCEP) [17]. *P<0.001 from baseline to Week 24.

The rate of treatment-emergent or worsening grade 2–4 and grade 3–4 laboratory abnormalities was significantly higher (*P*<0.001) in the TDF/3TC+ATV/r group compared with the ABC/3TC+ATV group. The difference was primarily driven by changes in total bilirubin; other treatment-emergent grade 3–4 lab abnormalities were infrequent and similar between groups. Fasting lipid levels (Figure 2) were similar between treatment groups and varied little between baseline and Week 24, except for fasting HDL, which increased significantly from baseline for the ABC/3TC+ATV group (median change +3 mg/dl; *P*<0.001) compared to the TDF/3TC+ATV/r group.

At baseline, the median estimated glomerular filtration rate (calculated using the MDRD equation) was 93 mL/min/1.73 m^2^ for both treatment groups. At Week 24, the rate was similar between the two groups, with a small median increase of 1 mL/min/1.73 m^2^ for the ABC/3TC+ATV group and no change from baseline in the TDF/FTC+ATV/r group. At baseline, the median 10-year risk of coronary heart disease (calculated using the Framingham equation) was 2.0% in the ABC/3TC+ATV group and 1.5% in the TDF/FTC+ATV/r group. Over 24 weeks on randomized treatment, there was no change in 10-year risk in either group.

### Biomarkers

**Figure 3:**
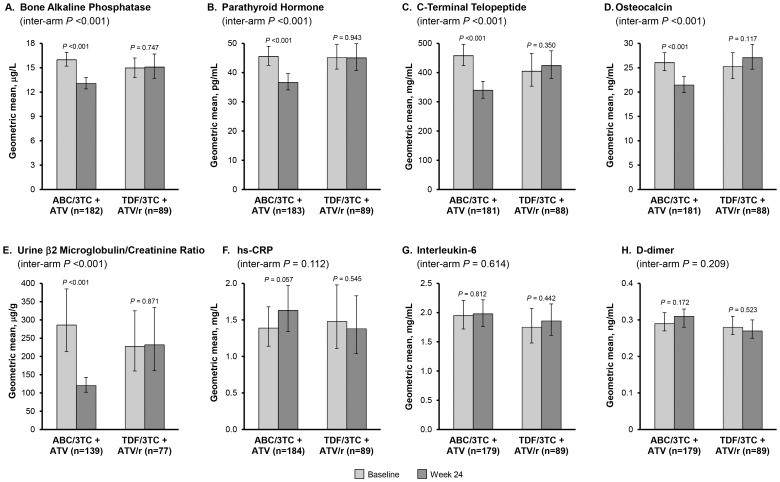
Biomarkers. Geometric means and 95% confidence intervals for bone (bone alkaline phosphatase, parathyroid hormone, C-terminal telopeptide and osteocalcin), renal (urine β2 microglobulin/creatinine ratio), and inflammatory (high sensitivity C-reactive protein [hs-CRP], interleukin-6, and d-dimer) biomarkers in patients in both treatment groups with data at baseline and Week 24. Intra-arm *P*-values were calculated using a paired t-test on the ratio of the geometric mean concentration at baseline and the geometric mean concentration at Week 24. Inter-arm *P*-values were calculated using a two sample t-test on the ratio of the geometric mean change from baseline to Week 24 in the ABC/3TC+ATV vs. TDF/FTC+ATV/r groups.

Between baseline and Week 24, the within-group decrease (improvement) in all 4 measured bone biomarkers (bone alkaline phosphatase, parathyroid hormone, C-terminal telopeptide and osteocalcin) was significant (*P*<0.001) for patients taking ABC/3TC+ATV (Figure 3A–D). These same biomarkers were relatively unchanged in patients taking TDF/FTC+ATV/r over the same time period. Comparing the two treatment groups, the decrease from baseline to Week 24 in all 4 bone biomarkers was significantly larger (*P*<0.001) for ABC/3TC+ATV than for TDF/FTC+ATV/r. Similarly, the renal biomarker urine β2 microglobulin/creatinine ratio declined by 58% in the ABC/3TC+ATV group (*P*<0.001) but remained unchanged in the TDF/FTC+ATV/r group (Figure 3E); the change from baseline to Week 24 was significantly different (*P*<0.001) between treatment groups. Conversely, there were no significant changes from baseline to Week 24 in any of the biomarkers associated cardiovascular disease, inflammation or thrombogenesis that were evaluated (hs-CRP, interleukin-6 and D-dimer) either within or between treatment groups. (Figure 3F–H).

## Discussion

### Efficacy and Safety

In this randomized, multicenter study, the percentage of patients maintaining suppression of HIV-1 RNA below 50 copies/mL at Week 24 was not different between the group simplifying to ABC/3TC+ATV and the group continuing on TDF/3TC+ATV/r (87% vs. 87%, respectively, by TLOVR analysis), and as the two sided 95% CI (−7.97% to 8.64%) stratified by prior ART excluded the predefined non-inferiority margin of −12%, non-inferiority of the simplification regimen was established. Confirmed virologic failure >400 copies/mL occurred in 1% of patients in both groups. The mean increase in CD4+ cell count from baseline to Week 24 was significantly larger for ABC/3TC+ATV (+48 cells/µL) than TDF/FTC+ATV/r (+8 cells/µL).

There were few grade 2–4 treatment-related AEs in either group, but patients treated with TDF/FTC+ATV/r had significantly more treatment-emergent or worsening grade 2–4 and grade 3–4 laboratory abnormalities than patients taking ABC/3TC+ATV – largely driven by a higher rate of hyperbilirubinemia in the RTV-containing group. Fasting lipid levels were similar between treatment groups and varied little between baseline and Week 24, except for a significant increase in HDL in patients taking ABC/3TC+ATV.

While induction-maintenance strategies in which RTV is discontinued once viral suppression has been achieved have been found to be effective and are now endorsed by DHHS guidelines [5]–[14], these studies are different in design from the registrational studies used to establish the efficacy and tolerability of the fixed dose combination of ABC/3TC where viremic ART-naïve, HIV-1 infected patients and viremic ART-experienced, HIV-1 infected patients were evaluated. This study evaluated the ability to maintain virologic suppression following RTV discontinuation in an ART-experienced population with HIV-RNA<75 c/mL and currently receiving RTV-containing therapy. The efficacy and tolerability of ABC/3TC+ATV demonstrated in this study complement those of the ARIES trial, which enrolled treatment-naïve patients to receive 36 weeks of ABC/3TC+ATV/r before randomizing 419 patients with HIV-1 RNA<50 copies/mL to maintain or discontinue RTV [13]. Twenty-four weeks after randomization in that study, viral suppression was maintained in 93% (195/210) of patients taking ABC/3TC+ATV and 89% (184/209) taking ATC/3TC+ATV/r (by TLOVR). Other data were reported only after 48 weeks on randomized therapy. Virologic failure was uncommon (2% overall), but 7 of 8 patients meeting the criteria for virologic failure were taking ATV/r. CD4+ cell responses were robust and similar between treatment groups. Few adverse events were reported after randomization: in the group discontinuing RTV, the rate of hyperbilirubinemia decreased from 14% to 4%. Fasting total cholesterol, LDL cholesterol, and triglycerides increased while patients were taking ATV/r, and values returned only partway to baseline when RTV was discontinued. Median HDL cholesterol improved from 38 mg/dl at baseline to 48 mg/dl at Week 84 regardless of randomized treatment.

Likewise, in the InduMA study, 252 antiretroviral-naïve patients received 2 NRTIs plus ATV/r for 26 to 30 weeks [8]. Patients with confirmed HIV-1 RNA<50 copies/mL were randomized to switch to unboosted ATV (n = 87) or maintain ATV/r (n = 85); NRTI backbones were not changed (approximately 52% ABC/3TC and 32% lamivudine/zidovudine). After 48 weeks on randomized therapy (no data were reported at 24 weeks), there was no significant difference in the percentage of patients with HIV-1 RNA<50 copies/mL (78% ATV and 75% ATV/r in an ITT non-completion = failure analysis). Virologic failure rates were higher than in our study (13% ATV; 7% ATV/r), but only a few patients had HIV-1 RNA >400 copies/L (5% ATV; 1% ATV/r). CD4+ cell counts continued to increase from randomization to the end of the 48-week maintenance phase, with no difference between the ATV (+92 cells/µL) and ATV/r (+100 cells/µL) groups. During the maintenance phase, the prevalence of hyperbilirubinemia decreased from 32% to 16% in the ATV group and from 32% to 28% in the ATV/r group. Fasting lipids favored ATV, with significant improvements in total cholesterol and triglycerides compared with ATV/r over 48 weeks on randomized therapy. HDL cholesterol improved in both groups, and LDL cholesterol levels were stable.

### Biomarkers

#### Bone

We found a significant improvement in markers of bone turnover following the switch to ABC/3TC+ATV but observed little change in the group maintaining TDF/FTC+ATV/r. It has previously been demonstrated that the initiation of ARV therapy is often associated with decreases in BMD that slow or stabilize over 24 to 48 weeks of therapy, and that these losses tend to be larger with TDF-containing regimens compared with non-TDF-containing regimens [18]–[21]. Several studies of bone biomarkers have been performed in treatment-naïve patients initiating therapy [21]–[24] but, to our knowledge, only 2 other randomized trials have included treatment-experienced adults [25], [26]. Results from these two trials were generally consistent with our findings that bone biomarkers were more favorable in patients taking ABC/3TC compared with TDF/FTC.

#### Renal

While the association between TDF and renal impairment is well studied, (for example, [27]–[29]) only one other randomized trial has included urine β2 microglobulin/creatinine ratio, a validated marker of combined glomerular and tubular health. ASSERT was open-label study comparing ABC/3TC with TDF/FTC, both in combination with efavirenz, in treatment-naïve patients [30]. After 24 weeks on therapy, the β2 microglobulin/creatinine ratio increased by 24% in the TDF/FTC group and decreased by 28% in the ABC/3TC group. The difference was statistically significant (*P*<0.0001) and consistent with the results observed in our study (albeit in treatment-naive patients).

#### Cardiovascular/inflammation

In our study, switching to ABC/3TC+ATV was not associated with significant changes in interleukin-6, hs-CRP or d-dimer, markers of inflammation and coagulation. Other data regarding changes in these markers following administration of ABC been mixed. Several studies found no increase in markers of cardiovascular disease with initiation of ABC compared to other NRTIs, [25], [26], [29], [31]–[35] while a metabolic substudy of AIDS Clinical Trials Group (ACTG) study A5202 reported a significant increase in hs-CRP at 24 and 96 weeks in treatment naïve patients randomized to ABC/3TC vs those randomized to TDF/FTC [20]. Interleukin-6 levels declined with both ABC/3TC and TDF/FTC, but the change was significantly smaller for those receiving ABC/3TC at week 24 but not week 96. Four other studies have examined cardiovascular markers in treatment-experienced patients: STEAL [25], SWAP [26], BICOMBO [32], and SWIFT [36]. As in our study, none of these trials reported a difference between ABC/3TC and TDF/FTC in hs-CRP, interleukin-6, or d-dimer over 48 weeks of post-randomization treatment.

There are several limitations of this trial that should be considered when interpreting these results. The study enrolled therapy-experienced patients who achieved viral suppression and had not experienced prior virologic failure leading to selection of drug resistance mutations; extrapolating these findings to other patient populations may be inappropriate. Plasma concentrations of ATV in the absence of RTV boosting are lower, and therefore this regimen may be more susceptible to patient adherence issues. Additionally, this simplification treatment strategy is restricted to certain NRTI backbones such as ABC/3TC that can be used in combination with unboosted ATV. Lastly, 24 week data are presented and the efficacy and safety of this simplification strategy needs to be evaluated over a longer period of time (this study continues to 48 weeks and follow-up results will be forthcoming).

### Conclusion

Over 24 weeks, simplification of TDF/FTC+ATV/r to ABC/3TC+ATV maintained viral suppression, was well-tolerated, and led to improvements in CD4+ cell count, bone biomarkers, renal biomarkers, and HDL cholesterol without causing increases in other fasting lipid levels or in cardiovascular biomarkers of inflammation and thrombogenesis. This study continues through 48 weeks to provide longer-term data regarding the efficacy and safety of this ART simplification strategy.

## Supporting Information

Checklist S1 **Checklist S1 is the CONSORT study checklist and lists the manuscript sections where details regarding the checklist items can be found.**
(PDF)

Protocol S1 **The complete EPZ113734 clinical study trial protocol.**
(PDF)
