# Correction: Simplification to Abacavir/Lamivudine + Atazanavir Maintains Viral Suppression and Improves Bone and Renal Biomarkers in ASSURE, a Randomized, Open Label, Non-Inferiority Trial

**DOI:** 10.1371/journal.pone.0103925

**Published:** 2014-07-23

**Authors:** The PLOS ONE Staff

There is an error in the Materials and Methods section. In the first sentence of the subheading “Design and Intervention”, the ATV dosage is incorrect. The correct ATV dosage is 400 mg. Please note that the dosage is correct in the Supporting Information protocol.
