# Supplementary material for: Simplification to Abacavir/Lamivudine + Atazanavir Maintains Viral Suppression and Improves Bone and Renal Biomarkers in ASSURE, a Randomized, Open Label, Non-Inferiority Trial
Source: PLoS One. 2014 May 13;9(5):e96187. doi: 10.1371/journal.pone.0096187 (PMC4019479; doi:10.1371/journal.pone.0096187)
Supplement: Protocol S1 — The complete EPZ113734 clinical study trial protocol. (PDF) [file pone.0096187.s002.pdf]

Division: Worldwide Development
Retention Category: GRS019
Information Type: Protocol Amendment

**Title:**	A Prospective, Randomized, Multicenter, Open-Label Study to Compare the Efficacy and Safety of Simplifying from a Regimen of Atazanavir + Ritonavir + Tenofovir/Emtricitabine to Atazanavir + Abacavir/Lamivudine Without Ritonavir for 48 Weeks in Virologically Suppressed, HIV-1 Infected, HLA-B*5701 Negative Subjects

Compound Number: GI265235+GR109714

Effective Date: 07-SEP-2010

Protocol Amendment Number: 01

Description:

The objective of this study is to evaluate the efficacy, safety, and tolerability of the antiviral response between atazanavir + ritonavir (ATV/RTV) + tenofovir/emtricitabine (TDF/FTC) or atazanavir (ATV) + abacavir sulfate/lamivudine (ABC/3TC) without ritonavir over 48 weeks in HIV-1 infected, HLA-B*5701 negative subjects previously suppressed on ATV/RTV + TDF/FTC. This study is designed to address an unmet patient need for alternative treatment strategies that do not require the long-term use of RTV. A simplification strategy is being investigated to evaluate improvement in tolerability and safety while maintaining virologic efficacy.

This study will enroll virologically suppressed subjects who are on an ART regimen of ATV/RTV + TDF/FTC as their initial regimen OR as their first or second switch regimen. Subjects should have been on this regimen for at least 6 months. These subjects will be randomized to continue on this treatment regimen or simplify to a RTV-sparing regimen, ATV + ABC/3TC.

A minimum of 300 subjects from participating sites in North America will be stratified according to their previous antiretroviral therapy experience and randomized (2:1) to receive one of the following two treatment regimens:

<u>Treatment Arm A: (Simplification)</u>	ATV 400 mg once daily + ABC/3TC 600 mg/300 mg once daily
<u>Treatment Arm B: (Continuation)</u>	ATV/RTV 300 mg/100 mg once daily + TDF/FTC 300 mg/200 mg once daily

Copyright 2010 the GlaxoSmithKline group of companies. All rights reserved. Unauthorised copying or use of this information is prohibited.

The primary endpoint is the proportion of subjects with HIV-1 RNA <50 copies/mL at Week 24 by time to loss of virologic response (TLOVR) analysis. Secondary endpoints include clinical and laboratory adverse events, virologic and immunologic responses, and detection of genotypic/phenotypic resistance at failure. Exploratory endpoints include evaluation of long term side effects of ARVs and chronic HIV infection by various neurocognitive, bone, cardiac and renal biomarkers.

Virologic failure is defined as confirmed plasma HIV-1 RNA rebound ≥400 copies/mL.

Subject: HIV Infection, EPZICOM®, KIVEXA®, atazanavir, ritonavir, tenofovir, HLA-B*5701, simplification.

Author:

[redacted]

Revision Chronology:

RM2009/00488/00	2010-FEB-12	Original
RM2009/00488/01	2010-SEP-07	Amendment No.: 01: Expand allowable pre-study ART exposure in inclusion criteria number 2, change viral load entry threshold to VL ≤75 c/ml in inclusion criteria number 3, addition of exclusion criteria for previous abacavir exposure, addition of pregnancy testing to Week 48 and early Withdrawal visit, update Withdrawal criteria, add collection of nadir CD4 value, addition of pre-baseline neurocognitive evaluation

[redacted] [redacted]

[redacted]

SPONSOR INFORMATION PAGE

Clinical Study Identifier: EPZ113734

This study is sponsored by ViiV Healthcare. GlaxoSmithKline is responsible for implementing and managing all aspects of the study.

ViiV Healthcare
Five Moore Drive
P.O. Box 13398
Research Triangle Park, NC 27709-3398, USA
Telephone: + 001-877-844-8872 (1-877-ViiVUSA)

GlaxoSmithKline
Five Moore Drive
P.O.Box 13398
Research Triangle Park, NC 27709-3398, USA
Telephone: + 001-919-483-2100

[redacted]

[redacted]

Regulatory Agency Identifying Number(s): EPZICOM (IND 63,468)

[redacted]

[redacted]

[redacted]

[redacted]

[redacted]

[redacted] [redacted]

TABLE OF CONTENTS

PAGE

LIST OF ABBREVIATIONS ... 9

PROTOCOL SUMMARY ... 12

1. INTRODUCTION ... 18
   1.1. Background ... 18
   1.2. Rationale ... 20

2. OBJECTIVES ... 21
   2.1. Primary Objective ... 21
   2.2. Secondary Objectives ... 21
   2.3. Exploratory Objectives ... 21

3. INVESTIGATIONAL PLAN ... 22
   3.1. Study Design ... 22
   3.2. Protocol-Definition of Virologic Failure ... 23
      3.2.1. Algorithm for Virologic Failure Management ... 24
      3.2.2. Withdrawal Criteria for Subjects with Confirmed Virologic Failure ... 25
   3.3. Discussion of Design ... 25

4. SUBJECT SELECTION AND WITHDRAWAL CRITERIA ... 26
   4.1. Number of Subjects ... 26
   4.2. Inclusion Criteria ... 26
   4.3. Exclusion Criteria ... 27
   4.4. Subject Withdrawal ... 28
      4.4.1. Subject Withdrawal from Study ... 28
      4.4.2. Management of Withdrawn Subjects ... 29

5. STUDY TREATMENTS ... 29
   5.1. Investigational Product ... 29
   5.2. Permitted Alternative Regimens ... 31
   5.3. Treatment Assignment ... 31
   5.4. Blinding ... 32
   5.5. Product Accountability ... 32
   5.6. Treatment Compliance ... 32
   5.7. Concomitant Medications and Non-Drug Therapies ... 33
      5.7.1. Permitted Medications and Non-Drug Therapies ... 33
      5.7.2. Prohibited Medications and Non-Drug Therapies ... 33
   5.8. Treatment after the End of the Study ... 33
   5.9. Treatment of Investigational Product Overdose ... 34

6. STUDY ASSESSMENTS AND PROCEDURES ... 34
   6.1. Time and Events Schedule ... 36
   6.2. Efficacy ... 38
      6.2.1. Primary Endpoint ... 38
      6.2.2. Secondary Efficacy Endpoints ... 38
      6.2.3. Efficacy Evaluations ... 38

6.3. Safety 38
6.3.1. Safety Endpoints..38
6.3.2. Safety Evaluations 39
6.3.2.1. Clinical Laboratory Assessments...............................39
6.4. Exploratory 40
6.4.1. Exploratory Endpoints..40
6.4.2. Exploratory Evaluations 40
6.4.3. General Toxicity Management 41
6.4.3.1. Grade 1 or Grade 2 Toxicity/Adverse Event 41
6.4.3.2. Grade 3 Toxicity/Adverse Event 41
6.4.3.3. Grade 4 Toxicity/Adverse Event 42
6.4.4. Specific Toxicities/Adverse Event Management...........................42
6.4.4.1. Abacavir Hypersensitivity Reaction.............................42
6.4.4.1.1. Risk Factors for Abacavir Hypersensitivity Reaction 43
6.4.4.1.2. Clinical Description of the Hypersensitivity Reaction 43
6.4.4.1.3. Clinical Management of Hypersensitivity Reactions......................44
6.4.4.1.4. Special considerations following an interruption of abacavir therapy..........45
6.4.4.1.5. Essential patient information..................45
6.4.4.1.6. Reporting of Hypersensitivity Reactions 46
6.4.4.1.7. Therapy Management for Abacavir Hypersensitivity Reaction 46
6.4.4.2. Stevens Johnson Syndrome, Toxic Epidermal Necrolysis or Erythema Multiforme.............................46
6.4.4.3. Rash Not Accompanied by Systemic Symptoms 46
6.4.4.4. Gastrointestinal Toxicity 48
6.4.4.5. Laboratory Toxicity 48
6.4.4.6. Renal Toxicity..50
6.4.5. Adverse Events..51
6.4.5.1. Definition of an AE...51
6.4.5.2. Definition of a SAE 52
6.4.6. Laboratory and Other Safety Assessment Abnormalities Reported as AEs and SAEs...53
6.4.7. Disease-Related Events and/or Disease-Related Outcomes Not Qualifying as SAEs...53
6.4.8. Pregnancy 54
6.4.8.1. Pregnancy Testing...54
6.4.8.2. Action to be Taken if Pregnancy Occurs.....................54
6.4.8.3. Time Period for Collecting Pregnancy Information 54
6.4.9. Time Period and Frequency of Detecting AEs and SAEs.............55
6.4.10. Prompt Reporting of Serious Adverse Events and Other Events 55
6.4.10.1. Regulatory reporting requirements for SAEs 56
6.5. Exploratory Biomarker Assessments 56
6.6. Viral Genotyping and Phenotyping..56

6.7. Pharmacogenetics 57
6.7.1. HLA-B*5701 Determination 57

7. DATA MANAGEMENT 57

8. DATA ANALYSIS AND STATISTICAL CONSIDERATIONS 58
8.1. Hypotheses 58
8.2. Study Design Considerations 58
8.2.1. Sample Size Assumptions 58
8.2.2. Sample Size Sensitivity 58
8.2.3. Sample Size Re-estimation 59
8.3. Data Analysis Considerations 59
8.3.1. Analysis Populations 59
8.3.2. Analysis Data Sets 60
8.3.3. Treatment Comparisons 60
8.3.3.1. Primary Comparisons of Interest 60
8.3.4. Interim Analysis 60
8.3.5. Key Elements of Analysis Plan 61
8.3.5.1. Efficacy Analyses 61
8.3.5.2. Safety Analyses 62
8.3.5.3. Viral Genotyping/Phenotyping Analyses 64

9. STUDY CONDUCT CONSIDERATIONS 64
9.1. Posting of Information on Clinicaltrials.gov 64
9.2. Regulatory and Ethical Considerations, Including the Informed Consent Process 64
9.3. Quality Control (Study Monitoring) 65
9.4. Quality Assurance 65
9.5. Study and Site Closure 65
9.6. Records Retention 66
9.7. Provision of Study Results to Investigators, Posting to the Clinical Trials Register and Publication 66

10. REFERENCES 68

11. APPENDICES 70
11.1. Appendix 1: CDC Classification CDC Classification System for HIV-1 Infections (1993) 70
11.2. Appendix 2: CogState 73
11.3. Appendix 3: DAIDS Division of Aids Table for Grading the Severity of Adult and Pediatric Adverse Events 74
11.4. Appendix 4: Pharmacogenetics 103
11.5. Appendix 5: Protocol Changes 109

LIST OF ABBREVIATIONS

3TC	Lamivudine, EPIVIR®
ABC	Abacavir sulfate, ZIAGEN®
AE	Adverse Event
AIDS	Acquired Immunodeficiency Syndrome
ALT	Alanine aminotransferase (SGPT)
ART	Antiretroviral Therapy
AST	Aspartate aminotransferase (SGOT)
ATV	Atazanavir
β-HCG	Beta Human Chorionic Gonadotropin
BID	Twice-daily
BSAP	Bone-Specific Alkaline Phosphatase
BUN	Blood Urea Nitrogen
c/mL	Copies per milliliter
CDC	Center for Disease Control
CI	Confidence Interval
CPK	Creatine Phosphokinase
CrCl	Creatinine Clearance
eCRF	Electronic Case Report Form
CRP	C-Reactive Protein
DHHS	Department of Health and Human Services
dL	deciliter
DNA	Deoxyribonucleic Acid
FC	Fold change
FDA	Food and Drug Administration
FDC	Fixed dose combination
FPV	Fosamprenavir, LEXIVA®
FTC	Emtricitabine
g	Gram
GCP	Good Clinical Practice
G-CSF	Granulocyte Colony-Stimulating Factor
GFR	Glomerular Filtration Rate
GSK	GlaxoSmithKline
HAART	Highly Active Antiretroviral Therapy
HbSAg	Hepatitis B Surface Antigen
hCRP	Human C-Reactive Protein
HCV	Hepatitis C Virus
HDL	High density lipoprotein
HIV-1	Human Immunodeficiency Virus, type 1
HLA-B	Human Leukocyte Antigen B
HSR	Hypersensitivity Reaction
IAS	International AIDS Society
ICF	Informed Consent Form
IEC	Independent Ethics Committee
IL	Interleukin

IND	Investigational New Drug
INR	International Normalized Ratio
IRB	Institutional Review Board
ITT	Intent-to-Treat
ITT-E	Intent-to-Treat Exposed
IUD	Intrauterine Device
Kg	Kilogram
LDL	Low density lipoprotein
LFT	Liver Function Test
LPV	Lopinavir
LSLV	Last Subject Last Visit
MCV	Mean Corpuscle Volume
MDRD	Modification of Diet in Renal Disease
MedDra	Medical Dictionary for Drug Regulatory Affairs
mm^3	Cubic millimeter
mEq	milliequivalent
M=F	Missing = Failure
Mg	Milligram
mL	Milliliter
MSDS	Material Safety Data Sheet
NNRTI	Non-Nucleoside Reverse Transcriptase Inhibitor
NRTI	Nucleoside Reverse Transcriptase Inhibitor
PI	Protease Inhibitor
PGx	Pharmacogenetics
PINP	Procollagen Type I N Propeptide
PK	Pharmacokinetics
PRTD	Proximal Renal Tubular Dysfunction
PTH	Parathyroid Hormone
QD	Once-daily
RAMOS	Registration and Medication Ordering System
RBC	Red Blood Cell
RNA	Ribonucleic Acid
RTV	Ritonavir, Norvir, /r
SAE	Serious Adverse Event
SNP	Single Nucleotide Polymorphism
SPM	Study Procedures Manual
$t_{1/2}$	Half-life
TDF	Tenofovir
TLOVR	Time to Loss of Virologic Response
ULN	Upper Limit of Normal
US	United States
vs.	Versus
WBC	White Blood Count
W/D	Withdrawal
ZDV	Zidovudine, RETROVIR®

Trademark Information

Trademarks of ViiV Healthcare
COMBIVIR
EPIVIR
EPZICOM
KIVEXA
LEXIVA
RETROVIR
TRIZIVIR
ZIAGEN

Trademarks not owned by ViiV Healthcare
Norvir
Reyataz
Truvada

PROTOCOL SUMMARY

Rationale

Protease inhibitor (PI)-based therapies containing low-dose Ritonavir (RTV) have demonstrated favourable efficacy, safety, and barrier to resistance among antiretroviral naïve and treatment experienced patients. However, RTV is associated with long-term gastrointestinal and metabolic toxicities, added pill burden and cost.

Atazanavir (ATV) is the only currently licensed PI that can be safely administered with or without RTV. Several trials investigating an induction-simplification treatment strategy to achieve rapid initial viral load reduction with RTV-boosted ATV followed by simplification to unboosted ATV have demonstrated comparable efficacy. Although there are limited published data utilizing this strategy in clinical practice, a RTV-sparing strategy with ATV has shown improvement in lipids, bilirubin, low rate of virologic failure, and patient acceptance based on improved tolerability and fewer side effects.

The need to find alternative treatment strategies that do not require the use of RTV is of continued interest among clinicians who perceive this as an unmet patient need. Data from this study will support this simplification strategy as a long term treatment option for subjects and clinicians who prefer a RTV-sparing regimen.

The benefit to patients would be a simplified and cost effective treatment regimen, with potentially fewer adverse effects and potential reduction in drug-drug interactions. The risks to this approach are potentially lower ATV trough levels that may lead to resistance development and the unknown long-term impact of switching Tenofovir/Emtricitabine (TDF/FTC) for Abacavir/Lamivudine (ABC/3TC). However, this risk to patients may be small and mitigated due to the expected low rate of virologic failure observed following this strategy, the ability to change to an alternative treatment regimen quickly at a relatively low level of viral replication, the expected emergence of mutation pattern(s) readily rescued by other current available PIs, and the shorter study duration.

Potential risks of using this simplification study design are that some subjects may experience viral rebound and potentially develop resistance mutations rendering the simplification regimen less effective. However, subjects randomized to the simplification arm who experience viral rebound will have the opportunity to switch to an alternative regimen at the time of confirmed virologic failure. Overall the risk of virologic failure in the RTV-sparing arm is small and readily manageable compared to the benefit of protection from long-term complications of RTV-containing therapy such as hyperlipidemia, insulin resistance, lipoatrophy, and gastrointestinal intolerance and associated complexities for Human Immunodeficiency Virus (HIV)-infected patients with other co-morbidities.

The long-term implications of this strategy are currently unknown, but any risk is likely to be small given the short-term exposure to resistant virus and that ATV-resistant virus has been documented *in vitro* to be susceptible to alternative PIs.

Additionally, there is a small risk for subjects that are simplified to the ABC/3TC regimen to experience a suspected ABC hypersensitivity (HSR) reaction. However, all subjects enrolled in this study will be HLA-B*5701 negative and therefore the risk for ABC HSR will be substantially reduced as evidenced by data in other HLA-B*5701 negative subjects receiving ABC-containing regimens in the ARIES and PREDICT-1 studies.

Objectives

The primary objective is to compare the safety and efficacy of antiretroviral response among virologically suppressed, HIV-1 infected, HLA-B*5701 negative subjects who are randomized to remain on ATV/RTV + TDF/FTC or simplify to ATV + ABC/3TC over 24 weeks and continue randomized therapy for a total of 48 weeks.

Secondary objectives are to compare the safety, tolerability, and virologic efficacy between ATV + ABC/3TC and ATV/RTV + TDF/FTC over 24 and 48 weeks, evaluate the immunologic response to ATV + ABC/3TC versus ATV/RTV + TDF/FTC over 24 and 48 weeks, compare clinical and laboratory adverse events (AEs) over 24 and 48 weeks, and evaluate genotypic and phenotypic resistance patterns in subjects who experience virologic failure over 24 and 48 weeks.

Exploratory objectives are to assess and compare neurocognitive changes between ATV + ABC/3TC and ATV/RTV + TDF/FTC over 24 and 48 weeks and compare changes in serum cardiovascular, bone, and renal biomarkers over 24 and 48 weeks.

Study Design

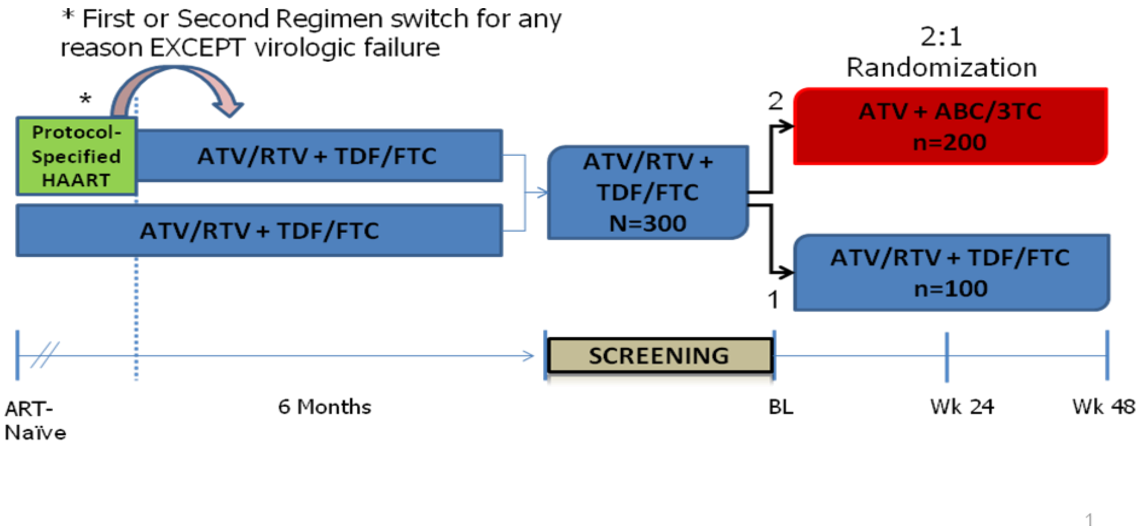

This is a phase IV, prospective, randomized, open-label, multicenter, non-inferiority study of the safety, efficacy, and tolerability of ATV + ABC/3TC once daily compared to ATV/RTV + TDF/FTC once daily for 48 weeks in HIV-1 infected, HLA-B*5701-negative subjects who are currently receiving a stable regimen of ATV/RTV + TDF/FTC once daily and are virologically suppressed (plasma HIV-1 RNA ≤75 c/mL).

A minimum of 300 subjects meeting eligibility criteria will be stratified by initial antiretroviral regimen received (ATV/RTV + TDF/FTC as initial regimen OR as first or second switch regimen) and randomized 2:1 to receive one of the following anti-retroviral therapy (ART)-regimens below for 48 weeks. Subjects will have twice the chance to be randomized to the simplification arm than the continuation arm.

**Treatment Arm A: (Simplification)**	ATV 400 mg once daily + ABC/3TC 600 mg/300 mg once daily
**Treatment Arm B: (Continuation)**	ATV/RTV 300 mg/100 mg once daily + TDF/FTC 300 mg/200 mg once daily

Subjects who enter the Screening period of this study must continue receiving their ATV/RTV + TDF/FTC regimen up to, but not including, the Baseline visit (Day 1). Subjects will begin randomized treatment on Day 1.

This study consists of a 35 day Screening period, a 48 week Treatment period (Day 1 through Week 48) and a Follow-up period (contact approximately 2-4 weeks after the Week 48 visit or Withdrawal visit).

Protocol-Defined Virologic Failure

Virologic failure is defined as plasma HIV-1 RNA rebound ≥400 c/mL.

Virologic failure in this study must be confirmed:

- A subject is considered a suspected virologic failure after the first HIV-1 RNA measurement of ≥400 c/mL.
- If the repeat HIV-1 RNA measurement performed at least 28 days later is again ≥400 c/mL, the subject is considered a confirmed virologic failure.

Algorithm for Management of Virologic Failure

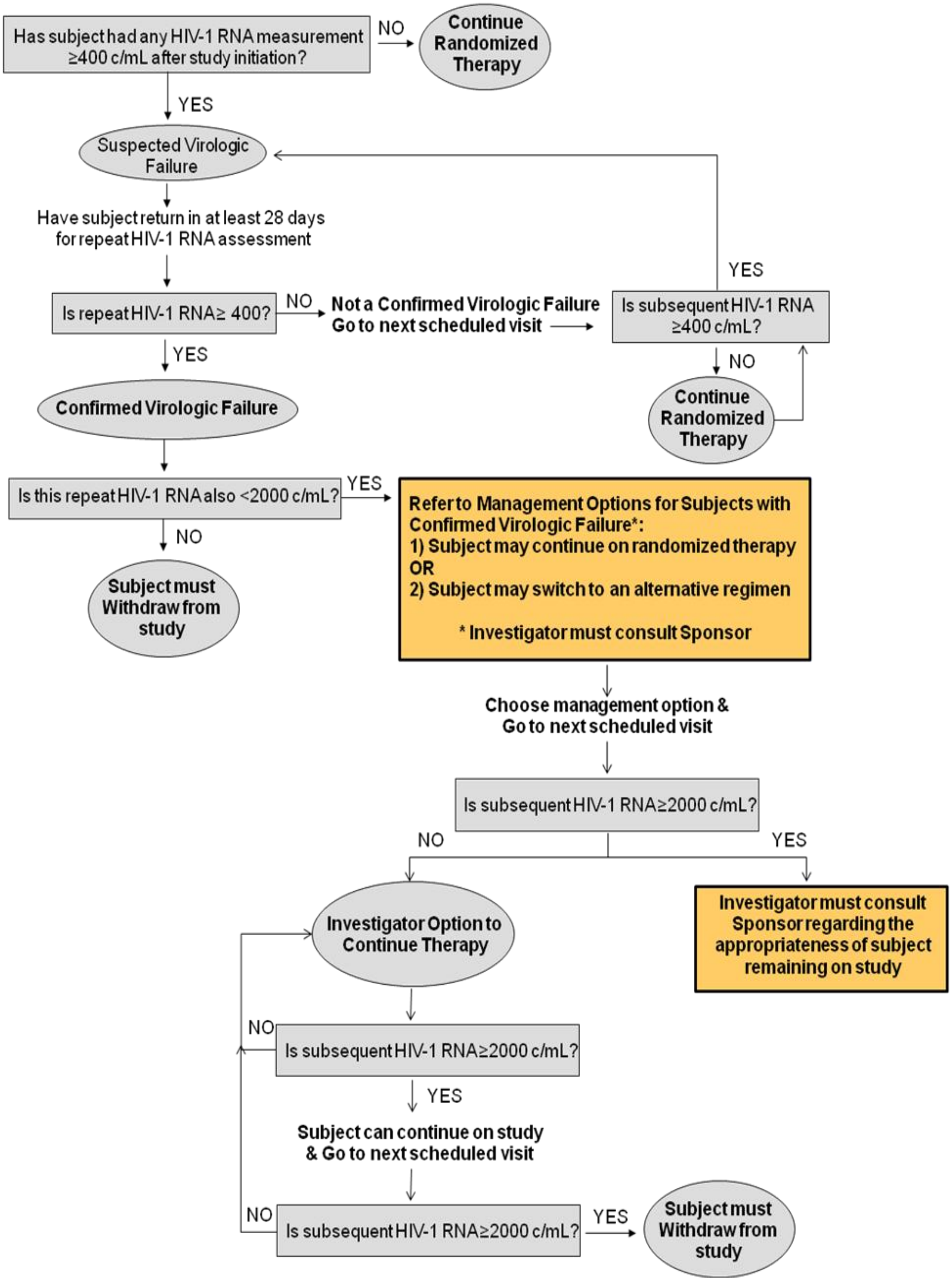

Subjects who meet the definition for confirmed virologic failure with an HIV-1 RNA measurement ≥400 but <2000 c/mL may continue in the study at the discretion of the investigator and after consultation with the Sponsor, and choose one of the two following management options:

1. Continue randomized treatment regimen
2. Change to a new antiretroviral regimen

If a subject switches to a new antiretroviral regimen, any regimen may be chosen. The Sponsor will provide the following study drugs for the purpose of constructing a new treatment regimen: ATV, RTV, ABC/3TC, TDF/FTC, 3TC/Zidovudine (ZDV) or Fosamprenavir (FPV).

Withdrawal Criteria for Subjects with Confirmed Virologic Failure

Subject must be withdrawn from the study for either of the following situations:

- Subject has an HIV-1 RNA measurement ≥2000 c/mL at the confirmatory visit for virologic failure
- Subject has two consecutive HIV-1 RNA measurements ≥2000 c/mL at any time

Note: A subject with an HIV-1 RNA ≥2000 c/mL at the time of suspected virologic failure need not be withdrawn as long as the repeat (confirmatory) HIV-1 RNA is <2000 c/mL.

Suspected Abacavir Hypersensitivity Reaction

Subjects who experience a suspected abacavir hypersensitivity reaction (ABC HSR) must discontinue abacavir and may chose from one of the following two options to remain on study:

1. Substitute zidovudine/lamivudine (ZDV/3TC) for ABC/3TC. ZDV/3TC will be provided by the Sponsor.
2. Substitute TDF/FTC for ABC/3TC and change back to original regimen of ATV/RTV + TDF/FTC. TDF/FTC and ATV/RTV will be provided by the Sponsor.

Declining Renal Function

Creatinine clearance <50 mL/min

For subjects who experience progression to an estimated creatinine clearance (CrCl) (calculated by Cockroft-Gault equation) to <50 mL/min judged by the investigator to be attributed to study medication, the offending agent(s) must be discontinued. No dose-reduction of the offending agent(s) will be allowed.

For subjects who experience progression to an estimated creatinine clearance (CrCl) (calculated by Cockroft-Gault equation) to <50 mL/min judged by the investigator to be NOT study drug-related, the investigator will choose from one of the following two management options for study medications:

1. Discontinue study medication(s) and switch to another agent in the same class and remain on study.
2. Dose reduce study medication (s) as indicated in prescribing information and remain on study.

Proximal Renal Tubule Dysfunction

Subjects who meet clinical and/or laboratory criteria for proximal renal tubule dysfunction (PRTD) must be withdrawn from study.

Study Endpoints/Assessments

Primary Endpoint:

The primary endpoint is to determine the proportion of subjects who maintain plasma HIV-1 RNA <50 c/mL at 24 weeks by the Time to Loss Of Virologic Response (TLOVR) algorithm.

Secondary Endpoints:

The secondary efficacy endpoints are to determine the following: proportion of subjects with plasma HIV-1 RNA <50 c/mL at 48 weeks, proportion with plasma HIV-1 RNA <400 c/mL at 24 and 48 weeks, change from Baseline in HIV-1 RNA and CD4+ cell count, time to virologic failure, and identification of genotypic and phenotypic resistance in virus from subjects with virologic failure.

The secondary safety endpoints are to evaluate the change from Baseline in fasting lipid profiles (total cholesterol, low density lipoprotein (LDL), high density lipoprotein (HDL), triglycerides) and incidence of Grade 2 to 4 AEs and all serious adverse events (SAE)s.

Exploratory Endpoints:

The exploratory endpoints are to assess change from Baseline in neurocognition scores measured by CogState and changes from Baseline in various cardiovascular, bone, and renal biomarkers, which may or may not include markers such as high-sensitivity C-reactive protein (hsCRP), interleukin-6 (IL- 6), D-dimer, serum procollagen type 1 N-propeptide (P1NP), serum bone specific alkaline phosphatase (BSAP), serum parathyroid hormone (PTH), serum c-telopeptide (CTx), osteocalcin, vitamin D 1,25-OH, and beta-2 microglobulin.

1. INTRODUCTION

1.1. Background

There are over twenty Food and Drug Administration (FDA) approved antiretroviral agents available with which to construct a highly active antiretroviral therapy (HAART) regimen which has proven to reduce morbidity and mortality in individuals with human immunodeficiency virus infection (HIV) or acquired immunodeficiency syndrome (AIDS) [Palella, 1998]. The current standard of therapy is a combination of at least three antiretrovirals consisting of two nucleoside/nucleotide (NRTI) analogues plus either a non-nucleoside reverse transcriptase inhibitor (NNRTI), a protease inhibitor (PI), or an integrase inhibitor [DHHS, 2009]. Among the available agents, atazanavir (ATV) boosted with ritonavir (RTV) + tenofovir/emtricitabine (TDF/FTC) is currently a preferred initial regimen for antiretroviral-naïve patients to reduce viral load and improve CD4 lymphocyte counts.

Low-dose RTV is commonly added to PI-based regimens to provide pharmacokinetic enhancement of the parent PI and to reduce the risk of drug resistance. However, RTV is also associated with adverse effects including gastrointestinal upset and lipid and metabolic alterations that may increase future cardiovascular risk. In addition, RTV is a perpetrator of cytochrome P450-mediated drug interactions which can limit the use of other concomitant drugs [Norvir Package Insert, 2010; Reyataz Package Insert, 2010]. The addition of RTV to PIs not co-formulated with RTV adds to the patient's pill burden and is associated with extra medication costs. Therefore, there is continued interest among clinicians to find alternative treatment strategies that do not require the use of RTV but continue to offer the potency and high virologic resistance barrier of protease inhibitors.

Treatment induction with a highly potent combination regimen followed by simplification is one strategy that has demonstrated comparable safety and efficacy outcomes in several studies [Gatell, 2007; Delfraissy, 2008; Squires, 2010a; Squires, 2010b]. Regimen induction with a RTV-boosted PI provides rapid initial virologic suppression reducing risk for development of viral resistance while subsequent simplification optimizes tolerability and adherence, minimizes short and long-term toxicity, and may reduce drug-drug interactions.

The SWAN study was a multicenter, randomized, open-label trial of 419 patients on a stable, virologically suppressive, PI-based regimen for a mean of 40.3 months who were randomized 2:1 to either switch to an ATV-containing regimen with unchanged NRTIs or remain on the initial non-ATV PI-based regimen. ATV was administered without RTV except in approximately 9% of subjects whose NRTIs included TDF, in which case ATV was given with low-dose RTV due to the pharmacokinetic interaction of ATV and TDF which requires that when using ATV in combination with TDF it must be boosted by RTV [Gatell, 2007]. In a post-hoc analysis among 153 subjects who were initially stable on a virologically suppressive, Lopinavir (LPV)/RTV based regimen, 82 (53%) switched to an unboosted ATV-based regimen, 18 (12%) switched to an ATV/RTV based regimen, and the remaining 53 subjects (34.5%) continued on the original LPV/RTV based

regimen. At Week 48, the rates of viral rebound were comparable; 11% in the combined ATV arm and 9% in the LPV/RTV arm [Gatell, 2006]. Rates of treatment failure for any reason, which included viral rebound, failure to receive the randomized treatment, or discontinuation of study therapy were similar in both groups (26% of patients on LPV/RTV versus 28% on ATV). In addition, subjects switched to ATV had a reduction in new onset gastrointestinal symptoms, improvements in lipid parameters, and a lower usage of lipid lowering agents compared to subjects remaining on the LPV/RTV arm [Gatell, 2006]. This post-hoc analysis demonstrated that subjects switching from a stable, virologically suppressive, LPV/RTV based regimen to a RTV-boosted or RTV-sparing ATV based regimen maintained similar virologic control with fewer lipid abnormalities and better gastrointestinal tolerability.

The ARIES study was a 144-week, phase IIIB, randomized, open-label, multi-center study in 419 randomized patients who had achieved virologic suppression during the initial 36-week induction phase. In the induction phase, patients received ATV/RTV + abacavir sulfate/lamivudine (ABC/3TC) over 36 weeks and those achieving virologic suppression were subsequently randomized (1:1) to a simplification regimen of ATV + ABC/3TC or remained on the induction regimen and then followed for 108 weeks. At week 84 (48 weeks from the start of randomization), 86% of patients randomized to the simplification arm vs. 81% of patients remaining on the induction arm maintained virologic suppression to HIV-1 RNA <50 copies/mL (c/mL) [Squires, 2010a]. Virologic failure occurred in 0.5% of patients in the simplification arm compared to 3% of patients in the induction arm through 84 weeks. Results were similar among patients with entry viral loads above and below 100,000 c/mL. Patients on the simplification regimen had less hyperbilirubinemia and a reduction in total cholesterol and triglycerides. At week 120 (84 weeks from start of randomization), virologic efficacy was sustained between groups resulting in 84% of ATV + ABC/3TC versus 83% of ATV/RTV + ABC/3TC subjects maintaining suppression to <50 c/mL [Squires, 2010b]. No differences in response rates by baseline viral load were observed between groups. The overall rate of virologic failure was 2% and comparable between groups. Subjects in the simplification arm continued to demonstrate favorable lipid benefits and maintained bilirubin reductions through 120 weeks.

ABC/3TC is a dual nucleoside combination that is also commonly used with RTV-boosted ATV, but may also be used with unboosted ATV in appropriate patient populations. Recent data have shown that the combination of unboosted ATV plus ABC/3TC effectively maintains virologic suppression (plasma HIV-1 RNA <50 c/mL) after initial induction with RTV-boosting of ATV in HIV-infected patients [Delfraissy, 2008; Squires, 2010b]. In addition, ABC/3TC has a well-established safety profile and relatively few drug interactions [EPZICOM® Package Insert, 2009].

The current study will evaluate whether subjects receiving a RTV-containing regimen of ATV/RTV + TDF/FTC who have achieved virologic suppression can safely simplify to a RTV-sparing regimen of ATV + ABC/3TC and maintain virologic suppression through 48 weeks.

This study will also evaluate changes in neurocognition (via a computerized test battery), and cardiovascular, renal and bone biomarkers as exploratory endpoints. As the life

expectancy of HIV-1 infected patients continues to improve, long-term consequences of chronic HIV-infection and potentially specific antiretroviral therapies is of active research interest. Additionally, this study will increase our knowledge regarding the incidence of clinically suspected abacavir hypersensitivity reaction (ABC HSR) after excluding patients who carry the HLA-B*5701 allele.

1.2. Rationale

Protease inhibitor (PI)-based therapies containing low-dose RTV have demonstrated favourable efficacy, safety, and barrier to resistance among antiretroviral naïve and treatment experienced patients. However, RTV is associated with long-term gastrointestinal and metabolic toxicities, added pill burden and cost.

ATV is the only currently licensed PI that can be safely administered with or without RTV. Several trials [Delfraissy, 2008; Gatell, 2007; Squires, 2010a; Squires, 2010b] investigating an induction-simplification treatment strategy to achieve rapid initial viral load reduction with RTV-boosted ATV followed by simplification to unboosted ATV have demonstrated comparable efficacy. Although there are limited published data utilizing this strategy in clinical practice, a RTV-sparing strategy with ATV has shown improvement in lipids, bilirubin, low rate of virologic failure, and patient acceptance based on improved tolerability and fewer side effects [Santos, 2009; Pavie, 2009; Giuntini, 2010].

The need to find alternative treatment strategies that do not require the use of RTV is of continued interest among clinicians who perceive this as an unmet patient need. Data from this study will support this simplification strategy as a long term treatment option for subjects and clinicians who prefer a RTV-sparing regimen.

The benefit to patients would be a simplified and cost effective treatment regimen, with potentially fewer adverse effects and potential reduction in drug-drug interactions. The risks to this approach are potentially lower ATV trough levels that may lead to resistance development and the unknown long-term impact of switching TDF/FTC for ABC/3TC. However, this risk to patients may be small and mitigated due to the expected low rate of virologic failure observed following this strategy [Squires, 2010a; Squires, 2010b; Delfraissy, 2008], the ability to change to an alternative treatment regimen quickly at a relatively low level of viral replication, the expected emergence of mutation pattern(s) readily rescued by other current available PIs, and the shorter study duration.

Potential risks of using this simplification study design are that some subjects may experience viral rebound and potentially develop resistance mutations rendering the simplification regimen less effective [Malan, 2006; McGrath, 2006]. However, subjects randomized to the simplification arm who experience viral rebound will have the opportunity to switch to an alternative regimen at the time of confirmed virologic failure. Overall the risk of virologic failure in the RTV-sparing arm is small and readily manageable compared to the benefit of protection from long-term complications of RTV-containing therapy such as hyperlipidemia, insulin resistance, lipoatrophy, and gastrointestinal intolerance and associated complexities for HIV-infected patients with other co-morbidities.

The long-term implications of this strategy are currently unknown, but any risk is likely to be small given the short-term exposure to resistant virus and that ATV-resistant virus has been documented *in vitro* to be susceptible to alternative PIs.

Additionally, there is a small risk for subjects that are simplified to the ABC/3TC regimen to experience a suspected ABC HSR reaction. However, all subjects enrolled in this study will be HLA-B*5701 negative and therefore the risk for ABC HSR will be substantially reduced as evidenced by data in other HLA-B*5701 negative subjects receiving ABC-containing regimens in the ARIES and PREDICT-1 studies [Squires, 2010a; Mallal, 2008].

2. OBJECTIVES

2.1. Primary Objective

- To demonstrate that ATV + ABC/3TC is virologically noninferior to ATV/RTV + TDF/FTC over 24 weeks in a population of virologically suppressed HIV-1 infected subjects

2.2. Secondary Objectives

- To compare the safety, tolerability, and virologic efficacy between ATV + ABC/3TC and ATV/RTV + TDF/FTC over 24 and 48 weeks
- To compare the immunologic response to ATV + ABC/3TC versus ATV/RTV + TDF/FTC over 24 and 48 weeks
- To evaluate clinical and laboratory adverse events (AEs) in subjects on ATV + ABC/3TC compared to those remaining on their Baseline regimen of ATV/RTV + TDF/FTC over 24 and 48 weeks
- To evaluate genotypic and phenotypic resistance patterns in subjects who experience virologic failure over 24 and 48 weeks

2.3. Exploratory Objectives

- To compare neurocognitive changes between ATV + ABC/3TC and ATV/RTV + TDF/FTC over 24 and 48 weeks
- To compare changes in various serum cardiovascular, bone, and renal biomarkers between ATV + ABC/3TC and ATV/RTV + TDF/FTC over 24 and 48 weeks

3. INVESTIGATIONAL PLAN

3.1. Study Design

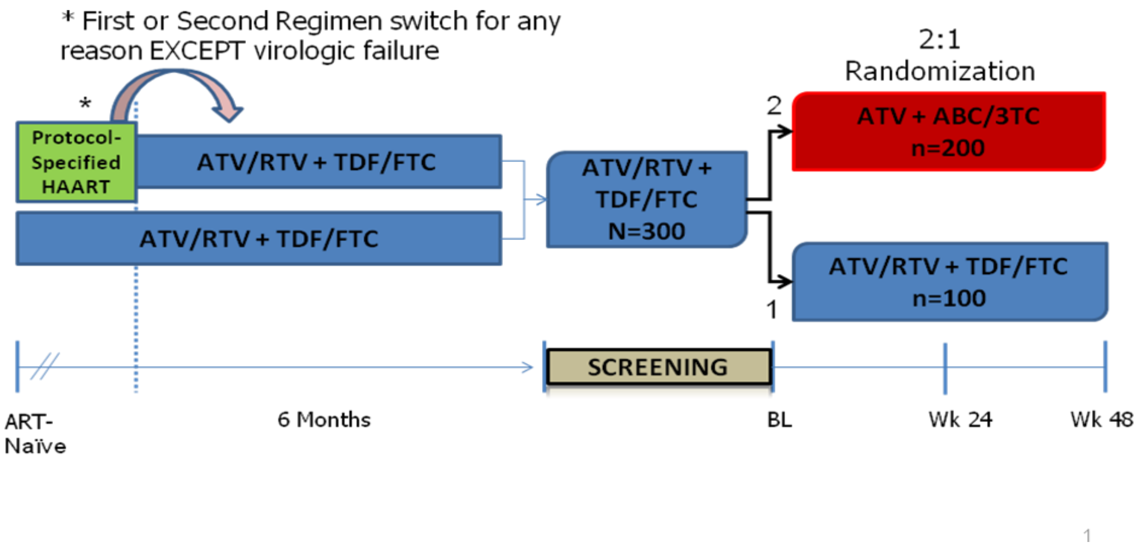

This is a phase IV, prospective, randomized, open-label, multicenter, non-inferiority study of the safety, efficacy, and tolerability of ATV + ABC/3TC once daily compared to ATV/RTV + TDF/FTC once daily for 48 weeks in HIV-1 infected, HLA-B*5701-negative subjects who are currently receiving a stable regimen of ATV/RTV + TDF/FTC once daily and are virologically suppressed (plasma HIV-1 RNA ≤75 c/mL).

ATV/RTV + TDF/FTC once daily must be the subject's initial, or first or second switch regimen. However, regimen switches must not have been due to virologic failure.

A minimum of 300 subjects meeting eligibility criteria will be stratified by initial antiretroviral regimen received (ATV/RTV + TDF/FTC as initial regimen OR as the first or second switch regimen) and randomized 2:1 to receive one of the following anti-retroviral therapy (ART)-regimens below for 48 weeks. Subjects will have twice the chance to be randomized to the simplification arm than the continuation arm.

**Treatment Arm A: (Simplification)**	ATV 400 mg once daily + ABC/3TC 600 mg/300 mg once daily
**Treatment Arm B: (Continuation)**	ATV/RTV 300 mg/100 mg once daily + TDF/FTC 300 mg/200 mg once daily

Subjects who enter the Screening period of this study must continue receiving their ATV/RTV + TDF/FTC regimen up to, but not including, the Baseline visit (Day 1). Subjects will begin randomized treatment on Day 1.

This study consists of a 35 day Screening period, a 48 week Treatment period (Day 1 through Week 48) and a follow-up period (contact approximately 2-4 weeks after the Week 48 visit or Withdrawal visit).

Study participation is considered complete when a subject has completed study procedures through Week 48.

Supplementary study conduct information not mandated to be present in this protocol is provided in the accompanying Study Procedures Manual (SPM). The SPM will provide the site personnel with administrative and detailed technical information that does not impact subject safety.

3.2. Protocol-Definition of Virologic Failure

Virologic failure is defined as plasma HIV-1 RNA rebound ≥400 c/mL.

Virologic failure in this study must be confirmed:

- A subject is considered a suspected virologic failure after the first HIV-1 RNA measurement of ≥400 c/mL.
- If the repeat HIV-1 RNA measurement performed at least 28 days later is again ≥400 c/mL, the subject is considered a confirmed virologic failure.

3.2.1. Algorithm for Virologic Failure Management

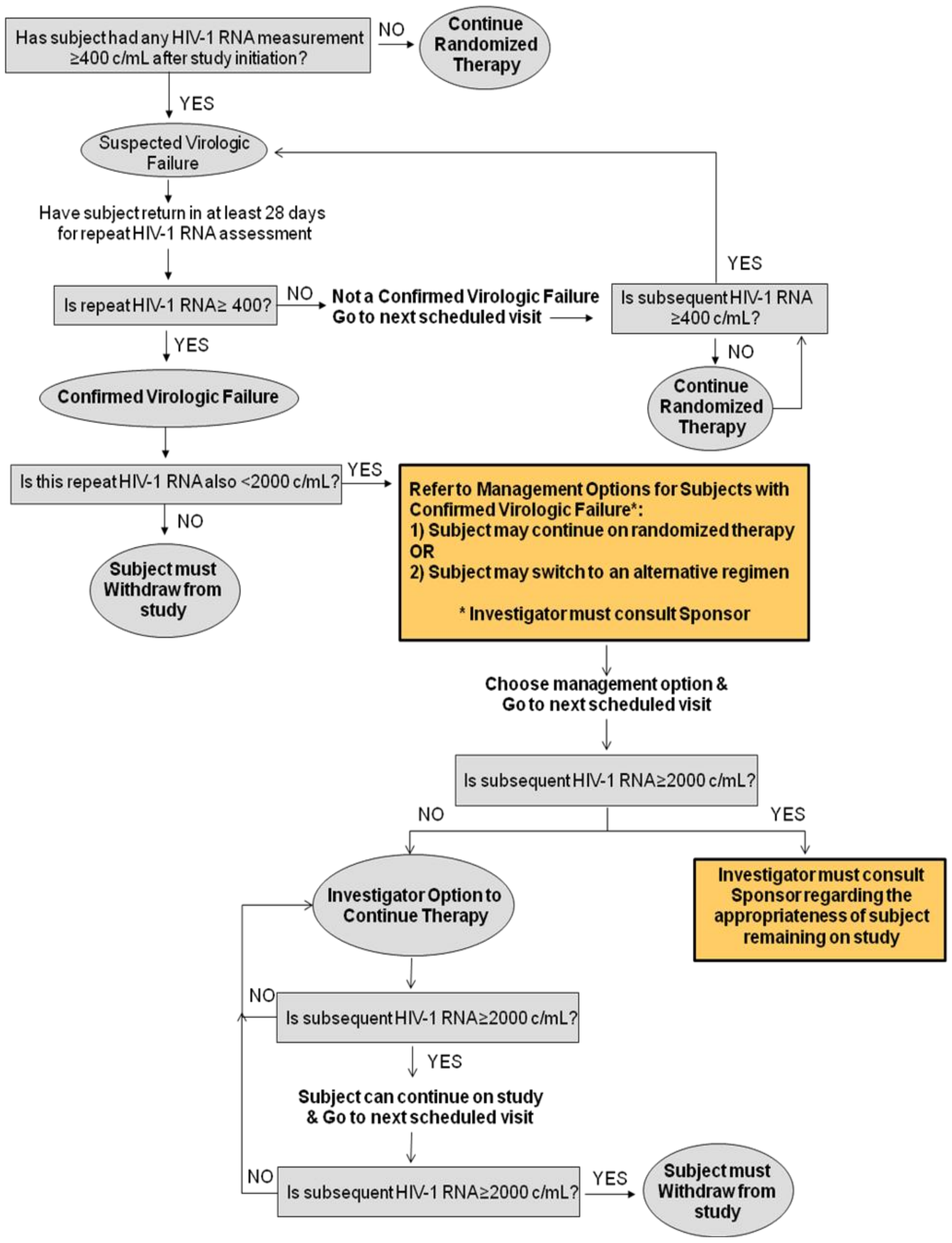

Subjects who meet the definition for confirmed virologic failure with an HIV-1 RNA measurement ≥400 but <2000 c/mL may continue in the study at the discretion of the investigator and after consultation with the Sponsor, and choose one of the following two management options:

1. Continue randomized treatment regimen.
2. Change to a new antiretroviral regimen

If a subject switches to a new antiretroviral regimen, any regimen may be chosen. The Sponsor will provide the following study drugs for the purpose of constructing a new treatment regimen: ATV, RTV, ABC/3TC, TDF/FTC, Zidovudine (ZDV)/3TC or Fosamprenavir (FPV).

3.2.2. Withdrawal Criteria for Subjects with Confirmed Virologic Failure

Subject must be withdrawn from the study for either of the following situations:

- Subject has an HIV-1 RNA measurement ≥2000 c/mL at the confirmatory visit for virologic failure (see Section 3.2)
- Subject has two consecutive HIV-1 RNA measurements ≥2000 c/mL at any time

Note: A subject with an HIV-1 RNA ≥2000 c/mL at the time of suspected virologic failure need not be withdrawn as long as the repeat (confirmatory) HIV-1 RNA is <2000 c/mL.

3.3. Discussion of Design

The current study is designed to evaluate whether virologically-suppressed subjects receiving ATV/RTV + TDF/FTC maintain virologic efficacy comparable to subjects who are simplified to ATV + ABC/3TC over 48 weeks. A simplification strategy is being investigated given the results from the ARIES trial demonstrating the safety and efficacy of switching virologically suppressed subjects from RTV-boosted ATV + ABC/3TC to unboosted ATV + ABC/3TC over 144 weeks [Squires, 2010a]. This strategy is also attractive given the potential to avoid the typical cumulative adverse effects of RTV as well as avoiding the known interaction between ATV and TDF which requires the use of RTV [Reyataz Package Insert, 2010].

An open-label instead of a placebo-matched study design was chosen since a placebo-matched design would add to the pill burden of both treatment regimens. A placebo-matched design would require that subjects randomized to the unboosted ATV treatment arm receive an additional RTV-placebo while subjects randomized to boosted ATV receive an additional ATV-placebo. RTV is associated with taste disturbances and other gastrointestinal adverse effects that would be difficult to match with a placebo in subjects accustomed to receiving RTV. Further, a placebo-matched design may be unnecessary since the primary objective is based on a virologic outcome. However, the absence of placebo-matching may be a potential source of bias in this study.

Utilizing the ABC/3TC fixed-dose combination (FDC) tablet as the NRTI backbone, this open-label, randomized, multicenter study will compare the safety and efficacy of ATV + ABC/3TC administered once daily to continuation of ATV/RTV + TDF/FTC once daily for 48 weeks in HIV-infected, HLA-B*5701 negative subjects who were initially suppressed on a combination of ATV/RTV + TDF/FTC once daily.

4. SUBJECT SELECTION AND WITHDRAWAL CRITERIA

4.1. Number of Subjects

Approximately 300 HLA-B*5701 negative, adult HIV-1 infected patients will be randomized at approximately 40 sites within North America.

4.2. Inclusion Criteria

Subjects eligible for enrolment in the study must meet all of the following criteria:

1. Adults ≥ 18 years of age.
2. Receiving a once-daily regimen of ATV/RTV (300 mg/100 mg) + TDF/FTC (300 mg/200 mg) for at least 6 months prior to the first day of Screening.
    - ATV/RTV+TDF/FTC must be the subject’s initial regimen or first or second switch regimen.
        - Initial regimen is defined as the first regimen received by a previously antiretroviral naïve subject
        - Any change of antiretroviral therapy, whether of a single drug or multiple drugs simultaneously, is considered a regimen switch.
        - Subjects must not have switched due to virologic failure.
        - If ATV/RTV + TDF/FTC is a subject’s first or second switch regimen, then the subject may only have received the following prior regimens:
            - Any currently licensed NNRTI in combination with either TDF/FTC or ZDV/3TC
            - RTV-boosted PI in combination with TDF/FTC or ZDV/3TC
            - An alternative prior regimen not listed above ONLY after consultation and assent from the Sponsor on a case-by-case basis
3. Virologically suppressed on ATV/RTV + TDF/FTC
    - Virologically suppressed is defined as HIV-1 RNA ≤75 c/mL at 2 consecutive timepoints, one of which is at Screening and the other at least 28 days prior to Screening
4. A female is eligible to enter and participate in the study if she is of:
    a. Non-childbearing potential (ie, physiologically incapable of becoming pregnant, including any female who is pre-menarchal or post-menopausal); or,

b. Child-bearing potential, has a negative pregnancy test at Screening (serum β-Human chorionic gonadotropins (HCG) and Baseline (urine β-HCG) and agrees to one of the following methods of contraception (any contraception method must be used consistently and correctly, i.e., in accordance with both the approved product label and the instructions of a physician):

- Complete abstinence from sexual intercourse from 2 weeks prior to administration of the Investigational Products, throughout the study, and for at least 2 weeks after discontinuation of all study medications
- Double barrier method (male condom/spermicide, male condom/diaphragm, diaphragm/spermicide). Hormonal contraception will not be considered adequate for inclusion into this study.
- Any intrauterine device (IUD) with published data showing that the expected failure rate is <1% per year.
- Sterilization (female subject or male partner of female subject).

All subjects participating in the study should be counselled on the practice of safer sexual practices including the use of effective barrier methods (e.g. male condom/spermicide).

4.3. Exclusion Criteria

Subjects meeting any of the following criteria must not be enrolled in the study:

1. Evidence of virologic failure at any time defined as two consecutive plasma HIV-1 RNA levels ≥200 c/mL after initial suppression to HIV-1 RNA ≤75 c/mL.
2. Any known HIV genotyping results indicating that the subject has virus containing any of the following HIV-1 mutations:
   - Reverse transcriptase mutations: K65R, K70E, L74V, M184I/V, or Y115F
   - Combination of two or more thymidine analog mutations: M41L, D67N, K70R, K219Q or E that include changes at either L210 or T215
   - Three or more of the following HIV-1 protease mutations associated with atazanavir resistance: D30, V32, M36, M46, I47, G48, I50, I54, A71, G73, V77, V82, I84, N88, and L90

   *NOTE: A Baseline genotype is NOT required to determine eligibility.
3. HLA-B*5701 positive.
4. Hypersensitivity to any component of the study drugs.
5. Pregnant or breastfeeding females.
6. Enrolled in one or more investigational drug protocols within 30 days of Screening.
7. An active Center for Disease Control and Prevention (CDC) Category C disease, except cutaneous Kaposi's sarcoma not requiring systemic therapy during the trial (see Appendix 1).

8. Ongoing clinically relevant hepatitis at Screening and/or positive for Hepatitis B (+HbsAg).
9. Creatinine clearance <50 mL/min via the Cockroft-Gault method [Cockroft, 1976]. A single repeat is allowed to determine eligibility.
10. Verified Grade 4 laboratory abnormality at Screening unless the Investigator can provide a compelling explanation (e.g. elevated Creatine Phosphokinase (CPK) due to exercise) for the laboratory result(s) and has the assent of the Sponsor. A single repeat is allowed to determine eligibility.
11. Any other laboratory abnormality or medical condition at Screening, which, in the opinion of the investigator, would preclude the subject's participation in the study (e.g. elevated liver function tests (LFTs) or pancreatitis, etc).
12. Immunization within 30 days prior to first dose of Investigational Product.
13. Any exposure to treatment with immunomodulating agents (such as systemic corticosteroids, interleukins, or interferons) or receipt of an HIV-1 immunotherapeutic vaccine within 90 days prior to Screening. Subjects using inhaled corticosteroids or short-course systemic corticosteroids (≤14 days) are eligible for enrollment.
14. Treatment with radiation therapy or cytotoxic chemotherapeutic agents within 90 days prior to Screening, or an anticipated need for these agents within the study period.
15. Treatment within 30 days prior to first dose of Investigational Product for or an anticipated need during the study of any medications which can have interactions with the study medications, TDF, FTC, ABC, 3TC, ATV and/or RTV, as described in current product labelling (e.g. use of proton pump inhibitors with ATV).
16. Any previous abacavir-containing regimen

Specific information regarding warnings, precautions, contraindications, adverse events, and other pertinent information on the Investigational Product that may impact subject eligibility is provided in each specific product label.

4.4. Subject Withdrawal

4.4.1. Subject Withdrawal from Study

A subject must be withdrawn from the study when:

- A subject becomes Hepatitis B positive (+ HbsAg).
- Subject requires the use of any prohibited study medication, unless explicit approval is given by the Sponsor in consultation with the Investigator (see Prohibited Medications, Section 5.7.2).

- A subject is significantly non-compliant with the requirements of the protocol (based upon the discretion of the investigator).
- A subject becomes pregnant (see Pregnancy, Section 6.4.8).
- A subject has an adverse experience that would, in the investigator's judgment, make continued participation in the study an unacceptable risk.
- Subject experiences a toxicity that meets the criteria for withdrawal (see Toxicity Management, Section 6.4.4)
- Subjects who become prisoners or become involuntarily incarcerated for treatment of either a psychiatric or physical (e.g., infectious disease) illness.
- Subject has a plasma HIV-1 RNA ≥2000 c/mL at the confirmatory virologic failure timepoint.
- Subject has two consecutive HIV-1 RNA measurements ≥2000 c/mL at any time.
- The Sponsor discontinues the study.

A subject may voluntarily discontinue participation in this study at any time. The investigator may also, at his or her discretion, withdraw the subject from participating in this study at any time.

4.4.2. Management of Withdrawn Subjects

If a subject is withdrawn from the study for any reason, the investigator must make every effort to perform the evaluations noted in the Time and Events table (see Section 6.1).

All data from the withdrawal visit should be recorded, as they comprise an essential evaluation that should be done prior to discharging any subject from the study.

If a subject is withdrawn from the study due to an AE (See Section 6.4.5.1) or serious adverse event (SAE) (See Section 6.4.5.2), the procedures stated in Section 6.4.5 (AEs and SAEs) must be followed and the AE must be followed-up until resolution.

Withdrawn subjects will not be replaced.

5. STUDY TREATMENTS

5.1. Investigational Product

The contents of the label will be in accordance with all applicable regulatory requirements.

Under normal conditions of handling and administration, Investigational Product is not expected to pose significant safety risks to site staff. A Material Safety Data Sheet (MSDS) describing the occupational hazards and recommended handling precautions will be provided to site staff if required by local laws or will otherwise be available from the Sponsor upon request.

Investigational Product must be stored in a secure area under the appropriate physical conditions for the product. Access to and administration of the Investigational Product will be limited to the investigator and authorized site staff. Investigational Product must be dispensed or administered only to subjects enrolled in the study and in accordance with the protocol.

- TDF/FTC will be provided as a fixed dose combination tablet (Truvada) which contains 300 mg of TDF (as tenofovir disoproxil fumarate) and 200 mg of FTC. The tablets are film-coated, blue capsule-shaped and debossed with “GILEAD” on one side and with “701” on the other side. TDF/FTC is packaged in bottles of 30 with child-resistant closures.
- ABC/3TC will be provided as a fixed dose combination tablet (EPZICOM®), which contains 600 mg of ABC (as abacavir sulfate) and 300 mg of 3TC. The tablets are film-coated, orange with a modified-capsule-shape and imprinted with GS FC2 and the other side plain. ABC/3TC is packaged in bottles of 30 with child-resistant closures.
- ATV (Reyataz) will be provided as commercially available 200 mg and 300 mg oral capsules. The 200 mg capsules are blue, gelatin capsules imprinted with BMS 200 mg and 3631. The 300 mg capsules are red and blue, gelatin capsules imprinted with BMS 300 mg and 3622. Reyataz 200 mg and 300 mg capsules are packaged in child-resistant bottles of 60 and 30 capsules, respectively.
- RTV (Norvir) will be provided as commercially available 100 mg oral capsules. The 100 mg capsules are white, soft-gelatin capsules imprinted with the Abbott corporate logo and Abbo-Code DS. Norvir capsules are packaged in bottles of 30 capsules each. Note: Norvir tablets may be substituted for Norvir capsules pending commercial availability. In this case, Norvir tablets should be administered and stored as per product information.

Investigational Product	Dose and Dose Interval	Total Daily Dose
**Treatment Arm A (Simplification)**		
EPZICOM<br>(ABC/3TC)	1 X 600 mg/300 mg tablet once-daily	600 mg/300 mg
Reyataz (atazanavir)	2 X 200 mg capsules once-daily with food	400 mg
**Treatment Arm B (Continuation)**		
Truvada<br>(TDF/FTC)	1 X 300 mg/200 mg tablet once-daily	300 mg/200 mg
Reyataz (atazanavir)	1 X 300 mg capsule once-daily with food	300 mg
Norvir (ritonavir)	1 X 100 mg capsule once-daily with food	100 mg

All Investigational Products will be administered orally. It is required that subjects take atazanavir with a meal.

All Investigational Products should be stored as follows until the time of dispensing:

**Truvada** tablets (TDF/FTC)	Store at 25°C (77°F)[1]
**EPZICOM** tablets (ABC/3TC)	Store up to 25°C (77°F)[1]
**Reyataz** capsules (atazanavir 200 mg/300 mg)	Store up to 25°C (77°F)[1]
**Norvir** capsules (ritonavir 100 mg)	Store 2-8°C (36-46°F)[2]

1. Permitted excursions up to 15-30°C (59-86°F)
2. Store in the refrigerator between 2°-8°C (36°-46°F) until dispensed. Refrigeration of Norvir soft gelatin capsules by the subject is recommended, but not required if used within 30 days and stored below 25°C (77°F)

5.2. Permitted Alternative Regimens

The Sponsor will provide ATV, RTV, ABC/3TC, TDF/FTC, ZDV/3TC or FPV in the event an alternative regimen is required per protocol. For renal toxicity, reimbursement will be offered for appropriate treatment (e.g. non fixed-dose tablets). All antiretroviral agents in the alternative regimen should be administered and stored in accordance with the current approved product labeling.

5.3. Treatment Assignment

Subjects will be assigned to study treatment in accordance with the randomization schedule. Subjects will be stratified by initial antiretroviral regimen received (ATV/RTV + TDF/FTC as initial regimen OR as first or second switch regimen) and randomized 2:1 to receive one of the following anti-retroviral therapy (ART)-regimens below for 48 weeks. Subjects will have twice the chance to be randomized to the simplification arm than the continuation arm.

**Treatment Arm A: (Simplification)**	ATV 400 mg once daily + ABC/3TC 600 mg/300 mg once daily
**Treatment Arm B: (Continuation)**	ATV/RTV 300 mg/100 mg once daily + TDF/FTC 300 mg/200 mg once daily

If a subject is eligible for randomization, the investigator (or designee) will call RAMOS (Registration and Medication Ordering System) and the subject will be assigned a randomization number. The randomization code is on file with the Sponsor and with RAMOS.

Training on the use of RAMOS and detailed user worksheets will be provided by the Sponsor prior to study start.

Randomization numbers are unique and may not be reassigned to another study subject.

5.4. Blinding

There will be no blinding. This is an open-label study.

5.5. Product Accountability

In accordance with local regulatory requirements, the investigator, designated site staff, or head of the medical institution (where applicable) must document the amount of Investigational Product dispensed and/or administered to study subjects, the amount returned by study subjects, and the amount received from and returned to the Sponsor, when applicable. Product accountability records must be maintained throughout the course of the study.

The Principal Investigator or authorized designee must sign for receipt and final disposition of all Investigational Products. To track Investigational Product receipt and return at the site level, signed and dated shipping and return forms must be maintained at the site. All Investigational Products will be handled and stored in accordance with the product label or information provided by the sponsor.

Within each subject's electronic case report form (eCRF), the name and total daily dose of each Investigational Product will be recorded. Start and stop dates of each Investigational Product must be recorded on this page. Date of resumption of original protocol dose and the date/reason for any permanent Investigational Product discontinuation will also be recorded.

Separate from the eCRF, sites will maintain a separate Investigational Product Accountability Log for each study drug to include the following:

- Subject number
- Quantity of Investigational Product dispensed/returned (unit of accountability=pill count)
- Lot or inventory number
- Dispensing/return date
- Signature or initials of individual dispensing or receiving the Investigational Product
- Comment area for noting discrepancies.

5.6. Treatment Compliance

At each study visit (exclusive of Week 2, Week 48, and Withdrawal visits), the site pharmacist or staff should dispense sufficient supplies of each Investigational Product to provide adequate supply for each subject until the next scheduled visit. Beginning with the Week 4 study visit, the pharmacist or site staff should account for all Investigational Product (including bottle) returned. Subjects must be educated to return all Investigational Product and bottles to the site at each study visit. This includes bottles that are empty, partially full, and completely full.

At the end of the study and at appropriate intervals during the study, all unused Investigational Product must be returned to the Sponsor according to procedures dictated by the study monitor or destroyed at the site and adequately documented. Monitors will regularly access pharmacy/dispensing records. Cases of suspected negligence will be investigated.

5.7. Concomitant Medications and Non-Drug Therapies

5.7.1. Permitted Medications and Non-Drug Therapies

Concomitant medications should be administered only as medically necessary during the study. Chemoprophylaxis for HIV-associated conditions is encouraged, if appropriate, at the discretion of the subject and his/her physician. All concomitant medications, blood products, and vaccines taken during the study will be recorded in the eCRF with dates of administration.

Hematological supportive therapy with Granulocyte Colony-Stimulating Factor (G-CSF) or erythropoietin will be permitted. Subjects being treated with G-CSF or erythropoietin at the time of Screening or Baseline will be allowed to participate in the study.

Because non-HIV vaccines may cause a temporary increase in the level of HIV-1 plasma RNA, it is recommended that a vaccine, if necessary, be given during or immediately after a scheduled visit after all laboratory tests have been drawn. This approach will minimize the risk of non-specific increases in the level of HIV-1 plasma RNA at the next scheduled assessment.

Subjects with confirmed virologic failure who are eligible to switch to an alternative antiretroviral regimen should administer medically necessary concomitant medications or non-drug therapies in accordance with recommendations from the current approved antiretroviral product label.

5.7.2. Prohibited Medications and Non-Drug Therapies

HIV immunotherapeutic vaccines are not permitted at any time during the study.

Other experimental agents, antiretroviral drugs, immunomodulators, cytotoxic chemotherapy, or radiation therapy may not be administered.

Refer to current prescribing information regarding medications that are prohibited for use with Investigational Products and substituted products contained in this study.

5.8. Treatment after the End of the Study

The Sponsor will not provide treatment after completion of the study.

5.9. Treatment of Investigational Product Overdose

An overdose is any dose greater than those described below for each study medication. For the purposes of this study, an overdose is not an adverse event (AE, Section 6.4.5.1 unless it is accompanied by a clinical manifestation associated with the overdose. If the clinical manifestation presents with serious criteria, the event is a serious adverse event (SAE, Section 6.4.5.2).

If an overdose occurs and is associated with an adverse event requiring action, all study medications should be temporarily discontinued until the adverse event resolves.

Investigational Product	Overdose
Truvada (TDF/FTC)	≥2 tablets per dose
EPZICOM (ABC/3TC)	2 tablets per dose
Atazanavir	>1200 mg/dose
Ritonavir	>600 mg/dose
**HSR Substitution Medication**	**Overdose**
COMBIVIR® (ZDV/3TC)	9 tablets per dose

The investigator should use clinical judgement in treating overdose, as the Sponsor is unable to recommend specific treatment.

6. STUDY ASSESSMENTS AND PROCEDURES

Written informed consent must be obtained from each potentially eligible subject (or his/her legal representative) by study site personnel **prior** to the initiation of any Screening procedures as outlined in this protocol. The consent form must have been approved by the Institutional Review Board / Independent Ethics Committee (IRB/IEC). After signing an informed consent, subjects will complete Screening assessments to determine subject eligibility.

Each subject being screened for study enrollment evaluation will be assigned a subject number. This number will be given sequentially in chronological order of subject presentation according to a numeric roster provided by the Sponsor.

Subjects who qualify must return no more than 35 days from the day of the Screening visit to begin study treatment. Subjects not meeting all inclusion and exclusion criteria at initial screen may be re-screened once at the discretion of the investigator after consultation with the Sponsor**.** A subject, who is randomized into the trial and subsequently withdraws from the study for any reason, may not be re-screened.

A single repeat test per analyte is allowed during the Screening period. **However, a repeat plasma HIV-1 RNA measurement is not allowed if the initial Screening value is >75 c/mL.**

6.1. Time and Events Schedule

Table 1 Time and Events Table

Procedures/Clinical Evaluation	Screen	Baseline	Treatment Week						Early W/D[a]	F/U[b]	Confirmation of VF[e,i]
			2	4	12	24	36	48			
**Study Assessments**											
Written Informed Consent (including PGx)	x										
Inclusion/Exclusion Criteria	x	x									
Subject Demography	x	x									
Medical History	x	x									
HIV-1 Associated Conditions		x	x	x	x	x	x	x	x		
CDC Classification	x	x	x	x	x	x	x	x	x		
HIV risk factors/mode of transmission		x									
Historical nadir CD4 value[k]		X[k]									
Limited Physical Exam (Body Weight, Height)		x									
Dispense Investigational Product		x		x	x	x	x				
**Safety Assessments**											
Vital Signs (BP, HR)		x				x		x	x		
Concomitant Medication	x	x	x	x	x	x	x	x	x		x
Smoking History[c]		x[c]				x[c]		x[c]	x[c]		
Adverse Events	x[d]	x[d]	x	x	x	x	x	x	x	x	x
**Laboratory Assessments**											
Quantitative Plasma HIV-1 RNA	x[e]	x[e]	x[e]	x[e]	x[e]	x[e]	x[e]	x[e]	x[e]	x (if needed)	x
CD4+/CD8+ Lymphocyte Subsets	x	x		x	x	x	x	x	x	x (if needed)	x
Hematology	x	x	x	x	x	x	x	x	x	x (if needed)	
Chemistry	x	x	x	x	x	x	x	x	x	x (if needed)	
Fasting lipids and Fasting Glucose[f]		x		x		x		x	x		

Procedures/Clinical Evaluation	Screen	Baseline	Treatment Week						Early W/D[a]	F/U[b]	Confirmation of VF[e,i]
			2	4	12	24	36	48			
CrCl (via C-G) and GFR (via MDRD)	x	x	x	x	x	x	x	x	x	X (if needed)	
HLA-B*5701 determination	x										
Hepatitis B antibody (HBsAg)	x										
Hep C (Anti- HCV Ab)		x									
Pregnancy Test[g]	x	x						X	X		
Urine Chemistry Analytes		x				x		x	x		
PGx Sampling[h]		x									
Plasma for storage (genotypic and phenotypic analyses)		x	x	x	x	x	x	x	x		x
**Exploratory Assessments**											
Blood CV Biomarkers		x				x		x	x		
Blood Bone Biomarkers		x				x		x	x		
Urine Biomarkers		x				x		x	x		
Serum Parathyroid hormone		x				x		x	x		
CogState Neurocognitive Battery[j]		x				x		x	x		

a. W/D – Study withdrawal. Withdrawal evaluations performed if subject discontinues prematurely from the study.
b. F/U – Study follow-up. Subjects will be contacted approximately 2-4 weeks after Week 48 or W/D by telephone for follow-up visit. If resolution of on-going AE(s) or confirmation of virologic failure is required the subject will return to the clinic for follow-up visit.
c. Smoking history will be used to calculate Framingham cardiovascular risk score which includes assessment of age, gender, total cholesterol, HDL, SBP, BP lowering medication use and smoking use.
d. Only SAEs related to study participation will be collected between obtaining written informed consent and administration of Investigational Products on Day 1.
e. A confirmatory HIV-1 RNA should be scheduled at least 28 days after a plasma HIV-1 RNA ≥400 c/mL result for confirmation of virologic failure.
f. Fasting is required (no food in previous 6-8 hours) except for the early W/D visit.
g. Required for females of childbearing potential only. Serum pregnancy test to be performed at Screening, Week 48 and Early Withdrawl; Urine pregnancy test to be performed at Baseline. Pregnancy test may be performed at the discretion of the investigator if pregnancy is suspected at any time during the trial.
h. PGx sample collection may be performed at any time starting at Baseline although it should be performed at the earliest time point possible.
i. Confirmation of virologic failure. Subjects should be asked about potential compliance issues, illness or recent immunizations. It is recommended that subjects be adherent to study medications for at least 28 days before returning for a confirmatory HIV-1 RNA.
j. A pre-baseline neurocognitive test is required to ensure that the subject is familiar with the testing modality. This test will be performed on the same day as the baseline test.
k. This value can be recorded at any time during the study using the subject's medical history

6.2. Efficacy

6.2.1. Primary Endpoint

- Proportion of subjects who maintain plasma HIV-1 RNA <50 c/mL at 24 weeks by time to loss of virologic response (TLOVR) algorithm

6.2.2. Secondary Efficacy Endpoints

- Proportion of subjects with plasma HIV-1 RNA <50 c/mL at 48 weeks
- Proportion of subjects with plasma HIV-1 RNA <400 c/mL at 24 and 48 weeks
- Change from Baseline in HIV-1 RNA and CD4+ cell count
- Time to virologic failure
- Identification of genotypic and phenotypic resistance in virus from subjects with virologic failure

6.2.3. Efficacy Evaluations

Efficacy assessments will consist of the following investigations:

- Plasma for quantitative HIV-1 RNA will be collected at Baseline and Weeks 2, 4, 12, 24, 36, 48, withdrawal (W/D) and, if necessary, follow-up visit.
- Blood samples for CD4+ and CD8+ lymphocyte counts will be collected at Baseline and Weeks 4, 12, 24, 36, 48, W/D and, if necessary, follow-up visit.
- HIV-associated conditions will be recorded at Baseline and Weeks 2, 4, 12, 24, 36, 48 and W/D.
- Death and clinical disease progression. CDC Classification will be recorded at Baseline and Weeks 2, 4, 12, 24, 36, 48 and W/D.

  Indicators of disease progression are defined as:

  - Baseline CDC Category A → CDC Category B Event or death
  - Baseline CDC Category A → CDC Category C Event or death
  - Baseline CDC Category B → CDC Category C Event or death
  - Baseline CDC Category C → New CDC Category C Event or death.

6.3. Safety

6.3.1. Safety Endpoints

- Change in fasting lipid profiles (total cholesterol, LDL, HDL, triglycerides) compared to Baseline
- Grade 2 to 4 AEs and all SAEs

6.3.2. Safety Evaluations

Safety assessments will consist of the following investigations:

- Clinical evaluations of adverse events will be made at Baseline and Weeks 2, 4, 12, 24, 36, 48, W/D, when confirming virologic failure and at follow-up.
- Vital signs (BP and HR) will be assessed at Baseline and Weeks 24, 48 and W/D.
- Serum chemistry and hematology will be performed at Baseline and Weeks 2, 4, 12, 24, 36, 48 and W/D. Evaluations may occur at the post-treatment follow-up if necessary for resolution of an ongoing laboratory adverse event.
- Fasting lipid and fasting glucose evaluations will be made at Baseline and Weeks 4, 24, 48, and W/D.
- Creatinine clearance (via Cockroft-Gault) and Modification of Diet in Renal Disease (MDRD) GFR will be calculated at Screening, Baseline and Weeks 2, 4, 12, 24, 36, 48, and W/D.
- HLA-B*5701 and Hepatitis C status will be assessed at Screening only.
- Concomitant medications will be recorded at Baseline and Weeks 2, 4, 12, 24, 36, 48, W/D and when confirming virologic failure.
- Framingham cardiovascular risk assessment will be performed at Baseline and Weeks 24, 48 and W/D.

6.3.2.1. Clinical Laboratory Assessments

Hematology, clinical chemistry, urine chemistry analytes and additional parameters to be tested as per the time and events table (See Section 6.1):

Hematology

Platelet Count	*RBC Indices*:	*WBC Differential:*
RBC Count	MCV	Neutrophils
WBC Count (absolute)		Lymphocytes
Hemoglobin		Monocytes
Hematocrit		Eosinophils
		Basophils

Clinical Chemistry

BUN	Potassium	AST (SGOT)	Total and direct bilirubin
Creatinine	Chloride	ALT (SGPT)	Lipase
Glucose, fasting	Bicarbonate	Alkaline phosphatase	Albumin
	Calcium	Creatine phosphokinase	Total Protein
	Magnesium	Phosphorus, inorganic	
	Sodium		

Fasting Lipid Profile

Triglyceride	High density lipoprotein (HDL)
Total Cholesterol	Low density lipoprotein (LDL)

Urine Chemistry Analytes

Glucose (quantified), total protein (quantified), phosphorus, creatinine (quantified) [collected as random samples]

Other Tests

Hepatitis B surface antigen (HBsAg) and Hep C (Anti-HCV Ab)
Serum or urine human chorionic gonadotrophin (hCG) pregnancy test for females only
Estimated Creatinine Clearance by Cockroft-Gault calculation<br>Modification of Diet in Renal Disease (MDRD) GFR calculation
HLA-B*5701
Suspected Abacavir HSR: Hematology (hemoglobin, RBC, MCV, Platelet, WBC with diff), Chemistry (CPK, Creatinine, T. bilirubin, ALT, AST, GGT), Lipase, Abs CD4, HIV RNA, HsCRP

6.4. Exploratory

6.4.1. Exploratory Endpoints

- Change from Baseline in neurocognition scores measured by CogState (see Appendix 2 CogState).
- Changes from Baseline in various cardiovascular, bone, and renal biomarkers, which may or may not include markers such as high sensitivity C-reactive protein (hsCRP), interleukin 6 (IL-6), D-dimer, serum procollagen type 1 N-propeptide (P1NP), serum bone specific alkaline phosphatase (BSAP), serum parathyroid hormone (PTH), serum c-telopeptide (CTx), osteocalcin, vitamin D 1,25-OH, and beta-2 microglobulin

6.4.2. Exploratory Evaluations

Exploratory assessments will consist of the following investigations:

- The pre-Baseline nadir CD4+ cell count will be collected. This may be collected at any time during the study.
- Neurocognitive assessment via computerized questionnaire will be assessed at Baseline and Weeks 24, 48 and W/D.
    - A pre-baseline neurocognitive assessment is required to ensure that the subject is familiar with the testing modality. The pre-baseline assessment will be performed on the same day as the baseline assessment. The pre-baseline assessment data will not be collected.

- Blood or urine samples for biomarker assessments will be collected at Baseline and Weeks 24, 48 and W/D.

6.4.3. General Toxicity Management

Adverse events that occur during the trial should be evaluated by the investigator and graded according to the modified Division of AIDS toxicity scales (see Appendix 3: DAIDS Division of Aids Table for Grading the Severity of Adult and Pediatric Adverse Events for laboratory test abnormalities and clinical toxicity).

The adverse event profiles for the combinations used in this study have not yet been fully defined. Trial medication may be interrupted at the discretion of the investigator and according to the severity of the adverse event. No dose reductions of any study medication will be allowed with the exception of declining creatinine clearance as indicated in product information.

Decisions regarding sequential reintroduction of study drugs or temporary interruption of one or more but not all drugs within the ART regimen should be made with the understanding that these changes may result in incomplete viral suppression and selection of resistant virus. Guidance is provided below on study medication interruptions based on the severity of the study medications-related adverse event. All changes in the Investigational Product regimen must be accurately recorded in the subject's eCRF.

6.4.3.1. Grade 1 or Grade 2 Toxicity/Adverse Event

Subjects who develop a Grade 1 or Grade 2 AE or toxicity may continue IP at the discretion of the investigator. (NOTE – please refer to Specific Toxicities for exceptions to this guideline in Section 6.4.4). Subjects who choose to withdraw from study due to a Grade 1 or 2 AE should have study withdrawal and follow-up evaluations completed.

6.4.3.2. Grade 3 Toxicity/Adverse Event

Subjects who develop a Grade 3 AE or toxicity should be managed as follows:

1. If the investigator has compelling evidence that the Grade 3 AE or toxicity has not been caused by IP, dosing may continue, after discussion with the Sponsor.
2. Subjects who develop a Grade 3 AE or toxicity, which the investigator considers related or possibly related to IP, should have all IP withheld and be rechecked each week until the adverse event returns to Grade 2. Once the AE is ≤ Grade 2, IP may be re-started at full dose at the discretion of the investigator. (Please refer to Section 6.4.4.5 for exception with hyperbilirubinemia and hypertriglyceridemia).

   NOTE: In the event of a discontinuation of ABC/3TC for any reason, re-initiation of this drug should be undertaken with caution. Health care providers should obtain a complete history of the events surrounding the discontinuation of ABC/3TC. If there are symptoms consistent with a hypersensitivity reaction, ABC should not be reinitiated, regardless of a subject's HLA-B*5701 status. If there is no evidence of a prior reaction, the subject may restart treatment with ABC/3TC. The subject and

health care provider should be aware of the possibility of a rapid-onset hypersensitivity reaction upon re-initiation of ABC, which may be life-threatening, and the subject should be able to, if necessary, receive prompt medical evaluation (refer to Section 6.4.4.1).

3. Should the same Grade 3 AE recur within 28 days in the same subject, IP must be permanently discontinued and the subject withdrawn from study if the investigator considers the AE related to IP. If the AE recurs after 28 days, the above management scheme should be followed. Subjects experiencing Grade 3 AEs requiring permanent discontinuation of IP should be followed weekly until resolution of the AE and encouraged to have withdrawal study evaluations completed. A follow-up visit should be performed 2-4 weeks after the last dose of IP.

Subjects with Grade 3 asymptomatic laboratory abnormalities should be investigated for all potential non-drug related causes, and may continue therapy if the investigator has compelling evidence that the toxicity is not related to IP, following discussion with the Sponsor.

6.4.3.3. Grade 4 Toxicity/Adverse Event

Subjects who develop a Grade 4 AE or toxicity should have all IP permanently discontinued. However, if the investigator has compelling evidence that the AE is not causally related to the Investigational Product (s), dosing may continue after discussion with and assent from the Sponsor. Subjects experiencing Grade 4 AEs requiring permanent discontinuation of IP should be followed weekly until resolution of the AE and encouraged to complete the withdrawal study evaluation. A follow-up visit should be performed 2-4 weeks after the last dose of IP. (Please refer to Section 6.4.4.5 for exception with hyperbilirubinemia).

Subjects with Grade 4 asymptomatic laboratory abnormalities should be investigated for all potential non-drug related causes, and may continue therapy if the investigator has compelling evidence that the toxicity is not related to IP.

A follow-up visit should be performed 2-4 weeks after the last dose of IP if laboratory abnormalities are ongoing at the time of treatment completion.

6.4.4. Specific Toxicities/Adverse Event Management

6.4.4.1. Abacavir Hypersensitivity Reaction

All hypersensitivity reactions to abacavir will be considered and reported as SAEs regardless of severity (refer to Section 6.4.5.2).

In clinical studies EPZ108859 (ARIES), CNA109586 (ASSERT) and CNA106030 (PREDICT-1), 0.8% (4/515), 3.1% (6/192) and 3.4 % (27/803) of subjects with a negative HLA-B*5701 status receiving abacavir developed a hypersensitivity reaction, respectively. In rare cases, hypersensitivity reactions to abacavir have proven fatal.

6.4.4.1.1. Risk Factors for Abacavir Hypersensitivity Reaction

Studies have shown that carriage of the HLA-B*5701 allele is associated with a significantly increased risk of a hypersensitivity reaction to ABC. In the prospective study CNA106030 (PREDICT-1), the use of such pre-therapy Screening for the HLA-B*5701 allele and subsequently avoiding ABC in HLA-B*5701 positive patients, reduced the incidence of clinically suspected ABC HSR from 7.8% (66 of 847) to 3.4% (27 of 803) (p<0.0001). Based on this study, it is estimated that 48% to 61% of patients with the HLA-B*5701 allele will develop a hypersensitivity reaction during the course of abacavir treatment compared with 0% to 4% of patients who do not have the HLA-B*5701 allele [Mallal, 2008]. While the PREDICT-1 study population was predominantly white, the association between HLA-B*5701 and ABC HSR appears to be generalisable across racial groups [Saag, 2008; Sun, 2007].

In any patient treated with ABC, the clinical diagnosis of suspected HSR must remain the basis of clinical decision making. Even in the absence of the HLA-B*5701 allele, it is important to permanently discontinue ABC and not rechallenge with ABC (i.e., ZIAGEN®, TRIZIVIR, EPZICOM® or KIVEXA®) if a HSR cannot be ruled out on clinical grounds, due to the potential for a severe or even fatal reaction.

6.4.4.1.2. Clinical Description of the Hypersensitivity Reaction

The abacavir hypersensitivity reaction is characterised by the appearance of symptoms indicating multi-organ involvement. The majority of patients have fever and/or rash as part of the syndrome; however reactions have occurred without rash or fever.

Symptoms can occur at any time during treatment with abacavir, but usually appear within the first six weeks of initiation of treatment (median time to onset 11 days). The symptoms worsen with continued therapy and can be life threatening. These symptoms usually resolve shortly after discontinuation of abacavir.

Frequently observed signs and symptoms include fever, rash, malaise or fatigue, gastrointestinal symptoms such as nausea, vomiting, diarrhea, or abdominal pain and respiratory symptoms such as dyspnea, sore throat, or cough. Other signs and symptoms include myalgia, arthralgia, oedema, pharyngitis, headache, paresthesia or myolysis.

Physical findings may include rash (usually maculopapular or urticarial), lymphadenopathy or mucous membrane lesions (conjunctivitis, mouth ulceration). Abnormal chest x-ray findings may also be present (predominantly infiltrates, which can be localised).

Laboratory abnormalities may include elevated liver function tests (such as hepatic transaminases), increased creatine phosphokinase or creatinine levels, and lymphopenia.

Anaphylaxis, hypotension, liver failure, renal failure, adult respiratory distress syndrome or respiratory failure may occur.

Some patients with hypersensitivity were initially thought to have respiratory disease (pneumonia, bronchitis, pharyngitis), a flu-like illness, gastroenteritis or reactions to other medications. This delay in diagnosis of hypersensitivity has resulted in abacavir being continued or re-introduced, leading to a more severe hypersensitivity reaction or death. Therefore, the diagnosis of hypersensitivity reaction should be carefully considered for patients presenting with symptoms of these diseases.

Restarting any abacavir-containing product following hypersensitivity reaction results in a prompt return of symptoms within hours. **This recurrence of the hypersensitivity reaction may be more severe than on initial presentation, and may include life-threatening hypotension and death.**

6.4.4.1.3. Clinical Management of Hypersensitivity Reactions

Patients developing signs or symptoms of hypersensitivity MUST contact their doctor immediately for advice.

If a hypersensitivity reaction is diagnosed, the abacavir-containing product MUST be discontinued immediately, regardless of a subject's HLA-B*5701 status. The patient should be asked to return all unused supplies of the abacavir-containing product for disposal to prevent an accidental re-challenge.

An abacavir containing medicinal product (ZIAGEN , TRIZIVIR , EPZICOM® or KIVEXA), MUST NEVER be administered following a hypersensitivity reaction, as more severe symptoms will recur within hours and may include life-threatening hypotension and death.

To avoid a delay in diagnosis and minimize the risk of a life-threatening hypersensitivity reaction, the abacavir-containing product should be permanently discontinued if hypersensitivity cannot be ruled out, **even when other diagnoses are possible (respiratory diseases, flu-like illness, gastroenteritis or reactions to other medications) and regardless of a subject's HLA-B*5701 status.**

Symptomatic support for abacavir hypersensitivity may be indicated. This should include, for example, administration of intravenous fluids to patients who develop hypotension. Antihistamines or corticosteroids have been used in cases of abacavir hypersensitivity, however there are no clinical data demonstrating the benefit of these in the management of the reaction.

Laboratory and other investigations which may be useful in the evaluation and treatment of abacavir hypersensitivity include, but may not be limited to, measurement of ALT, AST, creatine phosphokinase, serum creatinine and white blood cell differential count and chest x-ray, if respiratory symptoms are present.

6.4.4.1.4. Special considerations following an interruption of abacavir therapy

If therapy with abacavir has been discontinued and restarting therapy is under consideration, the reason for discontinuation should be evaluated to ensure that the patient did not have symptoms of a hypersensitivity reaction, regardless of a subject's HLA-B*5701 status. **If hypersensitivity reaction cannot be ruled out, no medicinal product containing abacavir (ZIAGEN , TRIZIVIR , EPZICOM® or KIVEXA) should be restarted.**

There have been infrequent reports of hypersensitivity reaction following reintroduction of an abacavir-containing product where the interruption was preceded by a single key symptom of hypersensitivity (rash, fever, malaise/fatigue, gastrointestinal symptoms or a respiratory symptom). If a decision is made to restart any abacavir-containing product in these patients, this should be done only under direct medical supervision.

On very rare occasions hypersensitivity reactions have been reported in patients who have re-started therapy, and who had no preceding symptoms of a hypersensitivity reaction. If a decision is made to re-start an abacavir-containing product, this must be done only if medical care can be accessed readily by the patient or others.

6.4.4.1.5. Essential patient information

With reference to this protocol section and the 'Subject Information and Consent Form' investigators must ensure that patients are fully informed regarding the following information on the hypersensitivity reaction:

- Patients must be made aware of the possibility of a hypersensitivity reaction to abacavir that may result in a life threatening reaction or death.
- Patients must be made aware that not all people who experience ABC HSR will be HLA-B*5701 positive. Therefore, patients who are HLA-B*5701 negative and who develop signs or symptoms consistent with a possible HSR to ABC **MUST STILL CONTACT their doctor IMMEDIATELY.**
- Patients who are hypersensitive to abacavir should be reminded that they must never take any abacavir containing medicinal product (**ZIAGEN , TRIZIVIR , EPZICOM® or KIVEXA**) again, regardless of their HLA-B*5701 status.
- In order to avoid restarting the abacavir-containing product, patients who have experienced a hypersensitivity reaction should be asked to return the remaining tablets or oral solution to the pharmacy.
- Patients who have stopped an abacavir-containing product for any reason, and particularly due to possible adverse reactions or illness, must be advised to contact their doctor before restarting.
- Each patient should be reminded to read the Package Leaflet included in the pack.
- Patients should be reminded of the importance of removing the Alert Card included in the pack, and keeping it with them at all times.

6.4.4.1.6. Reporting of Hypersensitivity Reactions

All cases of potential abacavir hypersensitivity should be reported as Serious Adverse Events (SAE) (see Section 6.4.5.2). In addition to reporting the case as an SAE, the site should immediately notify the Sponsor and within one week of the onset of the hypersensitivity reaction, complete the Abacavir Hypersensitivity Reaction (HSR) eCRF.

6.4.4.1.7. Therapy Management for Abacavir Hypersensitivity Reaction

Subjects who experience a suspected abacavir hypersensitivity reaction (ABC HSR) must discontinue abacavir and may chose from one of the following two options to remain on study:

- Substitute zidovudine/lamivudine (ZDV/3TC) for ABC/3TC. ZDV/3TC will be provided by the Sponsor.
- Substitute TDF/FTC for ABC/3TC and change back to original regimen of ATV/RTV + TDF/FTC. TDF/FTC and ATV/RTV will be provided by the Sponsor.

Abacavir substitution is only allowed for ABC hypersensitivity reactions. No NNRTI or PI substitutions are allowed. Any change **MUST** be discussed with **THE SPONSOR PRIOR TO THE CHANGE BEING MADE**.

6.4.4.2. Stevens Johnson Syndrome, Toxic Epidermal Necrolysis or Erythema Multiforme

Serious skin reactions such as Stevens Johnson Syndrome, Toxic Epidermal Necrolysis or Erythema Multiforme have been reported very rarely in patients taking abacavir-containing products. These patients generally do not have the cluster of additional symptoms (e.g., gastrointestinal and respiratory) that characterize the abacavir hypersensitivity reaction, but they do have features typical of these serious skin reactions.

If a serious skin reaction develops, the abacavir-containing product should be discontinued, and the patient should not be rechallenged with any abacavir-containing medicinal product (**ZIAGEN , TRIZIVIR , EPZICOM® or KIVEXA**).

As many products other than abacavir also cause these serious skin reactions, all other medicinal products that the patient is receiving should also be reviewed and discontinued as appropriate.

6.4.4.3. Rash Not Accompanied by Systemic Symptoms

Subjects receiving ABC who develop rash of any grade should be evaluated for the possibility of an HSR to abacavir or a serious skin reaction such as Stevens Johnson Syndrome, Toxic Epidermal Necrolysis or Erythema Multiforme and managed appropriately as outlined above. Rash may be caused by therapies in any of the major antiretroviral classes, or by other therapies commonly used as concurrent medications, such as cotrimoxazole. As it is not possible to provide an exhaustive list of products that

may cause rash in this protocol, please consult the product information leaflets for other products for information relating to rash.

The following guidance is provided for clinical management of subjects who experience rash alone in the absence of accompanying diagnosis of ABC hypersensitivity, systemic or allergic symptoms or signs of mucosal or target lesions. The toxicity ratings must be used to appropriately grade cutaneous events when recording adverse events.

Study treatment should be managed as outlined in the following table.

**Event**	**Action with Study Drug**	
	ATV + RTV	ATV
Grade 1 or 2 rash alone	Therapy can continue	Therapy can continue
Grade 1 or 2 rash with symptoms indicating multi-organ involvement[a]	Discontinue all Investigational Products and assess further. Therapy with ATV + RTV should not be reinitiated unless the investigator has compelling evidence that the rash is not caused by ATV + RTV	Discontinue all Investigational Products and assess further. Therapy with ATV should not be reinitiated unless the investigator has compelling evidence that the rash is not caused by ATV
Grade 3/4 rash alone or with symptoms indicating multi-organ involvement[a]	Discontinue all Investigational Products and assess further (see below).	Discontinue all Investigational Products and assess further (see below).

a Symptoms include: systemic symptoms (fever, GI symptoms including nausea, vomiting, diarrhea or abdominal pain) or allergic symptoms, or mucosal involvement, or severe tiredness, achiness or generally ill feeling.

Adjunctive treatment:

- For Grade 1 or 2 rash, antihistamines or topical corticosteroids may be prescribed. Systemic corticosteroids are not recommended for Grade 1 or 2 rash, as steroid therapy may mask the appearance of other symptoms leading to more serious systemic involvement.
- Subjects who develop a Grade 3 (vesiculation, or moist desquamation, or ulceration) or 4 rash (exfoliation, mucosal involvement, or target lesions [erythema multiform]) or any evidence of Stevens-Johnson Syndrome should have all study drugs discontinued and assessed appropriately. If Grade 3 or 4 rash is accompanied by systemic symptoms, ATV and/or RTV should be permanently discontinued. Steroids may be used for treatment of the event.

Subjects should be evaluated and followed aggressively for one week or until symptoms resolve or change, even though study medications have been discontinued.

Refer to local EPZICOM®, ATV and RTV prescribing information for complete information regarding management of rash.

The rash and any associated symptoms should be reported as adverse events (see Section 6.4.5.1) and appropriate toxicity ratings should be used to grade the events (See Appendix 2: DAIDS Toxicity Grading Scale).

If the aetiology of the rash can be definitively diagnosed as being due to a specific medical event or a concomitant medicinal product, routine management should be performed and documentation of the diagnosis provided.

6.4.4.4. Gastrointestinal Toxicity

Nausea and Vomiting

Although common, nausea following initiation of therapy with antiretroviral medications usually subsides or resolves during the first few weeks of treatment. Upper gastrointestinal adverse events may be reduced by taking medications with a meal, and by maintaining a steady oral intake, e.g., pretzels or other dry bread. Subjects who experience potentially treatment limiting nausea or vomiting should be treated with anti-emetics. No dose reduction or escalation of Investigational Products will be allowed.

Diarrhea

Subjects with ≥ Grade 1 diarrhea may be treated as needed with oral antidiarrheal agents.

6.4.4.5. Laboratory Toxicity

Hyperbilirubinemia

Any elevation in indirect bilirubin does not constitute a safety concern unless accompanied by >35% increase in direct bilirubin.

- For isolated ≥ Grade 3 unconjugated hyperbilirubinemia attributed to ATV, ATV should be continued unless associated with jaundice or scleral icterus that presents an intolerable cosmetic concern to the subject.
- For isolated ≥ Grade 3 unconjugated hyperbilirubinemia that cannot be attributed to ATV or a non-study drug-related cause, all study medications should be held pending evaluation of aetiology.
- A ≥ grade 2 elevation in total bilirubin (≥ 2 x ULN with >35% direct bilirubin) and ≥ grade 2 elevation in ALT (≥ 3 x ULN) is a SAE suggestive of significant liver toxicity which meets the criteria for Hy's Law and requires rapid evaluation and notification to the Sponsor within 24 hours. If this should occur, subject should immediately stop the Investigational Product(s), have their liver chemistries repeated, be closely monitored, and referred to a Hepatology specialist for further evaluation. Any subject meeting the above criteria should not be retreated or rechallenged with the Investigational Product(s).

Hypertriglyceridemia/Hypercholesterolemia

Please see the Recommendations of the Adult AIDS Clinical Trial Group Cardiovascular Disease Focus Group [Dubé, 2003] for full discussion of management of hyperlipidemia in the context of HIV therapy.

Anemia/Neutropenia

Transfusions and erythropoietin may be used to manage anemia. Neutropenia may be managed with G-CSF or GM-CSF, at the discretion of the investigator.

Lactic Acidosis/Severe Hepatomegaly with Steatosis

The relevance of asymptomatic lactic acid elevations is unclear, and lactates are not part of the routine safety evaluations or monitoring for this study. Should an investigator suspect the occurrence of lactic acidosis in any subject, **THE SPONSOR SHOULD BE CONSULTED**. Lactic acidosis and severe hepatomegaly with steatosis, including fatal cases, have been reported with the use of antiretroviral nucleoside analogues alone or in combination in the treatment of HIV infection. A majority of these cases have been in women. This syndrome is felt to be secondary to mitochondrial toxicity induced by the inhibitory effect of N(t)RTIs on DNA polymerase gamma, a key enzyme needed for mitochondrial DNA synthesis. Current knowledge regarding this syndrome is incomplete. Obesity and prolonged N(t)RTI exposure may be risk factors. Symptoms of lactic acidosis frequently involve non-specific symptoms such as fatigue, weakness, and fever, but in the majority of cases also involve symptoms suggestive of hepatic dysfunction such as nausea, vomiting, abdominal or epigastric discomfort, abdominal distension, hepatomegaly, and new onset elevated liver enzymes. Caution should be exercised when administering EPZICOM®, Truvada or COMBIVIR to any subject and particularly to those with known risk factors for liver disease. Treatment with EPZICOM®, Truvada or COMBIVIR should be suspended in any subject who develops clinical or laboratory findings suggestive of lactic acidosis or hepatotoxicity and **THE SPONSOR SHOULD BE CONSULTED**.

Pancreatitis

Cases of pancreatitis have occurred rarely in subjects treated with ABC, 3TC, TDF and ZDV. However it is not clear whether these cases were due to the medicinal products or to the underlying HIV disease. Treatment with **EPZICOM®, Truvada** or **COMBIVIR** should be stopped immediately if clinical signs, symptoms or laboratory abnormalities suggestive of pancreatitis occur.

Creatine Phosphokinase (CPK) Elevation

A Grade 3 or higher elevation in CPK should result in a repeat assessment within 2-4 weeks to ensure the result is transient or due to exercise and will not require a change in study treatment. A history regarding physical activity or exercise preceding the CPK evaluation should be obtained. Grade 4 elevations in CPK should have a repeat assessment after the subject has abstained from exercise for >24 hours. For persistent

Grade 4 CPK elevations that are considered possibly or probably related to the IP, IP should be discontinued and the subject withdrawn from the study.

6.4.4.6. Renal Toxicity

Nephrotoxicity

In addition to the Division of AIDS toxicity grading scale for serum creatinine, the National Kidney Foundation criteria for staging chronic kidney disease should be used to grade renal toxicity [National Kidney Foundation, 2002].

Stage	Description	GFR (mL/min/1.73m^2)
1	Kidney damage* with normal or increased GFR	≥90
2	Kidney damage* with mild reduction in GFR	60-89
3	Moderate reduction in GFR	30-59
4	Severe reduction in GFR	15-29
5	Kidney failure	<15 (or dialysis)

*Kidney damage is defined as pathologic abnormalities or markers of damage, including abnormalities in blood or urine tests or imaging studies.

Decline in Renal Function

The Cockroft-Gault equation for estimating creatinine clearance (CrCl) will be used to determine changes in dosing for both TDF/FTC and ABC/3TC in the event of declining renal function.

For subjects who experience progression to an estimated CrCl (calculated by Cockroft-Gault equation) to <50 mL/min judged by the investigator to be attributed to study medication, the offending agent(s) must be discontinued. No dose-reduction of the offending agent(s) will be allowed.

For subjects who experience progression to an estimated CrCl (calculated by Cockroft-Gault equation) to <50 mL/min to be NOT study drug-related, the investigator will choose from one of the following two management options for study medications:

1. Discontinue study medication(s) and switch to another agent in the same class and remain on study.
2. Dose reduce study medication (s) as indicated in prescribing information and remain on study.

*Note: The Sponsor will provide ATV, RTV, ABC/3TC, TDF/FTC, 3TC/ZDV or FPV for the purpose of constructing an alternative treatment regimen for decline in renal

function. Reimbursement will be offered for the cost associated with use of any agent not provided by the Sponsor.

Proximal Renal Tubule Dysfunction

Proximal Renal Tubule Dysfunction (PRTD) is defined as:

- Confirmed rise in serum creatinine of ≥0.5 mg/dL from Baseline AND serum phosphorus <2.0 mg/dL
- Either of the above accompanied by any two of the following:
  - Proteinuria (≥100 mg/dL)
  - Glycosuria (≥250 g/dL) in a non-diabetic
  - Low serum potassium (<3 mEq/L)
  - Low serum bicarbonate (<19 mEq/L)

Quantification of urine protein and urine glucose will not be routinely performed at each visit, therefore PRTD will be evaluated based upon investigators clinical judgment and assessment of initial chemistry findings to determine if further work-up is required for PRTD. If PRTD is suspected, the investigator should consult the Sponsor and conduct follow-up investigations per clinical practice.

Subjects meeting criteria for PRTD must return for a confirmatory assessment within 4 weeks. **If PRTD is confirmed, subject must discontinue study.**

PRTD is considered resolved when serum creatinine is ≤0.3 mg/dL above Baseline, serum phosphorus is ≥2.5 mg/dL, and serum bicarbonate is ≥19 mEq/L [Fisher, 2001].

6.4.5. Adverse Events

The investigator or site staff will be responsible for detecting, documenting and reporting events that meet the definition of an AE or SAE.

6.4.5.1. Definition of an AE

Any untoward medical occurrence in a patient or clinical investigation subject, temporally associated with the use of a medicinal product, whether or not considered related to the medicinal product.

Note: An AE can therefore be any unfavorable and unintended sign (including an abnormal laboratory finding), symptom, or disease (new or exacerbated) temporally associated with the use of a medicinal product. For marketed medicinal products, this also includes failure to produce expected benefits (i.e., lack of efficacy), abuse or misuse.

Events meeting the definition of an AE include:

- Exacerbation of a chronic or intermittent pre-existing condition including either an increase in frequency and/or intensity of the condition
- New conditions detected or diagnosed after Investigational Product administration even though it may have been present prior to the start of the study
- Signs, symptoms, or the clinical sequelae of a suspected interaction
- Signs, symptoms, or the clinical sequelae of a suspected overdose of either Investigational Product or a concomitant medication (overdose per se will not be reported as an AE/SAE).

“Lack of efficacy” or “failure of expected pharmacological action” per se will not be reported as an AE or SAE. However, the signs and symptoms and/or clinical sequelae resulting from lack of efficacy will be reported if they fulfill the definition of an AE or SAE.

Events that **do not** meet the definition of an AE include:

- Medical or surgical procedure (e.g., endoscopy, appendectomy); the condition that leads to the procedure is an AE
- Situations where an untoward medical occurrence did not occur (social and/or convenience admission to a hospital)
- Anticipated day-to-day fluctuations of pre-existing disease(s) or condition(s) present or detected at the start of the study that do not worsen
- The disease/disorder being studied, or expected progression, signs, or symptoms of the disease/disorder being studied, unless more severe than expected for the subject’s condition

6.4.5.2. Definition of a SAE

A serious adverse event is any untoward medical occurrence that, at any dose:

a. Results in death

b. Is life-threatening

   NOTE: The term 'life-threatening' in the definition of 'serious' refers to an event in which the subject was at risk of death at the time of the event. It does not refer to an event, which hypothetically might have caused death, if it were more severe.

c. Requires hospitalization or prolongation of existing hospitalization

   NOTE: In general, hospitalization signifies that the subject has been detained (usually involving at least an overnight stay) at the hospital or emergency ward for observation and/or treatment that would not have been appropriate in the physician’s office or out-patient setting. Complications that occur during hospitalization are AEs. If a complication prolongs hospitalization or fulfills any other serious criteria, the event is serious. When in doubt as to whether “hospitalization” occurred or was necessary, the AE should be considered serious.

Hospitalization for elective treatment of a pre-existing condition that did not worsen from Baseline is not considered an AE.

d. Results in disability/incapacity, or

NOTE: The term disability means a substantial disruption of a person's ability to conduct normal life functions. This definition is not intended to include experiences of relatively minor medical significance such as uncomplicated headache, nausea, vomiting, diarrhea, influenza, and accidental trauma (e.g. sprained ankle) which may interfere or prevent everyday life functions but do not constitute a substantial disruption.

e. Is a congenital anomaly/birth defect

f. Medical or scientific judgment should be exercised in deciding whether reporting is appropriate in other situations, such as important medical events that may not be immediately life-threatening or result in death or hospitalization but may jeopardize the subject or may require medical or surgical intervention to prevent one of the other outcomes listed in the above definition. These should also be considered serious. Examples of such events are invasive or malignant cancers, intensive treatment in an emergency room or at home for allergic bronchospasm, blood dyscrasias or convulsions that do not result in hospitalization, or development of drug dependency or drug abuse.

g. All events of possible drug-induced liver injury with hyperbilirubinemia defined as ALT ≥ 3xULN and bilirubin ≥ 2xULN **(>35% direct)** (or ALT ≥ 3xULN and INR>1.5, if INR measured) termed 'Hy's Law' events.

h. All clinically suspected cases of ABC HSR in subjects receiving an ABC-containing product

6.4.6. Laboratory and Other Safety Assessment Abnormalities Reported as AEs and SAEs

Any abnormal laboratory test results (hematology, clinical chemistry, or urinalysis) or other safety assessments (e.g., ECGs, radiological scans, vital signs measurements), including those that worsen from Baseline, and felt to be clinically significant in the medical and scientific judgement of the investigator are to be recorded as AEs or SAEs.

However, any clinically significant safety assessments that are associated with the underlying disease, unless judged by the investigator to be more severe than expected for the subject's condition, are **not** to be reported as AEs or SAEs.

6.4.7. Disease-Related Events and/or Disease-Related Outcomes Not Qualifying as SAEs

The events or outcomes listed in the CDC Classification System for HIV-1 Infections (Appendix 3: CDC Classification System for HIV-1 Infections (1993) will be recorded on the HIV-Associated Conditions electronic case report form (eCRF) page if they occur. However, these individual events or outcomes, as well as any sign, symptom, diagnosis, illness, and/or clinical laboratory abnormality that can be linked to any of these events or

outcomes are not reported to the Sponsor as AEs and SAEs even though such event or outcome may meet the definition of an AE or SAE, **unless the following conditions apply**:

- the Investigator determines that the event or outcome qualifies as an SAE under part 'f' of the SAE definition (see Section 6.4.5.2 "Definition of a SAE"), or
- the event or outcome is in the Investigator's opinion of greater intensity, frequency or duration than expected for the individual subject, or
- death occurring for any reason during a study, including death due to a disease-related event, will always be reported promptly.
- Lymphomas and invasive cervical carcinomas are excluded from this exemption; they must be reported as SAEs even if they are considered to be HIV related.

6.4.8. Pregnancy

6.4.8.1. Pregnancy Testing

Women of childbearing potential must have a negative pregnancy test at Screening and Day 1 to be eligible for administration of IP. Pregnancy testing will also be conducted as per the Time and Events Table (See Section 6.1) and at anytime during the trial when pregnancy is suspected.

6.4.8.2. Action to be Taken if Pregnancy Occurs

Any pregnancy that occurs during study participation must be reported using a clinical trial pregnancy form. To ensure subject safety, each pregnancy must be reported to the Sponsor within 2 weeks of learning of its occurrence. The pregnancy must be followed up to determine outcome (including premature termination) and status of mother and child. Pregnancy complications and elective terminations for medical reasons must be reported as an AE or SAE. Spontaneous abortions must be reported as an SAE.

Any SAE occurring in association with a pregnancy, brought to the investigator's attention after the subject has completed the study and considered by the investigator as possibly related to the Investigational Product, must be promptly reported to the Sponsor.

The Sponsor's central safety department will also forward this information to the Antiretroviral Pregnancy Registry. The international registry is jointly sponsored by manufacturers or licensees of antiretroviral products. Additional information and a list of participating manufacturers/licensees are available from http://apregistry.com/index.htm.

6.4.8.3. Time Period for Collecting Pregnancy Information

Information on the occurrence of pregnancies in female subjects will be collected over the period starting at Screening and ending at the final follow-up visit. Only those pregnancies that occur following the first dose of IP will be reported to the Sponsor.

Follow-up information will only be collected for pregnancies occurring from Day 1 to the final follow-up visit.

In addition, the investigator must attempt to collect pregnancy information on any female partners of male study subjects who become pregnant while the subject is enrolled in the study. Pregnancy information must be reported to the Sponsor as described above.

6.4.9. Time Period and Frequency of Detecting AEs and SAEs

The investigator or site staff is responsible for detecting, documenting and reporting events that meet the definition of an AE or SAE.

AEs will be collected from the start of Investigational Product and until the follow up contact.

SAEs will be collected over the same time period as stated above for AEs. However, any SAEs assessed **as related** to study participation (e.g., Investigational Product, protocol-mandated procedures, invasive tests, or change in existing therapy) or related to a Sponsor concomitant medication, will be recorded from the time a subject consents to participate in the study up to and including any follow up contact. All SAEs will be reported to the Sponsor within 24 hours, as indicated in Section 6.4.10.

6.4.10. Prompt Reporting of Serious Adverse Events and Other Events

SAEs, pregnancies and liver function abnormalities meeting pre-defined criteria will be reported promptly by the investigator to the Sponsor as described in the following table once the investigator determines that the event meets the protocol definition for that event.

	**Initial Reports**		**Follow-up Information on a Previous Report**	
**Type of Event**	**Time Frame**	**Documents**	**Time Frame**	**Documents**
All SAEs	24 hours	"SAE" data collection tool	24 hours	Updated "SAE" data collection tool
Pregnancy	2 Weeks	Pregnancy Notification Form	2 Weeks	Pregnancy Follow up Form
ALT≥3xULN **plus** Bilirubin≥2xULN (>35% direct) and/or INR>1.5	24 hours	SAE data collection tool	24 hours	Updated SAE data collection tool

The method of detecting, recording, evaluating and follow-up of AEs and SAEs plus procedures for completing and transmitting SAE reports to the Sponsor are provided in the SPM. Procedures for post-study AEs/SAEs are provided in the SPM.

6.4.10.1. Regulatory reporting requirements for SAEs

Prompt notification of SAEs by the investigator to the Sponsor is essential so that legal obligations and ethical responsibilities towards the safety of subjects are met.

The Sponsor has a legal responsibility to notify both the local regulatory authority and other regulatory agencies about the safety of a product under clinical investigation. The Sponsor will comply with country specific regulatory requirements relating to safety reporting to the regulatory authority, Institutional Review Board (IRB)/Independent Ethics Committee (IEC) and investigators.

Investigator safety reports are prepared for suspected unexpected serious adverse reactions according to local regulatory requirements and sponsor policy and are forwarded to investigators as necessary.

An investigator who receives an investigator safety report describing a SAE(s) or other specific safety information (e.g., summary or listing of SAEs) from the Sponsor will file it with the package insert and will notify the IRB/IEC, if appropriate according to local requirements.

6.5. Exploratory Biomarker Assessments

Blood and urine will be collected to perform various cardiovascular, bone and renal biomarker assessments at Baseline, Weeks 24, 48 and W/D. The assessments may or may not include markers such as high sensitivity C-reactive protein (hsCRP), interleukin-6 (IL-6), D-dimer, serum procollagen type 1 N-propeptide (P1NP), serum bone specific alkaline phosphatase (BSAP), serum parathyroid hormone (PTH), serum c-telopeptide (CTx), osteocalcin, vitamin D 1,25-OH, and beta-2 microglobulin. It is not anticipated that the Sponsor will provide the results of these assessments to the investigator.

6.6. Viral Genotyping and Phenotyping

Plasma samples will be prepared for storage at all study visits except Screening. Subjects experiencing virologic failure will also have samples drawn at the time the confirmatory sample is drawn for potential further analysis of the evolution of drug resistance-associated mutation selection. Confirmed virologic failures will be genotyped and phenotyped using plasma samples collected at the point of first failure detection. The point of failure genotype and phenotype results will be provided to the relevant investigator on a per-subject basis when available. Additional analyses may be performed on these stored samples to better understand what factors can influence therapy response, such as clonal analysis of samples from subjects meeting virologic failure criteria.

6.7. Pharmacogenetics

6.7.1. HLA-B*5701 Determination

At the Screening visit, a 10 mL blood sample will be collected for HLA-B*5701 determination using deoxyribonucleic acid (DNA)-based methods by a suitably accredited clinical laboratory. All subjects must provide a blood sample for HLA-B*5701 determination, as subjects who are HLA-B*5701 positive may not participate in the study. HLA-B*5701 results will be provided to study sites to determine subject eligibility for the study. As HLA-B*5701 status has potential clinical implications, subjects will be provided with their HLA-B*5701 results.

A second 10 mL blood sample will be collected for potential pharmacogenetics research aimed at understanding variation in subject response to the antiretroviral therapies included in this study. Information regarding pharmacogenetic research is included in Appendix 4: Pharmacogenetics. The IEC/IRB and, where required, the applicable regulatory agency must approve the PGx assessments before these can be conducted at the site. The approval(s) must be in writing and will clearly specify approval of the PGx assessments (see Appendix 4: Pharmacogenetics). In some cases, approval of the PGx assessments can occur after approval is obtained for the rest of the study. If so, then the written approval will clearly indicate approval of the PGx assessments is being deferred and the study, except for PGx assessments, can be initiated. When PGx assessments will not be approved, then the approval for the rest of the study will clearly indicate this and therefore, PGx assessments will not be conducted.

7. DATA MANAGEMENT

For this study subject data will be entered into electronic case report forms (eCRFs), transmitted electronically to the Sponsor and combined with data provided from other sources in a validated data system.

Management of clinical data will be performed in accordance with applicable Sponsor standards and data cleaning procedures to ensure the integrity of the data, e.g., removing errors and inconsistencies in the data. Adverse events and concomitant medications terms will be coded using the Medical Dictionary for Drug Regulatory Affairs (MedDRA) and an internal validated medication dictionary, GSKDrug. eCRFs (including queries and audit trails) will be retained by the Sponsor, and copies will be sent to the investigator to maintain as the investigator copy. In all cases, subject initials will not be collected or transmitted to the Sponsor according to Sponsor policy.

8. DATA ANALYSIS AND STATISTICAL CONSIDERATIONS

8.1. Hypotheses

The primary hypothesis of interest is that treatment with ABC/3TC + ATV is not inferior to treatment with TDF/FTC + ATV/RTV in the proportion of subjects with plasma HIV-1 RNA <50 c/mL over 24 weeks in a population of virologically suppressed HIV-1 infected subjects.

The null hypothesis is that ABC/3TC + ATV arm is inferior to TDF/FTC + ATV/RTV arm in proportion of subjects with HIV-1 RNA <50 c/mL at Week 24. This null hypothesis is to be tested against the alternative that ABC/3TC + ATV is at least as good as (i.e., not inferior to) TDF/FTC + ATV/RTV in proportion of subjects with HIV-1 RNA <50 c/mL at Week 24. With a non-inferiority margin of 0.12, the null and alternative hypothesis can be written statistically as

Ho: $p_1 – p_2 \leq -0.12$,

Ha: $p_1 – p_2 > -0.12$;

where p_1, p_2 are the proportions of subjects with HIV-1 RNA <50 c/mL at Week 24 in ABC/3TC + ATV and TDF/FTC + ATV/RTV, respectively.

8.2. Study Design Considerations

This is a phase IV, randomized, open-label, multi-center study evaluating the efficacy, safety, and tolerability of the antiviral response between ATV + ABC/3TC and ATV/RTV + TDF/FTC for 48 weeks in HIV-1 infected, HLA-B*5701 negative subjects who were previously suppressed on ATV/RTV + TDF/FTC.

8.2.1. Sample Size Assumptions

A sample size of 300 subjects with a 2:1 randomization ratio in ABC/3TC + ATV to TDF/FTC + ATV/RTV will provide 90% power (one-sided, α=0.025) to establish non-inferiority via a 95% confidence interval for the difference in proportions of subjects with HIV-1 RNA <50 c/mL at Week 24. This sample size calculation assumes a virologic response rate (proportions of subjects with HIV-1 RNA < 50 c/mL) of 89% in both treatment arms and a non-inferiority margin of 0.12.

8.2.2. Sample Size Sensitivity

The power of the study is affected by the assumptions of the proportion of virologic response in each treatment arm, type-I error, one or two sided test, and the non-inferiority margin. The non-inferiority margin has been set at 0.12 for the difference between treatment arms, and type-I error has been set at α=0.05, two sided (one sided α=0.025). The choice of delta margin of 0.12 in comparing response rate is consistent with previous non-inferiority studies including studies to support regulatory submissions.

Table 2 shows the sensitivity of the power change in the response rate assumptions with a total sample size of 300 subjects (200 in EPZICOM and 100 in Truvuda) using a non-inferiority margin of 0.12, 1-sided alpha of 0.025.

Table 2 Power changes as response rate varies (Fixed sample size: N=300)

EPZICOM Virologic Response Rate	Truvuda Virologic Response Rate	Power (1-sided at α=0.025)
90%	90%	92%
89%	89%	90%
88%	88%	88%
87%	87%	86%
86%	86%	84%
85%	85%	82%

Table 3 shows the sensitivity of the sample size change with varying response rates in order to achieve 90% based on a non-inferiority margin of 0.12 and 1-sided alpha of 0.025.

Table 3 Sample Size changes as response rate varies (Fixed Power: 90%)

Virologic Response Rate in both arms	Sample Size Epzicom:Truvuda = 2:1		
	N (EPZICOM)	N (Truvuda)	Total Sample Size
90%	183	92	275
89%	198	99	297
88%	213	107	320
87%	227	114	341
86%	242	121	363
85%	256	128	384

8.2.3. Sample Size Re-estimation

No sample size re-estimation is planned for this study.

8.3. Data Analysis Considerations

8.3.1. Analysis Populations

Intent-to-treat (ITT) population

The ITT population will consist of all enrolled subjects who are randomized in the study, regardless of what treatment is actually received or the eventual outcome of study participation. A modified ITT population, ITT-Exposed, that consists only of subjects who are in the ITT population and receive at least one dose of study drug, will be the primary population for efficacy analyses. If more than 5% of randomized subjects do not

receive treatment, then additional sensitivity analyses may be carried out with the ITT population defining subjects who withdraw without receiving treatment as failures.

Safety Population

The safety population will consist of all randomized subjects with the exception of those with documented evidence of not having consumed any Investigational Product.

Virologic Failure Population

The virologic failure population will consist of all subjects who met the protocol defined virologic failure criteria.

Subpopulations

Sub-populations may be created as subsets of the ITT population for use in supporting safety or efficacy analyses. Sub-populations of interest may include (but are not limited to) per protocol population, Baseline HIV-1 RNA strata, Baseline CD4+ category, demographic category, age and ethnic origin categories or genotypic subgroups.

8.3.2. Analysis Data Sets

Observed Dataset

The observed dataset contains all data collected while subjects are in the study. No missing values are imputed.

8.3.3. Treatment Comparisons

8.3.3.1. Primary Comparisons of Interest

For all study objectives, the primary comparison of interest will be between the two treatment arms: ABC/3TC + ATV and TDF/FTC + ATV/RTV. The treatment comparisons will be made at designated timepoints as specified in the defined endpoints.

For the primary efficacy endpoint, a two-sided 95% confidence interval (CI) approach will be used to test for non-inferiority between the two treatment arms as described further in the Primary Analysis Section 8.3.5.1.section below.

8.3.4. Interim Analysis

There are two planned analyses in this study: an interim analysis at Week 24 and a final analysis at Week 48. The Week 24 analysis is considered the primary analysis to establish non-inferiority.

8.3.5. Key Elements of Analysis Plan

8.3.5.1. Efficacy Analyses

All efficacy analysis will be performed based on the ITT-Exposed population.

Primary Analysis

The primary efficacy objective of the study is to establish the non-inferiority of the EPZICOM arm to the Truvada arm in proportion of subjects with plasma HIV-1 RNA <50 c/mL at Week 24.

Statistical hypothesis testing on the difference in the proportion of subjects with HIV-1 RNA <50 c/mL at Week 24 between the two treatment arms being -0.12 or less versus the alternative that the difference is > -0.12 will be performed at a one-sided 0.025 significance level. This is equivalent to constructing a two-sided 95% confidence interval on the difference in proportions and comparing the lower bound of the 95% CI to -0.12. The null hypothesis will be rejected and hence the non-inferiority will be established if the lower limit of the confidence interval for the difference (EPZICOM arm minus Truvada) in proportion of subjects with HIV-1 RNA <50 c/mL at Week 24 is > - 0.12. However, if the lower limit of the 95% CI is ≤ -0.12, it cannot be ruled out that the EPZICOM arm is inferior to the Truvada arm. The Cochran-Mantel-Haenszel (CMH) test will be used in the analysis. Odds ratios, confidence intervals, and p-values will be computed: (1) stratifying by initial antiretroviral regimen received (ATV/RTV + TDF/FTC as initial regimen or ATV/RTV + TDF/FTC as first or second switch regimen), (2) stratifying by center, and (3) without stratification.

Secondary Analysis

The proportion of subjects with plasma HIV-1 RNA <400 c/mL and <50 c/mL will be calculated and summarized at each assessment week. TLOVR, Observed and M=F rates will be computed by treatment arm. The rates will be compared between treatment arms using the CMH test stratified by initial antiretroviral regimen received at the planned analysis timepoints.

Summary statistics for the measured values and change from Baseline in HIV-1 RNA will also be tabulated at each assessment week by treatment arm.

The immunologic response will be assessed with summary statistics for CD4+ and CD8+ cell count measured value and change from Baseline at each assessment week by treatment arm. These parameters may be compared between treatment arms using the Wilcoxon sum-rank test as exploratory analyses.

Time to loss of virologic response (TLOVR) will be analyzed using the Kaplan-Meier non-parametric method and plotted by treatment groups.

TLOVR algorithm:

For 1 and 2 below, all visits with no data will be discarded. In what follows, a visit means a visit with an observed viral load. Viral load data from all available visits, including off-schedule visits will be included in the calculation.

If a subject has never achieved confirmed HIV-1 RNA levels <400 (50) c/mL (on two consecutive visits) before the following events, then this subject will be considered to have failed at time 0:

- Death
- Permanent discontinuation of study, study medication or any modifications of the regimen for any reason other than ABC HSR.
- By the planned analysis week (Week 24 or 48).

For all subjects who had confirmed HIV-1 RNA levels <400 (50) c/mL, on two consecutive visits, the time of failure is the earliest time when any of the following specific event have occurred:

- Death
- Permanent discontinuation of study, study medication or any modifications of regimen for any reason other than ABC HSR
- Confirmed HIV-1 RNA levels ≥400 (50) c/mL (time to the first confirmed HIV-1 RNA ≥400 (50) c/mL) or HIV-1 RNA levels ≥400 (50) c/mL at one visit followed by loss to follow-up (including any discontinuation or just last visit)

If the time of failure as defined above is immediately preceded by a single missing scheduled visit or multiple consecutive missing scheduled visits, then the time of failure is replaced by the first time of such missing visits.

Subjects who complete the planned analysis weeks (24, 48) of the study without experiencing a loss of virologic response will be right censored at that point.

8.3.5.2. Safety Analyses

All safety analyses will be based on the safety population.

Extent of Exposure

Extent of exposure will be summarized according to the cumulative number of days a subject was taking each Investigational Product and the maximum time the subject spent on any Investigational Product (i.e., the difference from the first day on any investigation product until the last day on any Investigational Product). Summary statistics for the extent of exposure to each specific Investigational Product will be provided.

Adverse Events

Adverse event data will be coded using the MedDRA coding dictionary and the resulting terms will be categorized by system organ class. A listing showing the relationship of AE, system organ class, group preferred terms and verbatim text will be presented. Adverse events (with onset on or after the first dose of Investigational Product or with onset before the first dose of Investigational Product but worsens during the study) will be tabulated by treatment arm for:

- All Grade 2-4 adverse events.
- Grade 3-4 adverse events.
- Treatment-related adverse events.
- Adverse events leading to premature discontinuation from the study.
- Serious adverse events.

Additionally, AEs will be tabulated by maximum intensity and action taken with respect to Investigational Product. The number and percent of subjects experiencing a treatment-limiting adverse event will also be presented by treatment arm.

A listing of deaths and SAEs that occur during the study will be generated. A listing of subjects who experience HSR will also be generated.

Exploratory statistical analyses of the AE data may be performed, as deemed appropriate. These analyses will be detailed in the reporting and analysis plan (RAP) for the study.

Clinical Laboratory Evaluations

Blood chemistry, haematology, and urine analytes will be summarized by treatment arm after conversion to standard units (as necessary). The laboratory data will be summarized as follows:

- Descriptive summary statistics will be presented by treatment arm and week for each laboratory parameter and change from Baseline.
- Summary of toxicity shifts from Baseline: the maximum post-Baseline toxicity grade will be tabulated (count and percent) against the Baseline toxicity grade for each laboratory parameter by treatment arm.
- Listing of laboratory data for subjects with at least one laboratory toxicity of Grade 3 or Grade 4 will be displayed by laboratory parameter, treatment arm, subject, and week.

Baseline is defined as the last pre-treatment value collected. Subjects must have both a Baseline and on-treatment measurement to be included in the change from Baseline analysis.

8.3.5.3. Viral Genotyping/Phenotyping Analyses

For the virologic failure population, only samples from subjects who meet the criteria for virologic failure and have genotypic or phenotypic resistance data will be included. As the absolute number of virologic failures is likely to be small, the statistical analyses will be mainly descriptive. Genotypic and phenotypic resistance data will be summarized by treatment arm at the time of virologic failure. Summaries of genotypic data will be based mainly on the mutations provided in the IAS USA Resistance Tables (based on the most current version). Correlation between phenotypic resistance, genotypic mutations, and HIV-1 RNA responses may be explored.

9. STUDY CONDUCT CONSIDERATIONS

9.1. Posting of Information on Clinicaltrials.gov

Study information from this protocol will be posted on clinicaltrials.gov before subject enrolment begins.

9.2. Regulatory and Ethical Considerations, Including the Informed Consent Process

Prior to initiation of a study site, the Sponsor will obtain approval from the appropriate regulatory agency to conduct the study in accordance with applicable country-specific regulatory requirements, including those required under an US Investigational New Drug (IND).

The study will be conducted in accordance with all applicable regulatory requirements, [including an US IND].

The study will be conducted in accordance with Good Clinical Practice (GCP), all applicable subject privacy requirements, and the ethical principles that are outlined in the Declaration of Helsinki 2008, including, but not limited to:

- Institutional Review Board (IRB)/Independent Ethics Committee (IEC) review and approval of study protocol and any subsequent amendments.
- Subject informed consent.
- Investigator reporting requirements.

The Sponsor will provide full details of the above procedures, either verbally, in writing, or both.

Written informed consent must be obtained from each subject prior to participation in the study.

9.3. Quality Control (Study Monitoring)

In accordance with applicable regulations, GCP, and Sponsor procedures, monitors will contact the site prior to the start of the study to review with the site staff the protocol, study requirements, and their responsibilities to satisfy regulatory, ethical, and Sponsor requirements. When reviewing data collection procedures, the discussion will include identification, agreement and documentation of data items for which the eCRF will serve as the source document.

The Sponsor will monitor the study to ensure that the:

- Data are authentic, accurate, and complete.
- Safety and rights of subjects are being protected.
- Study is conducted in accordance with the currently approved protocol and any other study agreements, GCP, and all applicable regulatory requirements.

The investigator and the head of the medical institution (where applicable) agrees to allow the monitor direct access to all relevant documents.

9.4. Quality Assurance

To ensure compliance with GCP and all applicable regulatory requirements, the Sponsor may conduct a quality assurance audit of the site records, and the regulatory agencies may conduct a regulatory inspection at any time during or after completion of the study. In the event of an audit or inspection, the investigator (and institution) must agree to grant the auditor(s) and inspector(s) direct access to all relevant documents and to allocate their time and the time of their staff to discuss any findings/relevant issues.

9.5. Study and Site Closure

Upon completion or termination of the study, the monitor will conduct site closure activities with the investigator or site staff (as appropriate), in accordance with applicable regulations, GCP, and Sponsor Standard Operating Procedures.

The Sponsor reserves the right to temporarily suspend or terminate the study at any time for reasons including (but not limited to) safety issues, ethical issues, or severe non-compliance. If the Sponsor determines that such action is required, the Sponsor will discuss the reasons for taking such action with the investigator or head of the medical institution (where applicable). When feasible, the Sponsor will provide advance notice to the investigator or head of the medical institution of the impending action.

If a study is suspended or terminated for **safety reasons**, the Sponsor will promptly inform all investigators, heads of the medical institutions (where applicable),and/or institutions conducting the study. The Sponsor will also promptly inform the relevant regulatory authorities of the suspension/termination along with the reasons for such action. Where required by applicable regulations, the investigator or head of the medical

institution must inform the IRB/IEC promptly and provide the reason(s) for the suspension/termination.

9.6. Records Retention

Following closure of the study, the investigator or head of the medical institution (where applicable) must maintain all site study records (except for those required by local regulations to be maintained elsewhere) in a safe and secure location. The records must be easily accessible when needed (e.g., for a Sponsor audit or regulatory inspection) and must be available for review in conjunction with assessment of the facility, supporting systems, and relevant site staff.

Where permitted by local laws/regulations or institutional policy, some or all of the records may be maintained in a format other than hard copy (e.g., microfiche, scanned, electronic); however, caution must be exercised before such action is taken. The investigator must ensure that all reproductions are legible and are a true and accurate copy of the original. In addition, they must meet accessibility and retrieval standards, including regeneration of a hard copy, if required. The investigator must also ensure that an acceptable back-up of the reproductions exists and that there is an acceptable quality control procedure in place for creating the reproductions.

The Sponsor will inform the investigator of the time period for retaining the site records in order to comply with all applicable regulatory requirements. The minimum retention time will meet the strictest standard applicable to a particular site, as dictated by local laws/regulations, Sponsor standard operating procedures, and/or institutional requirements.

The investigator must notify the Sponsor of any changes in the archival arrangements, including, but not limited to archival of records at an off-site facility or transfer of ownership of the records in the event that the investigator is no longer associated with the site.

9.7. Provision of Study Results to Investigators, Posting to the Clinical Trials Register and Publication

Where required by applicable regulatory requirements, an investigator signatory will be identified for the approval of the clinical study report. The investigator will be provided reasonable access to statistical tables, figures, and relevant reports and will have the opportunity to review the complete study results at a Sponsor site or other mutually-agreeable location.

The Sponsor will also provide the investigator with the full summary of the study results. The investigator is encouraged to share the summary results with the study subjects, as appropriate.

The Sponsor will provide the investigator with the randomization codes for their site only after completion of the full statistical analysis.

The results summary will be posted to the Clinical Study Register no later than 12 months after the last subject's last visit (LSLV) or sooner if required by legal agreement, local law or regulation. In addition, a manuscript will be submitted to a peer-reviewed journal for publication within 18 months of LSLV. When manuscript publication in a peer-reviewed journal is not feasible, further study information will be posted to the Sponsor's Clinical Study Register to supplement the results summary.

A manuscript will be progressed for publication in the scientific literature if the results provide important scientific or medical knowledge.

10. REFERENCES

Cockroft DW, Gault MH. Prediction of creatinine clearance from serum creatinine. Nephron 1976;16:31-41.

Delfraissy JF, Moreno S, Sanz-Moreno J, Carosi G, et al. Efficacy and safety of 48 weeks of maintenance with once-daily ATV vs. ATV/r in patients with viral load <50 c/mL after induction with ATV/r. Presented at the 9th Biennial International Congress on Drug Therapy in HIV Infection. Glasgow, UK, 2008:Abstract 042.

Dubé MP, Stein JH, Aberg JA, et al. Adult AIDS Clinical Trials Group Cardiovascular Subcommittee; HIV Medical Association of the Infectious Disease Society of America. Guidelines for the evaluation and management of dyslipidemia in human immunodeficiency virus (HIV)-infected adults receiving antiretroviral therapy: recommendations of the HIV Medical Association of the Infectious Disease Society of America and the Adult AIDS Clinical Trials Group. Clin Infect Dis 2003;37:613-27.

EPZICOM (abacavir sulfate/lamivudine) Product Information. March, 2009.

Fisher EJ, Chaloner K, Cohn DL, et al. The safety and efficacy of adefovir dipivoxil in patients with advanced HIV disease: a randomized, placebo-controlled trial. AIDS 2001;15:1695-1700.

Gatell J, Salmon-Ceron D, Lazzarin A, et al. Efficacy and safety of atazanavir-based highly active antiretroviral therapy in patients with virologic suppression switched from a stable, boosted or unboosted protease inhibitor treatment regimen: The SWAN Study (AI424-097) 48-week results. *Clin Infect Dis* 2007;44:1484–1492.

Gatell JM, Branco T, Sasset L, et al. Efficacy and safety of atazanavir (ATV) based HAART in virologically suppressed subjects switched from lopinavir/ritonavir (LPV/RTV) treatment. Oral abstract session: AIDS 2006 – XVI International AIDS Conference : Abstract no. THPE0123.

Giuntini R, Martinelli GR, Ricci E, et al. Efficacy and safety of boosted and unboosted atazanavir-containing antiretroviral regimens in real life: results from a multicentre cohort study. HIV Med 2010;11:40-5.

Malan N, Krantz E, David N, et al. Efficacy and Safety of Atazanavir-based Therapy in Antiretroviral Naïve HIV-1 Infected Subjects, Both with and without Ritonavir: 48-week Results from AI424-089. Abstract 79. 13th Conference on Retroviruses and Opportunistic Infections, 05 Feb-09 Feb, 2006; Denver, CO, USA.

Mallal S, Phillips E, Carosi G, et al for the PREDICT-1 Study Team. HLA-B*5701 screening for hypersensitivity to abacavir. N Engl J Med. 2008;358:568-79.

McGrath D, Hammond J, Frederick D, et al. Evaluation of resistance patterns in treatment naïve subjects with virological failure on atazanavir- or atazanavir/ritonavir-containing regimens. *Antivir Ther* 2006; 11:S97 (abstract 87)

National Kidney Foundation. K/DOQI clinical practice guidelines for chronic kidney disease: evaluation, classification and stratification. Am J Kidney Dis 2002;39(suppl 1):S1-266.

Norvir (ritonavir) Product Information. April, 2010.

Palella F, Delaney K, Moorman A, et al. Declining morbidity and mortality among patients with advanced human immunodeficiency virus infection. *N Engl J Med.* 1998;338:853-860.

Panel on Antiretroviral Guidelines for Adults and Adolescents. Guidelines for the use of antiretroviral agents in HIV-1-infected adults and adolescents. Department of Health and Human Services (DHHS). December 1, 2009; 1-161. Available at http://www.aidsinfo.nih.gov/ContentFiles/AdultandAdolescentGL.pdf.

Pavie J, Rachline A, Porcher R, et al. Long-term efficacy and safety of a switch to unboosted atazanavir-based regimens among HIV-infected patients with suppression of viral replication: a retrospective cohort study. In: Program and abstracts of the 5th International AIDS Society Conference on HIV Pathogenesis, Treatment and Prevention; July 19-22, 2009; Cape Town, South Africa. Poster CDB089.

Reyataz (atazanavir) Product Information. April, 2010.

Saag M, Balu R, Phillips E, et al. for the Study of Hypersensitivity to Abacavir and Pharmacogenetic Evaluation Study Team. High sensitivity of human leukocyte antigen-b*5701 as a marker for immunologically confirmed abacavir hypersensitivity in white and black patients. Clin Infect Dis 2008;46:1111-8.

Santos JR, Molto J, Llibre JM, et al. Unboosted atazanavir plus co-formulated lamivudine/abacavir as a ritonavir-sparing simplification strategy in routine clinical practice. HIV Clin Trials 2009; 10:129-134.

Squires KE, Young B, DeJesus E, et al. Similar efficacy and tolerability of atazanavir compared with atazanavir/ritonavir, each with abacavir/lamivudine after initial suppression with abacavir/lamivudine plus ritonavir-boosted atazanavir in HIV-1 infected patients. AIDS 2010(a);13:2019-27.

Squires K, DeJesus E, Bellos N, et al. Sustained virologic efficacy of atazanavir (ATV) versus atazanavir/ritonavir (ATV/ r), each in combination with abacavir/lamivudine (ABC/3TC)over 120 weeks: the ARIES trial. In: Program and abstracts of the 10th Annual Interscience Conference on Antimicrobial Agents and Chemotherapy (ICAAC); September 12-15, 2010(b); Boston, MA. Poster H-204.

Sun HY, Hung CC, Lin PH, et al. Incidence of abacavir hypersensitivity and its relationship with HLA-B*5701 in HIV-infected patients in Taiwan. J Antimicrob Chemother 2007; 60:599-604.

11. APPENDICES

11.1. Appendix 1: CDC Classification CDC Classification System for HIV-1 Infections (1993)

Reference - 1993 revised classification system for HIV infection and expanded surveillance case definition for AIDS among adolescents and adults.
MMWR 1992;41(No. RR-17):1-19.

Clinical Categories

The clinical categories of HIV infection are defined as follows:

Category A

Category A consists of one or more of the conditions listed below in an adolescent or adult (>13 years) with documented HIV infection. Conditions listed in Categories B and C must not have occurred.

- Asymptomatic HIV infection
- Persistent generalized lymphadenopathy
- Acute (primary) HIV infection with accompanying illness or history of acute HIV infection

Category B (Symptomatic non-AIDS conditions)

Category B consists of symptomatic conditions in an HIV-infected adolescent or adult that are not included among conditions listed in clinical Category C and that meet at least one of the following criteria: a) the conditions are attributed to HIV infection or are indicative of a defect in cell-mediated immunity; or b) the conditions are considered by physicians to have a clinical course or to require management that is complicated by HIV infection. **Examples** of conditions in clinical Category B include, **but are not limited to:**

- Bacillary angiomatosis
- Candidiasis, oropharyngeal (thrush)
- Candidiasis, vulvovaginal; persistent, frequent, or poorly responsive to therapy
- Cervical dysplasia (moderate or severe)/cervical carcinoma in situ
- Constitutional symptoms, such as fever (38.5°C) or diarrhea lasting >1 month
- Hairy leukoplakia, oral
- Herpes zoster (shingles), involving at least two distinct episodes or more than one dermatome
- Idiopathic thrombocytopenic purpura

- Listeriosis
- Pelvic inflammatory disease, particularly if complicated by tubo-ovarian abscess
- Peripheral neuropathy

For classification purposes, Category B conditions take precedence over those in Category A. For example, someone previously treated for oral or persistent vaginal candidiasis (and who has not developed a Category C disease) but who is now asymptomatic should be classified in clinical Category B.

Category C (AIDS indicator conditions as defined by diagnostic or presumptive measures).

Category C includes the clinical conditions listed in the AIDS surveillance case definition. For classification purposes, once a Category C condition has occurred, the person will remain in Category C.

Conditions in Category C include:

- Candidiasis of bronchi, trachea, or lungs
- Candidiasis, esophageal
- Cervical cancer, invasive
- Coccidioidomycosis, disseminated or extrapulmonary
- Cryptococcosis, extrapulmonary
- Cryptosporidiosis, chronic intestinal (>1 month's duration)
- Cytomegalovirus disease (other than liver, spleen, or nodes)
- Cytomegalovirus retinitis (with loss of vision)
- Encephalopathy, HIV-related
- Herpes simplex: chronic ulcer(s) (>1 month's duration);or bronchitis, pneumonitis, or esophagitis
- Histoplasmosis, disseminated or extrapulmonary
- Isosporiasis, chronic intestinal (>1 month's duration)
- Kaposi's sarcoma
- Lymphoma, Burkitt's (or equivalent term)
- Lymphoma, immunoblastic (or equivalent term)
- Lymphoma, primary, of brain
- *Mycobacterium avium* complex or *M. kansasii*, disseminated or extrapulmonary
- *Mycobacterium tuberculosis*, any site (pulmonary or extrapulmonary)
- *Mycobacterium*, other species or unidentified species, disseminated or extrapulmonary

- *Pneumocystis carinii* pneumonia
- Pneumonia, recurrent
- Progressive multifocal leukoencephalopathy
- *Salmonella* septicemia, recurrent
- Toxoplasmosis of brain
- Wasting syndrome due to HIV
- Non-CDC, HIV-associated conditions.

11.2. Appendix 2: CogState

Research concerning cognition is still in its infancy. As we discover more about the subject, cognitive testing plays an increasingly important role - especially in clinical trials.

CogState ClinicalTrials tasks provide rapid, sensitive and valid measurement of distinct cognitive functions. The tasks use novel visual and verbal stimuli to ensure assessment is culture-neutral and not limited by a subject's level of education. All CogState tasks are designed for repeated administration with minimal practice or learning effects, making them ideal for use in clinical research trials.

A CogState ClinicalTrials test battery comprises a number of individual tasks - each designed to test a specific area of cognition. When a number of these individual tasks are put together to form a test battery, a more complete picture of a person's cognitive state can be garnered.

All CogState test batteries run on standard computer equipment and allow for non-expert administration. It is estimated that the battery used for this study should take approximately 15 minutes to complete.

CogState is fully compliant with FDA 21 CFR Part 11 and all quality and data systems have been audited successfully by independent consultants and global pharmaceutical companies.

Additional information and instruction will be provided in the Study Procedures Manual.

For further information, go to http://www.cogstate.com/.

11.3. Appendix 3: DAIDS Division of Aids Table for Grading the Severity of Adult and Pediatric Adverse Events

Version 1.0, December, 2004; clarification August 2009

PARAMETER	GRADE 1 MILD	GRADE 2 MODERATE	GRADE 3 SEVERE	GRADE 4 POTENTIALLY LIFE-THREATENING
ESTIMATING SEVERITY GRADE				
Clinical adverse event NOT identified elsewhere in this DAIDS AE Grading Table	Symptoms causing no or minimal interference with usual social & functional activities	Symptoms causing greater than minimal interference with usual social & functional activities	Symptoms causing inability to perform usual social & functional activities	Symptoms causing inability to perform basic self-care functions OR Medical or operative intervention indicated to prevent permanent impairment, persistent disability, or death
SYSTEMIC				
Acute systemic allergic reaction	Localized urticaria (wheals) with no medical intervention indicated	Localized urticaria with medical intervention indicated OR Mild angioedema with no medical intervention indicated	Generalized urticaria OR Angioedema with medical intervention indicated OR Symptomatic mild bronchospasm	Acute anaphylaxis OR Life-threatening bronchospasm OR laryngeal edema
Chills	Symptoms causing no or minimal interference with usual social & functional activities	Symptoms causing greater than minimal interference with usual social & functional activities	Symptoms causing inability to perform usual social & functional activities	NA

PARAMETER	GRADE 1 MILD	GRADE 2 MODERATE	GRADE 3 SEVERE	GRADE 4 POTENTIALLY LIFE-THREATENING
Fatigue<br><br>Malaise	Symptoms causing no or minimal interference with usual social & functional activities	Symptoms causing greater than minimal interference with usual social & functional activities	Symptoms causing inability to perform usual social & functional activities	Incapacitating fatigue/ malaise symptoms causing inability to perform basic self-care functions
Fever (nonaxillary)	37.7 – 38.6°C	38.7 – 39.3°C	39.4 – 40.5°C	> 40.5°C
Pain (indicate body site)<br><br>DO NOT use for pain due to injection (See Injection Site Reactions: Injection site pain)<br><br>See also Headache, Arthralgia, and Myalgia	Pain causing no or minimal interference with usual social & functional activities	Pain causing greater than minimal interference with usual social & functional activities	Pain causing inability to perform usual social & functional activities	Disabling pain causing inability to perform basic self-care functions OR Hospitalization (other than emergency room visit) indicated
Unintentional weight loss	NA	5 – 9% loss in body weight from baseline	10 – 19% loss in body weight from baseline	≥ 20% loss in body weight from baseline OR Aggressive intervention indicated [e.g., tube feeding or total parenteral nutrition (TPN)]

PARAMETER		GRADE 1<br>MILD	GRADE 2<br>MODERATE	GRADE 3<br>SEVERE	GRADE 4<br>POTENTIALLY LIFE-THREATENING
INFECTION					
Infection (any other than HIV infection)		Localized, no systemic antimicrobial treatment indicated AND Symptoms causing no or minimal interference with usual social & functional activities	Systemic antimicrobial treatment indicated OR Symptoms causing greater than minimal interference with usual social & functional activities	Systemic antimicrobial treatment indicated AND Symptoms causing inability to perform usual social & functional activities OR Operative intervention (other than simple incision and drainage) indicated	Life-threatening consequences (e.g., septic shock)
**INJECTION SITE REACTIONS**					
Injection site pain (pain without touching)<br><br>Or<br><br>Tenderness (pain when area is touched)		Pain/tenderness causing no or minimal limitation of use of limb	Pain/tenderness limiting use of limb OR Pain/tenderness causing greater than minimal interference with usual social & functional activities	Pain/tenderness causing inability to perform usual social & functional activities	Pain/tenderness causing inability to perform basic self-care function OR Hospitalization (other than emergency room visit) indicated for management of pain/tenderness
Injection site reaction (localized)					
	**Adult > 15 years**	Erythema OR Induration of 5x5 cm – 9x9 cm (or 25 cm^2 – 81cm^2)	Erythema OR Induration OR Edema > 9 cm any diameter (or > 81 cm^2)	Ulceration OR Secondary infection OR Phlebitis OR Sterile abscess OR Drainage	Necrosis (involving dermis and deeper tissue)

PARAMETER	GRADE 1 MILD	GRADE 2 MODERATE	GRADE 3 SEVERE	GRADE 4 POTENTIALLY LIFE-THREATENING
**Pediatric ≤ 15 years**	Erythema OR Induration OR Edema present but ≤ 2.5 cm diameter	Erythema OR Induration OR Edema > 2.5 cm diameter but < 50% surface area of the extremity segment (e.g., upper arm/thigh)	Erythema OR Induration OR Edema involving ≥ 50% surface area of the extremity segment (e.g., upper arm/thigh) OR Ulceration OR Secondary infection OR Phlebitis OR Sterile abscess OR Drainage	Necrosis (involving dermis and deeper tissue)
Pruritis associated with injection<br><br>See also Skin: Pruritis (itching - no skin lesions)	Itching localized to injection site AND Relieved spontaneously or with < 48 hours treatment	Itching beyond the injection site but not generalized OR Itching localized to injection site requiring ≥ 48 hours treatment	Generalized itching causing inability to perform usual social & functional activities	NA
SKIN – DERMATOLOGICAL				
Alopecia	Thinning detectable by study participant (or by caregiver for young children and disabled adults)	Thinning or patchy hair loss detectable by health care provider	Complete hair loss	NA

PARAMETER	GRADE 1<br>MILD	GRADE 2<br>MODERATE	GRADE 3<br>SEVERE	GRADE 4<br>POTENTIALLY LIFE-THREATENING
Cutaneous reaction – rash	Localized macular rash	Diffuse macular, maculopapular, or morbilliform rash OR Target lesions	Diffuse macular, maculopapular, or morbilliform rash with vesicles or limited number of bullae OR Superficial ulcerations of mucous membrane limited to one site	Extensive or generalized bullous lesions OR Stevens-Johnson syndrome OR Ulceration of mucous membrane involving two or more distinct mucosal sites OR Toxic epidermal necrolysis (TEN)
Hyperpigmentation	Slight or localized	Marked or generalized	NA	NA
Hypopigmentation	Slight or localized	Marked or generalized	NA	NA
Pruritis (itching – no skin lesions)<br><br>(See also Injection Site Reactions: Pruritis associated with injection)	Itching causing no or minimal interference with usual social & functional activities	Itching causing greater than minimal interference with usual social & functional activities	Itching causing inability to perform usual social & functional activities	NA
CARDIOVASCULAR				
Cardiac arrhythmia (general)<br><br>(By ECG or physical exam)	Asymptomatic AND No intervention indicated	Asymptomatic AND Non-urgent medical intervention indicated	Symptomatic, non-life-threatening AND Non-urgent medical intervention indicated	Life-threatening arrhythmia OR Urgent intervention indicated

PARAMETER		GRADE 1 MILD	GRADE 2 MODERATE	GRADE 3 SEVERE	GRADE 4 POTENTIALLY LIFE-THREATENING
Cardiac-ischemia/infarction		NA	NA	Symptomatic ischemia (stable angina) OR Testing consistent with ischemia	Unstable angina OR Acute myocardial infarction
Hemorrhage (significant acute blood loss)		NA	Symptomatic AND No transfusion indicated	Symptomatic AND Transfusion of ≤ 2 units packed RBCs (for children ≤ 10 cc/kg) indicated	Life-threatening hypotension OR Transfusion of > 2 units packed RBCs (for children > 10 cc/kg) indicated
Hypertension					
	**Adult > 17 years**<br><br>(with repeat testing at same visit)	140 – 159 mmHg systolic<br><br>OR<br><br>90 – 99 mmHg diastolic	160 – 179 mmHg systolic<br><br>OR<br><br>100 – 109 mmHg diastolic	≥ 180 mmHg systolic<br><br>OR<br><br>≥ 110 mmHg diastolic	Life-threatening consequences (e.g., malignant hypertension) OR Hospitalization indicated (other than emergency room visit)
	**Correction**: in Grade 2 to 160 - 179 from > 160-179 (systolic) and to ≥ 100 -109 from > 100-109 (diastolic) and<br><br>in Grade 3 to ≥ 180 from > 180 (systolic) and to ≥ 110 from > 110 (diastolic).				
	**Pediatric ≤ 17 years**<br><br>(with repeat testing at same visit)	NA	91st – 94th percentile adjusted for age, height, and gender (systolic and/or diastolic)	≥ 95th percentile adjusted for age, height, and gender (systolic and/or diastolic)	Life-threatening consequences (e.g., malignant hypertension) OR Hospitalization indicated (other than emergency room visit)

PARAMETER		GRADE 1 MILD	GRADE 2 MODERATE	GRADE 3 SEVERE	GRADE 4 POTENTIALLY LIFE-THREATENING
Hypotension		NA	Symptomatic, corrected with oral fluid replacement	Symptomatic, IV fluids indicated	Shock requiring use of vasopressors or mechanical assistance to maintain blood pressure
Pericardial effusion		Asymptomatic, small effusion requiring no intervention	Asymptomatic, moderate or larger effusion requiring no intervention	Effusion with non-life threatening physiologic consequences OR Effusion with non-urgent intervention indicated	Life-threatening consequences (e.g., tamponade) OR Urgent intervention indicated
Prolonged PR interval					
	**Adult > 16 years**	PR interval 0.21 – 0.25 sec	PR interval > 0.25 sec	Type II 2nd degree AV block OR Ventricular pause > 3.0 sec	Complete AV block
	**Pediatric ≤ 16 years**	1st degree AV block (PR > normal for age and rate)	Type I 2nd degree AV block	Type II 2nd degree AV block	Complete AV block
Prolonged QTc					
	**Adult > 16 years**	Asymptomatic, QTc interval 0.45 – 0.47 sec OR Increase interval < 0.03 sec above baseline	Asymptomatic, QTc interval 0.48 – 0.49 sec OR Increase in interval 0.03 – 0.05 sec above baseline	Asymptomatic, QTc interval ≥ 0.50 sec OR Increase in interval ≥ 0.06 sec above baseline	Life-threatening consequences, e.g. Torsade de pointes or other associated serious ventricular dysrhythmia

PARAMETER	GRADE 1 MILD	GRADE 2 MODERATE	GRADE 3 SEVERE	GRADE 4 POTENTIALLY LIFE-THREATENING
**Pediatric ≤ 16 years**	Asymptomatic, QTc interval 0.450 – 0.464 sec	Asymptomatic, QTc interval 0.465 – 0.479 sec	Asymptomatic, QTc interval ≥ 0.480 sec	Life-threatening consequences, e.g. Torsade de pointes or other associated serious ventricular dysrhythmia
Thrombosis/embolism	NA	Deep vein thrombosis AND No intervention indicated (e.g., anticoagulation, lysis filter, invasive procedure)	Deep vein thrombosis AND Intervention indicated (e.g., anticoagulation, lysis filter, invasive procedure)	Embolic event (e.g., pulmonary embolism, life-threatening thrombus)
Vasovagal episode (associated with a procedure of any kind)	Present without loss of consciousness	Present with transient loss of consciousness	NA	NA
Ventricular dysfunction (congestive heart failure)	NA	Asymptomatic diagnostic finding AND intervention indicated	New onset with symptoms OR Worsening symptomatic congestive heart failure	Life-threatening congestive heart failure
GASTROINTESTINAL				
Anorexia	Loss of appetite without decreased oral intake	Loss of appetite associated with decreased oral intake without significant weight loss	Loss of appetite associated with significant weight loss	Life-threatening consequences OR Aggressive intervention indicated [e.g., tube feeding or total parenteral nutrition (TPN)]
**Comment:** Please note that, while the grading scale provided for Unintentional Weight Loss may be used as a guideline when grading anorexia, this is not a requirement and should not be used as a substitute for clinical judgment.				

PARAMETER		GRADE 1 MILD	GRADE 2 MODERATE	GRADE 3 SEVERE	GRADE 4 POTENTIALLY LIFE-THREATENING
Ascites		Asymptomatic	Symptomatic AND Intervention indicated (e.g., diuretics or therapeutic paracentesis)	Symptomatic despite intervention	Life-threatening consequences
Cholecystitis		NA	Symptomatic AND Medical intervention indicated	Radiologic, endoscopic, or operative intervention indicated	Life-threatening consequences (e.g., sepsis or perforation)
Constipation		NA	Persistent constipation requiring regular use of dietary modifications, laxatives, or enemas	Obstipation with manual evacuation indicated	Life-threatening consequences (e.g., obstruction)
Diarrhea					
	**Adult and Pediatric ≥ 1 year**	Transient or intermittent episodes of unformed stools OR Increase of ≤ 3 stools over baseline per 24-hour period	Persistent episodes of unformed to watery stools OR Increase of 4 – 6 stools over baseline per 24-hour period	Bloody diarrhea OR Increase of ≥ 7 stools per 24-hour period OR IV fluid replacement indicated	Life-threatening consequences (e.g., hypotensive shock)
	**Pediatric < 1 year**	Liquid stools (more unformed than usual) but usual number of stools	Liquid stools with increased number of stools OR Mild dehydration	Liquid stools with moderate dehydration	Liquid stools resulting in severe dehydration with aggressive rehydration indicated OR Hypotensive shock

PARAMETER	GRADE 1<br>MILD	GRADE 2<br>MODERATE	GRADE 3<br>SEVERE	GRADE 4<br>POTENTIALLY LIFE-THREATENING
Dysphagia-Odynophagia	Symptomatic but able to eat usual diet	Symptoms causing altered dietary intake without medical intervention indicated	Symptoms causing severely altered dietary intake with medical intervention indicated	Life-threatening reduction in oral intake
Mucositis/stomatitis (clinical exam)<br><br>Indicate site (e.g., larynx, oral)<br><br>See Genitourinary for Vulvovaginitis<br><br>See also Dysphagia-Odynophagia and Proctitis	Erythema of the mucosa	Patchy pseudomembranes or ulcerations	Confluent pseudomembranes or ulcerations OR Mucosal bleeding with minor trauma	Tissue necrosis OR Diffuse spontaneous mucosal bleeding OR Life-threatening consequences (e.g., aspiration, choking)
Nausea	Transient (< 24 hours) or intermittent nausea with no or minimal interference with oral intake	Persistent nausea resulting in decreased oral intake for 24 – 48 hours	Persistent nausea resulting in minimal oral intake for > 48 hours OR Aggressive rehydration indicated (e.g., IV fluids)	Life-threatening consequences (e.g., hypotensive shock)
Pancreatitis	NA	Symptomatic AND Hospitalization not indicated (other than emergency room visit)	Symptomatic AND Hospitalization indicated (other than emergency room visit)	Life-threatening consequences (e.g., circulatory failure, hemorrhage, sepsis)

PARAMETER	GRADE 1<br>MILD	GRADE 2<br>MODERATE	GRADE 3<br>SEVERE	GRADE 4<br>POTENTIALLY LIFE-THREATENING
Proctitis (functional-symptomatic)<br><br>Also see Mucositis/stomatitis for clinical exam	Rectal discomfort AND No intervention indicated	Symptoms causing greater than minimal interference with usual social & functional activities OR Medical intervention indicated	Symptoms causing inability to perform usual social & functional activities OR Operative intervention indicated	Life-threatening consequences (e.g., perforation)
Vomiting	Transient or intermittent vomiting with no or minimal interference with oral intake	Frequent episodes of vomiting with no or mild dehydration	Persistent vomiting resulting in orthostatic hypotension OR Aggressive rehydration indicated (e.g., IV fluids)	Life-threatening consequences (e.g., hypotensive shock)
NEUROLOGIC				
Alteration in personality-behavior or in mood (e.g., agitation, anxiety, depression, mania, psychosis)	Alteration causing no or minimal interference with usual social & functional activities	Alteration causing greater than minimal interference with usual social & functional activities	Alteration causing inability to perform usual social & functional activities	Behavior potentially harmful to self or others (e.g., suicidal and homicidal ideation or attempt, acute psychosis) OR Causing inability to perform basic self-care functions

PARAMETER	GRADE 1 MILD	GRADE 2 MODERATE	GRADE 3 SEVERE	GRADE 4 POTENTIALLY LIFE-THREATENING
Altered Mental Status<br><br>For Dementia, see Cognitive and behavioral/attentional disturbance (including dementia and attention deficit disorder)	Changes causing no or minimal interference with usual social & functional activities	Mild lethargy or somnolence causing greater than minimal interference with usual social & functional activities	Confusion, memory impairment, lethargy, or somnolence causing inability to perform usual social & functional activities	Delirium OR obtundation, OR coma
Ataxia	Asymptomatic ataxia detectable on exam OR Minimal ataxia causing no or minimal interference with usual social & functional activities	Symptomatic ataxia causing greater than minimal interference with usual social & functional activities	Symptomatic ataxia causing inability to perform usual social & functional activities	Disabling ataxia causing inability to perform basic self-care functions
Cognitive and behavioral/attentional disturbance (including dementia and attention deficit disorder)	Disability causing no or minimal interference with usual social & functional activities OR Specialized resources not indicated	Disability causing greater than minimal interference with usual social & functional activities OR Specialized resources on part-time basis indicated	Disability causing inability to perform usual social & functional activities OR Specialized resources on a full-time basis indicated	Disability causing inability to perform basic self-care functions OR Institutionalizati on indicated
CNS ischemia (acute)	NA	NA	Transient ischemic attack	Cerebral vascular accident (CVA, stroke) with neurological deficit

PARAMETER	GRADE 1<br>MILD	GRADE 2<br>MODERATE	GRADE 3<br>SEVERE	GRADE 4<br>POTENTIALLY LIFE-THREATENING
Developmental delay – **Pediatric ≤ 16 years**	Mild developmental delay, either motor or cognitive, as determined by comparison with a developmental screening tool appropriate for the setting	Moderate developmental delay, either motor or cognitive, as determined by comparison with a developmental screening tool appropriate for the setting	Severe developmental delay, either motor or cognitive, as determined by comparison with a developmental screening tool appropriate for the setting	Developmental regression, either motor or cognitive, as determined by comparison with a developmental screening tool appropriate for the setting
Headache	Symptoms causing no or minimal interference with usual social & functional activities	Symptoms causing greater than minimal interference with usual social & functional activities	Symptoms causing inability to perform usual social & functional activities	Symptoms causing inability to perform basic self-care functions OR Hospitalization indicated (other than emergency room visit) OR Headache with significant impairment of alertness or other neurologic function
Insomnia	NA	Difficulty sleeping causing greater than minimal interference with usual social & functional activities	Difficulty sleeping causing inability to perform usual social & functional activities	Disabling insomnia causing inability to perform basic self-care functions

PARAMETER	GRADE 1 MILD	GRADE 2 MODERATE	GRADE 3 SEVERE	GRADE 4 POTENTIALLY LIFE-THREATENING
Neuromuscular weakness (including myopathy & neuropathy)	Asymptomatic with decreased strength on exam OR Minimal muscle weakness causing no or minimal interference with usual social & functional activities	Muscle weakness causing greater than minimal interference with usual social & functional activities	Muscle weakness causing inability to perform usual social & functional activities	Disabling muscle weakness causing inability to perform basic self-care functions OR Respiratory muscle weakness impairing ventilation
Neurosensory alteration (including paresthesia and painful neuropathy)	Asymptomatic with sensory alteration on exam or minimal paresthesia causing no or minimal interference with usual social & functional activities	Sensory alteration or paresthesia causing greater than minimal interference with usual social & functional activities	Sensory alteration or paresthesia causing inability to perform usual social & functional activities	Disabling sensory alteration or paresthesia causing inability to perform basic self-care functions
Seizure: (new onset) **– Adult ≥ 18 years**<br><br>See also Seizure: (known pre-existing seizure disorder)	NA	1 seizure	2 – 4 seizures	Seizures of any kind which are prolonged, repetitive (e.g., status epilepticus), or difficult to control (e.g., refractory epilepsy)

PARAMETER	GRADE 1 MILD	GRADE 2 MODERATE	GRADE 3 SEVERE	GRADE 4 POTENTIALLY LIFE-THREATENING
Seizure: (known pre-existing seizure disorder) **– Adult ≥ 18 years**<br><br>For worsening of existing epilepsy the grades should be based on an increase from previous level of control to any of these levels.	NA	Increased frequency of pre-existing seizures (non-repetitive) without change in seizure character OR Infrequent break-through seizures while on stable medication in a previously controlled seizure disorder	Change in seizure character from baseline either in duration or quality (e.g., severity or focality)	Seizures of any kind which are prolonged, repetitive (e.g., status epilepticus), or difficult to control (e.g., refractory epilepsy)
Seizure **– Pediatric < 18 years**	Seizure, generalized onset with or without secondary generalization, lasting < 5 minutes with < 24 hours post ictal state	Seizure, generalized onset with or without secondary generalization, lasting 5 – 20 minutes with < 24 hours post ictal state	Seizure, generalized onset with or without secondary generalization, lasting > 20 minutes	Seizure, generalized onset with or without secondary generalization, requiring intubation and sedation
Syncope (not associated with a procedure)	NA	Present	NA	NA
Vertigo	Vertigo causing no or minimal interference with usual social & functional activities	Vertigo causing greater than minimal interference with usual social & functional activities	Vertigo causing inability to perform usual social & functional activities	Disabling vertigo causing inability to perform basic self-care functions

PARAMETER		GRADE 1 MILD	GRADE 2 MODERATE	GRADE 3 SEVERE	GRADE 4 POTENTIALLY LIFE-THREATENING
RESPIRATORY					
Bronchospasm (acute)		FEV1 or peak flow reduced to 70 – 80%	FEV1 or peak flow 50 – 69%	FEV1 or peak flow 25 – 49%	Cyanosis OR FEV1 or peak flow < 25% OR Intubation
Dyspnea or respiratory distress					
	**Adult ≥ 14 years**	Dyspnea on exertion with no or minimal interference with usual social & functional activities	Dyspnea on exertion causing greater than minimal interference with usual social & functional activities	Dyspnea at rest causing inability to perform usual social & functional activities	Respiratory failure with ventilatory support indicated
	**Pediatric < 14 years**	Wheezing OR minimal increase in respiratory rate for age	Nasal flaring OR Intercostal retractions OR Pulse oximetry 90 – 95%	Dyspnea at rest causing inability to perform usual social & functional activities OR Pulse oximetry < 90%	Respiratory failure with ventilatory support indicated
MUSCULOSKELETAL					
Arthralgia<br><br>See also Arthritis		Joint pain causing no or minimal interference with usual social & functional activities	Joint pain causing greater than minimal interference with usual social & functional activities	Joint pain causing inability to perform usual social & functional activities	Disabling joint pain causing inability to perform basic self-care functions

PARAMETER	GRADE 1 MILD	GRADE 2 MODERATE	GRADE 3 SEVERE	GRADE 4 POTENTIALLY LIFE-THREATENING
Arthritis<br><br>See also Arthralgia	Stiffness or joint swelling causing no or minimal interference with usual social & functional activities	Stiffness or joint swelling causing greater than minimal interference with usual social & functional activities	Stiffness or joint swelling causing inability to perform usual social & functional activities	Disabling joint stiffness or swelling causing inability to perform basic self-care functions
Bone Mineral Loss				
**Adult ≥ 21 years**	BMD t-score -2.5 to -1.0	BMD t-score < -2.5	Pathological fracture (including loss of vertebral height)	Pathologic fracture causing life-threatening consequences
**Pediatric < 21 years**	BMD z-score -2.5 to -1.0	BMD z-score < -2.5	Pathological fracture (including loss of vertebral height)	Pathologic fracture causing life-threatening consequences
Myalgia (non-injection site)	Muscle pain causing no or minimal interference with usual social & functional activities	Muscle pain causing greater than minimal interference with usual social & functional activities	Muscle pain causing inability to perform usual social & functional activities	Disabling muscle pain causing inability to perform basic self-care functions
Osteonecrosis	NA	Asymptomatic with radiographic findings AND No operative intervention indicated	Symptomatic bone pain with radiographic findings OR Operative intervention indicated	Disabling bone pain with radiographic findings causing inability to perform basic self-care functions

PARAMETER	GRADE 1 MILD	GRADE 2 MODERATE	GRADE 3 SEVERE	GRADE 4 POTENTIALLY LIFE-THREATENING
GENITOURINARY				
Cervicitis (<u>symptoms</u>)<br><br>(For use in studies evaluating topical study agents)<br><br>For other cervicitis see Infection: Infection (any other than HIV infection)	Symptoms causing no or minimal interference with usual social & functional activities	Symptoms causing greater than minimal interference with usual social & functional activities	Symptoms causing inability to perform usual social & functional activities	Symptoms causing inability to perform basic self-care functions
Cervicitis (<u>clinical exam</u>)<br><br>(For use in studies evaluating topical study agents)<br><br>For other cervicitis see Infection: Infection (any other than HIV infection)	Minimal cervical abnormalities on examination (erythema, mucopurulent discharge, or friability) OR Epithelial disruption < 25% of total surface	Moderate cervical abnormalities on examination (erythema, mucopurulent discharge, or friability) OR Epithelial disruption of 25 – 49% total surface	Severe cervical abnormalities on examination (erythema, mucopurulent discharge, or friability) OR Epithelial disruption 50 – 75% total surface	Epithelial disruption > 75% total surface
Inter-menstrual bleeding (IMB)	Spotting observed by participant OR Minimal blood observed during clinical or colposcopic examination	Inter-menstrual bleeding not greater in duration or amount than usual menstrual cycle	Inter-menstrual bleeding greater in duration or amount than usual menstrual cycle	Hemorrhage with life-threatening hypotension OR Operative intervention indicated
Urinary tract obstruction (e.g., stone)	NA	Signs or symptoms of urinary tract obstruction without hydronephrosis or renal dysfunction	Signs or symptoms of urinary tract obstruction with hydronephrosis or renal dysfunction	Obstruction causing life-threatening consequences

PARAMETER	GRADE 1 MILD	GRADE 2 MODERATE	GRADE 3 SEVERE	GRADE 4 POTENTIALLY LIFE-THREATENING
Vulvovaginitis (symptoms)<br><br>(Use in studies evaluating topical study agents)<br><br>For other vulvovaginitis see Infection: Infection (any other than HIV infection)	Symptoms causing no or minimal interference with usual social & functional activities	Symptoms causing greater than minimal interference with usual social & functional activities	Symptoms causing inability to perform usual social & functional activities	Symptoms causing inability to perform basic self-care functions
Vulvovaginitis (clinical exam)<br><br>(Use in studies evaluating topical study agents)<br><br>For other vulvovaginitis see Infection: Infection (any other than HIV infection)	Minimal vaginal abnormalities on examination OR Epithelial disruption < 25% of total surface	Moderate vaginal abnormalities on examination OR Epithelial disruption of 25 - 49% total surface	Severe vaginal abnormalities on examination OR Epithelial disruption 50 - 75% total surface	Vaginal perforation OR Epithelial disruption > 75% total surface
OCULAR/VISUAL				
Uveitis	Asymptomatic but detectable on exam	Symptomatic anterior uveitis OR Medical intervention indicated	Posterior or pan-uveitis OR Operative intervention indicated	Disabling visual loss in affected eye(s)
Visual changes (from baseline)	Visual changes causing no or minimal interference with usual social & functional activities	Visual changes causing greater than minimal interference with usual social & functional activities	Visual changes causing inability to perform usual social & functional activities	Disabling visual loss in affected eye(s)

PARAMETER	GRADE 1<br>MILD	GRADE 2<br>MODERATE	GRADE 3<br>SEVERE	GRADE 4<br>POTENTIALLY LIFE-THREATENING
ENDOCRINE/METABOLIC				
Abnormal fat accumulation (e.g., back of neck, breasts, abdomen)	Detectable by study participant (or by caregiver for young children and disabled adults)	Detectable on physical exam by health care provider	Disfiguring OR Obvious changes on casual visual inspection	NA
Diabetes mellitus	NA	New onset without need to initiate medication OR Modification of current medications to regain glucose control	New onset with initiation of medication indicated OR Diabetes uncontrolled despite treatment modification	Life-threatening consequences (e.g., ketoacidosis, hyperosmolar non-ketotic coma)
Gynecomastia	Detectable by study participant or caregiver (for young children and disabled adults)	Detectable on physical exam by health care provider	Disfiguring OR Obvious on casual visual inspection	NA
Hyperthyroidism	Asymptomatic	Symptomatic causing greater than minimal interference with usual social & functional activities OR Thyroid suppression therapy indicated	Symptoms causing inability to perform usual social & functional activities OR Uncontrolled despite treatment modification	Life-threatening consequences (e.g., thyroid storm)

PARAMETER	GRADE 1<br>MILD	GRADE 2<br>MODERATE	GRADE 3<br>SEVERE	GRADE 4<br>POTENTIALLY LIFE-THREATENING
Hypothyroidism	Asymptomatic	Symptomatic causing greater than minimal interference with usual social & functional activities OR Thyroid replacement therapy indicated	Symptoms causing inability to perform usual social & functional activities OR Uncontrolled despite treatment modification	Life-threatening consequences (e.g., myxedema coma)
Lipoatrophy (e.g., fat loss from the face, extremities, buttocks)	Detectable by study participant (or by caregiver for young children and disabled adults)	Detectable on physical exam by health care provider	Disfiguring OR Obvious on casual visual inspection	NA

Laboratory				
**PARAMETER**	**GRADE 1<br>MILD**	**GRADE 2<br>MODERATE**	**GRADE 3<br>SEVERE**	**GRADE 4<br>POTENTIALLY LIFE-THREATENING**
HEMATOLOGY	*Standard International Units are listed in italics*			
Absolute CD4+ count **– Adult and Pediatric > 13 years** (HIV NEGATIVE ONLY)	300 – 400/mm^3<br>*300 – 400/µL*	200 – 299/mm^3<br>*200 – 299/µL*	100 – 199/mm^3<br>*100 – 199/µL*	< 100/mm^3<br>*< 100/µL*
Absolute lymphocyte count **– Adult and Pediatric > 13 years** (HIV NEGATIVE ONLY)	600 – 650/mm^3<br>*0.600 x 10^9 – 0.650 x 10^9/L*	500 – 599/mm^3<br>*0.500 x 10^9 – 0.599 x 10^9/L*	350 – 499/mm^3<br>*0.350 x 10^9 – 0.499 x 10^9/L*	< 350/mm^3<br>*< 0.350 x 10^9/L*

**Laboratory**				
**PARAMETER**	**GRADE 1 MILD**	**GRADE 2 MODERATE**	**GRADE 3 SEVERE**	**GRADE 4 POTENTIALLY LIFE-THREATENING**
**Comment:** Values in children ≤ 13 years are not given for the two parameters above because the absolute counts are variable.				
Absolute neutrophil count (ANC)				
**Adult and Pediatric, > 7 days**	1,000 – 1,300/mm^3 *1.000 x 10^9 – 1.300 x 10^9/L*	750 – 999/mm^3 *0.750 x 10^9 – 0.999 x 10^9/L*	500 – 749/mm^3 *0.500 x 10^9 – 0.749 x 10^9/L*	< 500/mm^3 *< 0.500 x 10^9/L*
**Infant**[*]†, **2 – ≤ 7 days**	1,250 – 1,500/mm^3 *1.250 x 10^9 – 1.500 x 10^9/L*	1,000 – 1,249/mm^3 *1.000 x 10^9 – 1.249 x 10^9/L*	750 – 999/mm^3 *0.750 x 10^9 – 0.999 x 10^9/L*	< 750/mm^3 *< 0.750 x 10^9/L*
**Infant**[*]†, **≤1 day**	4,000 – 5,000/mm^3 *4.000 x 10^9 – 5.000 x 10^9/L*	3,000 – 3,999/mm^3 *3.000 x 10^9 – 3.999 x10^9/L*	1,500 – 2,999/mm^3 *1.500 x 10^9 – 2.999 x 10^9/L*	< 1,500/mm^3 *< 1.500 x 10^9/L*
**Comment:** Parameter changed from "Infant, < 1 day" to "Infant, **≤**1 day"				
Fibrinogen, decreased	100 – 200 mg/dL *1.00 – 2.00 g/L* OR 0.75 – 0.99 x LLN	75 – 99 mg/dL *0.75 – 0.99 g/L* OR 0.50 – 0.74 x LLN	50 – 74 mg/dL *0.50 – 0.74 g/L* OR 0.25 – 0.49 x LLN	< 50 mg/dL *< 0.50 g/L* OR < 0.25 x LLN OR Associated with gross bleeding
Hemoglobin (Hgb)				
**Comment:** The Hgb values in mmol/L have changed because the conversion factor used to convert g/dL to mmol/L has been changed from 0.155 to 0.6206 (the most commonly used conversion factor). For grading Hgb results obtained by an analytic method with a conversion factor other than 0.6206, the result must be converted to g/dL using the appropriate conversion factor for that lab.				
**Adult and Pediatric ≥ 57 days** (HIV POSITIVE ONLY)	8.5 – 10.0 g/dL *5.24 – 6.23 mmol/L*	7.5 – 8.4 g/dL *4.62–5.23 mmol/L*	6.50 – 7.4 g/dL *4.03–4.61 mmol/L*	< 6.5 g/dL *< 4.03 mmol/L*

Laboratory				
**PARAMETER**	**GRADE 1 MILD**	**GRADE 2 MODERATE**	**GRADE 3 SEVERE**	**GRADE 4 POTENTIALLY LIFE-THREATENING**
**Adult and Pediatric ≥ 57 days** (HIV NEGATIVE ONLY)	10.0 – 10.9 g/dL<br>*6.18* – 6.79 *mmol/L*<br><br>OR<br>Any decrease 2.5 – 3.4 g/dL<br>*1.58 – 2.13 mmol/L*	9.0 – 9.9 g/dL<br>*5.55 - 6.17 mmol/L*<br><br>OR<br>Any decrease 3.5 – 4.4 g/dL<br>*2.14 – 2.78 mmol/L*	7.0 – 8.9 g/dL<br>*4.34 - 5.54 mmol/L*<br><br>OR<br>Any decrease ≥ 4.5 g/dL<br>*> 2.79 mmol/L*	< 7.0 g/dL<br>*< 4.34 mmol/L*
**Comment:** The decrease is a decrease from baseline				
**Infant*†, 36 – 56 days** (HIV POSITIVE OR NEGATIVE)	8.5 – 9.4 g/dL<br>*5.24 – 5.86 mmol/L*	7.0 – 8.4 g/dL<br>*4.31 – 5.23 mmol/L*	6.0 – 6.9 g/dL<br>*3.72 – 4.30 mmol/L*	< 6.00 g/dL<br>*< 3.72 mmol/L*
**Infant*†, 22 – 35 days** (HIV POSITIVE OR NEGATIVE)	9.5 – 10.5 g/dL<br>*5.87 - 6.54 mmol/L*	8.0 – 9.4 g/dL<br>*4.93 – 5.86 mmol/L*	7.0 – 7.9 g/dL<br>*4.34 – 4.92 mmol/L*	< 7.00 g/dL<br>*< 4.34 mmol/L*
**Infant*†, ≤ 21 days** (HIV POSITIVE OR NEGATIVE)	12.0 – 13.0 g/dL<br>*7.42 – 8.09 mmol/L*	10.0 – 11.9 g/dL<br>*6.18 – 7.41 mmol/L*	9.0 – 9.9 g/dL<br>*5.59- 6.17 mmol/L*	< 9.0 g/dL<br>*< 5.59 mmol/L*
**Correction**: Parameter changed from "Infant < 21 days" to "Infant ≤ 21 days"				
International Normalized Ratio of prothrombin time (INR)	1.1 – 1.5 x ULN	1.6 – 2.0 x ULN	2.1 – 3.0 x ULN	> 3.0 x ULN
Methemoglobin	5.0 – 10.0%	10.1 – 15.0%	15.1 – 20.0%	> 20.0%
Prothrombin Time (PT)	1.1 – 1.25 x ULN	1.26 – 1.50 x ULN	1.51 – 3.00 x ULN	> 3.00 x ULN
Partial Thromboplastin Time (PTT)	1.1 – 1.66 x ULN	1.67 – 2.33 x ULN	2.34 – 3.00 x ULN	> 3.00 x ULN

Laboratory				
**PARAMETER**	**GRADE 1 MILD**	**GRADE 2 MODERATE**	**GRADE 3 SEVERE**	**GRADE 4 POTENTIALLY LIFE-THREATENING**
Platelets, decreased	100,000 – 124,999/mm^3 *100.000 x 10^9 – 124.999 x 10^9/L*	50,000 – 99,999/mm^3 *50.000 x 10^9 – 99.999 x 10^9/L*	25,000 – 49,999/mm^3 *25.000 x 10^9 – 49.999 x 10^9/L*	< 25,000/mm^3 *< 25.000 x 10^9/L*
WBC, decreased	2,000 – 2,500/mm^3 *2.000 x 10^9 – 2.500 x 10^9/L*	1,500 – 1,999/mm^3 *1.500 x 10^9 – 1.999 x 10^9/L*	1,000 – 1,499/mm^3 *1.000 x 10^9 – 1.499 x 10^9/L*	< 1,000/mm^3 *< 1.000 x 10^9/L*
CHEMISTRIES	*Standard International Units are listed in italics*			
Acidosis	NA	pH < normal, but ≥ 7.3	pH < 7.3 without life-threatening consequences	pH < 7.3 with life-threatening consequences
Albumin, serum, low	3.0 g/dL – < LLN *30 g/L – < LLN*	2.0 – 2.9 g/dL *20 – 29 g/L*	< 2.0 g/dL *< 20 g/L*	NA
Alkaline Phosphatase	1.25 – 2.5 x ULN†	2.6 – 5.0 x ULN†	5.1 – 10.0 x ULN†	> 10.0 x ULN†
Alkalosis	NA	pH > normal, but ≤ 7.5	pH > 7.5 without life-threatening consequences	pH > 7.5 with life-threatening consequences
ALT (SGPT)	1.25 – 2.5 x ULN	2.6 – 5.0 x ULN	5.1 – 10.0 x ULN	> 10.0 x ULN
AST (SGOT)	1.25 – 2.5 x ULN	2.6 – 5.0 x ULN	5.1 – 10.0 x ULN	> 10.0 x ULN
Bicarbonate, serum, low	16.0 mEq/L – < LLN *16.0 mmol/L – < LLN*	11.0 – 15.9 mEq/L *11.0 – 15.9 mmol/L*	8.0 – 10.9 mEq/L *8.0 – 10.9 mmol/L*	< 8.0 mEq/L *< 8.0 mmol/L*
**Comment:** Some laboratories will report this value as Bicarbonate (HCO_3) and others as Total Carbon Dioxide (CO_2). These are the same tests; values should be graded according to the ranges for Bicarbonate as listed above.				

Laboratory				
**PARAMETER**	**GRADE 1 MILD**	**GRADE 2 MODERATE**	**GRADE 3 SEVERE**	**GRADE 4 POTENTIALLY LIFE-THREATENING**
Bilirubin (Total)				
**Adult and Pediatric > 14 days**	1.1 – 1.5 x ULN	1.6 – 2.5 x ULN	2.6 – 5.0 x ULN	> 5.0 x ULN
**Infant*†, ≤ 14 days** (non-hemolytic)	NA	20.0 – 25.0 mg/dL *342 – 428 µmol/L*	25.1 – 30.0 mg/dL *429 – 513 µmol/L*	> 30.0 mg/dL *> 513.0 µmol/L*
**Infant*†, ≤ 14 days** (hemolytic)	NA	NA	20.0 – 25.0 mg/dL *342 – 428 µmol/L*	> 25.0 mg/dL *> 428 µmol/L*
Calcium, serum, high				
**Adult and Pediatric ≥ 7 days**	10.6 – 11.5 mg/dL *2.65 – 2.88 mmol/L*	11.6 – 12.5 mg/dL *2.89 – 3.13 mmol/L*	12.6 – 13.5 mg/dL *3.14 – 3.38 mmol/L*	> 13.5 mg/dL *> 3.38 mmol/L*
**Infant*†, < 7 days**	11.5 – 12.4 mg/dL *2.88 – 3.10 mmol/L*	12.5 – 12.9 mg/dL *3.11 – 3.23 mmol/L*	13.0 – 13.5 mg/dL *3.245 – 3.38 mmol/L*	> 13.5 mg/dL *> 3.38 mmol/L*
Calcium, serum, low				
**Adult and Pediatric ≥ 7 days**	7.8 – 8.4 mg/dL *1.95 – 2.10 mmol/L*	7.0 – 7.7 mg/dL *1.75 – 1.94 mmol/L*	6.1 – 6.9 mg/dL *1.53 – 1.74 mmol/L*	< 6.1 mg/dL *< 1.53 mmol/L*
**Infant*†, < 7 days**	6.5 – 7.5 mg/dL *1.63 – 1.88 mmol/L*	6.0 – 6.4 mg/dL *1.50 – 1.62 mmol/L*	5.50 – 5.90 mg/dL *1.38 – 1.51 mmol/L*	< 5.50 mg/dL *< 1.38 mmol/L*
**Comment:** Do not adjust Calcium, serum, low or Calcium, serum, high for albumin				

Laboratory					
**PARAMETER**		**GRADE 1 MILD**	**GRADE 2 MODERATE**	**GRADE 3 SEVERE**	**GRADE 4 POTENTIALLY LIFE-THREATENING**
Cardiac troponin I (cTnI)		NA	NA	NA	Levels consistent with myocardial infarction or unstable angina as defined by the manufacturer
Cardiac troponin T (cTnT)		NA	NA	NA	≥ 0.20 ng/mL OR Levels consistent with myocardial infarction or unstable angina as defined by the manufacturer
Cholesterol (fasting)					
	**Adult ≥ 18 years**	200 – 239 mg/dL *5.18 – 6.19 mmol/L*	240 – 300 mg/dL *6.20 – 7.77 mmol/L*	> 300 mg/dL *> 7.77 mmol/L*	NA
	**Pediatric < 18 years**	170 – 199 mg/dL *4.40 – 5.15 mmol/L*	200 – 300 mg/dL *5.16 – 7.77 mmol/L*	> 300 mg/dL *> 7.77 mmol/L*	NA
Creatine Kinase		3.0 – 5.9 x ULN†	6.0 – 9.9 x ULN†	10.0 – 19.9 x ULN†	≥ 20.0 x ULN†
Creatinine		1.1 – 1.3 x ULN†	1.4 – 1.8 x ULN†	1.9 – 3.4 x ULN†	≥ 3.5 x ULN†
Glucose, serum, high					
	Nonfasting	116 – 160 mg/dL *6.44 – 8.88 mmol/L*	161 – 250 mg/dL *8.89 – 13.88 mmol/L*	251 – 500 mg/dL *13.89 – 27.75 mmol/L*	> 500 mg/dL *> 27.75 mmol/L*
	Fasting	110 – 125 mg/dL *6.11 – 6.94 mmol/L*	126 – 250 mg/dL *6.95 – 13.88 mmol/L*	251 – 500 mg/dL *13.89 – 27.75 mmol/L*	> 500 mg/dL *> 27.75 mmol/L*
Glucose, serum, low					

Laboratory				
**PARAMETER**	**GRADE 1 MILD**	**GRADE 2 MODERATE**	**GRADE 3 SEVERE**	**GRADE 4 POTENTIALLY LIFE-THREATENING**
**Adult and Pediatric ≥ 1 month**	55 – 64 mg/dL *3.05 – 3.55 mmol/L*	40 – 54 mg/dL *2.22 – 3.06 mmol/L*	30 – 39 mg/dL *1.67 – 2.23 mmol/L*	< 30 mg/dL *< 1.67 mmol/L*
**Infant[*†], < 1 month**	50 – 54 mg/dL *2.78 – 3.00 mmol/L*	40 – 49 mg/dL *2.22 – 2.77 mmol/L*	30 – 39 mg/dL *1.67 – 2.21 mmol/L*	< 30 mg/dL *< 1.67 mmol/L*
Lactate	ULN - < 2.0 x ULN without acidosis	≥ 2.0 x ULN without acidosis	Increased lactate with pH < 7.3 without life-threatening consequences	Increased lactate with pH < 7.3 with life-threatening consequences
**Comment:** Added ULN to Grade 1 parameter				
LDL cholesterol (fasting)				
**Adult ≥ 18 years**	130 – 159 mg/dL *3.37 – 4.12 mmol/L*	160 – 190 mg/dL *4.13 – 4.90 mmol/L*	≥ 190 mg/dL *≥4.91 mmol/L*	NA
**Pediatric > 2 - < 18 years**	110 – 129 mg/dL *2.85 – 3.34 mmol/L*	130 – 189 mg/dL *3.35 – 4.90 mmol/L*	≥ 190 mg/dL *≥ 4.91 mmol/L*	NA
Lipase	1.1 – 1.5 x ULN	1.6 – 3.0 x ULN	3.1 – 5.0 x ULN	> 5.0 x ULN
Magnesium, serum, low	1.2 – 1.4 mEq/L *0.60 – 0.70 mmol/L*	0.9 – 1.1 mEq/L *0.45 – 0.59 mmol/L*	0.6 – 0.8 mEq/L *0.30 – 0.44 mmol/L*	< 0.60 mEq/L *< 0.30 mmol/L*
Pancreatic amylase	1.1 – 1.5 x ULN	1.6 – 2.0 x ULN	2.1 – 5.0 x ULN	> 5.0 x ULN
Phosphate, serum, low				
**Adult and Pediatric > 14 years**	2.5 mg/dL – < LLN *0.81 mmol/L – < LLN*	2.0 – 2.4 mg/dL *0.65 – 0.80 mmol/L*	1.0 – 1.9 mg/dL *0.32 – 0.64 mmol/L*	< 1.00 mg/dL *< 0.32 mmol/L*

Laboratory				
**PARAMETER**	**GRADE 1 MILD**	**GRADE 2 MODERATE**	**GRADE 3 SEVERE**	**GRADE 4 POTENTIALLY LIFE-THREATENING**
**Pediatric 1 year – 14 years**	3.0 – 3.5 mg/dL<br>*0.97 – 1.13 mmol/L*	2.5 – 2.9 mg/dL<br>*0.81 – 0.96 mmol/L*	1.5 – 2.4 mg/dL<br>*0.48 – 0.80 mmol/L*	< 1.50 mg/dL<br>*< 0.48 mmol/L*
**Pediatric < 1 year**	3.5 – 4.5 mg/dL<br>*1.13 – 1.45 mmol/L*	2.5 – 3.4 mg/dL<br>*0.81 – 1.12 mmol/L*	1.5 – 2.4 mg/dL<br>*0.48 – 0.80 mmol/L*	< 1.50 mg/dL<br>*< 0.48 mmol/L*
Potassium, serum, high	5.6 – 6.0 mEq/L<br>*5.6 – 6.0 mmol/L*	6.1 – 6.5 mEq/L<br>*6.1 – 6.5 mmol/L*	6.6 – 7.0 mEq/L<br>*6.6 – 7.0 mmol/L*	> 7.0 mEq/L<br>*> 7.0 mmol/L*
Potassium, serum, low	3.0 – 3.4 mEq/L<br>*3.0 – 3.4 mmol/L*	2.5 – 2.9 mEq/L<br>*2.5 – 2.9 mmol/L*	2.0 – 2.4 mEq/L<br>*2.0 – 2.4 mmol/L*	< 2.0 mEq/L<br>*< 2.0 mmol/L*
Sodium, serum, high	146 – 150 mEq/L<br>*146 – 150 mmol/L*	151 – 154 mEq/L<br>*151 – 154 mmol/L*	155 – 159 mEq/L<br>*155 – 159 mmol/L*	≥ 160 mEq/L<br>*≥ 160 mmol/L*
Sodium, serum, low	130 – 135 mEq/L<br>*130 – 135 mmol/L*	125 – 129 mEq/L<br>*125 – 129 mmol/L*	121 – 124 mEq/L<br>*121 – 124 mmol/L*	≤ 120 mEq/L<br>*≤ 120 mmol/L*
Triglycerides (fasting)	NA	500 – 750 mg/dL<br>*5.65 – 8.48 mmol/L*	751 – 1,200 mg/dL<br>*8.49 – 13.56 mmol/L*	> 1,200 mg/dL<br>*> 13.56 mmol/L*
Uric acid	7.5 – 10.0 mg/dL<br>*0.45 – 0.59 mmol/L*	10.1 – 12.0 mg/dL<br>*0.60 – 0.71 mmol/L*	12.1 – 15.0 mg/dL<br>*0.72 – 0.89 mmol/L*	> 15.0 mg/dL<br>*> 0.89 mmol/L*
URINALYSIS	*Standard International Units are listed in italics*			
Hematuria (microscopic)	6 – 10 RBC/HPF	> 10 RBC/HPF	Gross, with or without clots OR with RBC casts	Transfusion indicated

Laboratory				
**PARAMETER**	**GRADE 1 MILD**	**GRADE 2 MODERATE**	**GRADE 3 SEVERE**	**GRADE 4 POTENTIALLY LIFE-THREATENING**
Proteinuria, random collection	1 +	2 – 3 +	4 +	NA
Proteinuria, 24 hour collection				
**Adult and Pediatric ≥ 10 years**	200 – 999 mg/24 h *0.200 – 0.999 g/d*	1,000 – 1,999 mg/24 h *1.000 – 1.999 g/d*	2,000 – 3,500 mg/24 h *2.000 – 3.500 g/d*	> 3,500 mg/24 h *> 3.500 g/d*
**Pediatric > 3 mo - < 10 years**	201 – 499 $mg/m^2/24$ h *0.201 – 0.499 g/d*	500 – 799 $mg/m^2/24$ h *0.500 – 0.799 g/d*	800 – 1,000 $mg/m^2/24$ h *0.800 – 1.000 g/d*	> 1,000 mg/ $m^2/24$ h *> 1.000 g/d*

11.4. Appendix 4: Pharmacogenetics

Pharmacogenetics – Background

Pharmacogenetics (PGx) is the study of variability in drug response due to hereditary factors in different populations. There is increasing evidence that an individual's genetic composition (i.e., genotype) may impact the pharmacokinetics (absorption, distribution, metabolism, excretion), pharmacodynamics (relationship between concentrations and pharmacologic effects or the time course of pharmacologic effects) and/or clinical outcome (in terms of efficacy and/or safety and tolerability). Some reported examples of PGx analysis include:

Drug	Disease	Gene	Outcome
Abacavir	HIV [Hetherington, 2002; Mallal, 2008]	*HLA-B* (Human Leukocyte Antigen B)	Carriage of the *HLA-B*5701* variant has been shown to increase a patient's risk for experiencing hypersensitivity to abacavir. Prospective *HLA-B*5701* screening and exclusion of *HLA-B*5701* positive patients from abacavir treatment significantly decreased the incidence of abacavir hypersensitivity. Treatment guidelines and abacavir product labeling in the United States and Europe now recommend (US) or require (EU) prospective *HLA-B*5701* screening prior to initiation of abacavir to reduce the incidence of abacavir hypersensitivity. *HLA-B*5701* screening should supplement but must never replace clinical risk management strategies for abacavir hypersensitivity.
Warfarin	Cardiovascular [The International Warfarin Pharmacogenetics Consortium, 2009]	*CYP2C9* & *VKORC1* (Vitamin K epoxide reductase complex subunit 1)	In a retrospective study in over 5,000 patients who initiated warfarin therapy, an algorithm that included both clinical and genetic factors was better correlated with the empirically determined stable warfarin maintenance dose for outliers (patients who did not respond to a standard 5 mg dose) than an algorithm that only included clinical factors.

A key component to successful PGx research is the collection of samples during the conduct of clinical studies.

Collection of whole blood samples, even when no a priory hypothesis has been identified, may enable PGx analysis to be conducted if at any time it appears that there is a potential unexpected or unexplained variation in handling or response to any of the HIV medicines taken in this study.

Pharmacogenetic Research Objectives

The objective of the PGx research (if there is a potential unexpected or unexplained variation) is to investigate a possible genetic relationship to handling or response to any of the HIV medicines used in this study. If at any time it appears there is potential variability in response in this clinical study or in a series of clinical studies with HIV medicines taken in this study that may be attributable to genetic variation of subjects, the following objectives may be investigated:

- Relationship between genetic variants and the pharmacokinetics and/or pharmacodynamics of HIV medicines taken in this study
- Relationship between genetic variants and safety and/or tolerability of HIV medicines taken in this study
- Relationship between genetic variants and efficacy of HIV medicines taken in this study

Study Population

Any subject who has given informed consent to participate in the clinical study, has met all the entry criteria for the clinical study, and receives treatment for HIV infection may take part in the PGx research. Any subject who has received an allogeneic bone marrow transplant must be excluded from the PGx research.

Subject participation in the PGx research is voluntary and refusal to participate will not indicate withdrawal from the clinical study. Refusal to participate will involve no penalty or loss of benefits to which the subject would otherwise be entitled.

Study Assessments and Procedures

In addition to any blood samples take for the clinical study, a whole blood sample (~10ml) will be collected for the PGx research using a tube containing EDTA. The PGx sample is labeled (or “coded”) with a study specific number that can be traced or linked back to the subject by the Investigator or site staff. Coded samples do not carry personal identifiers (such as name or social security number). The blood sample will be taken on a single occasion unless a duplicate sample is required due to inability to utilize the original sample. It is recommended that the blood sample be taken at the first opportunity after a subject has been randomized and provided informed consent for PGx research, but may be taken at any time while the subject is participating in the clinical study.

If deoxyribonucleic acid (DNA) is extracted from the blood sample, the DNA may be subjected to sample quality control analysis. This analysis will involve the genotyping of several genetic markers to confirm the integrity of individual samples. If inconsistencies are noted in the analysis, then those samples may be destroyed.

The need to conduct PGx analysis may be identified after a study (or a set of studies) of the HIV therapies taken in this study has been completed and the study data reviewed.

In some cases, the samples may not be studied. e.g., no questions are raised about how people respond to the HIV therapies taken in this study.

Samples will be stored securely and may be kept for up to 15 years after the last subject completes the study or the Sponsor may destroy the samples sooner. The Sponsor or those working with the Sponsor (for example, other researchers) will use samples collected from the study for the purpose stated in this protocol and in the informed consent form.

Subjects can request their sample to be destroyed at any time.

Subject Withdrawal from Study

If a subject who has consented to participate in PGx research and has a sample taken for PGx research withdraws from the clinical study for any reason other than lost to follow-up, the subject will be given the following options:

- The sample is retained for PGx research
- Any PGx sample is destroyed.

If a subject withdraws consent from the PGx research or requests sample destruction for any reason, the Investigator must complete the appropriate documentation to request sample destruction within the timeframe specified by the Sponsor and maintain the documentation in the site study records. In either case, the Sponsor will only keep study information collected/generated up to that point.

Screen and Baseline Failures

If a blood sample for PGx research has been collected and it is determined that the subject does not meet the entry criteria for participation in the clinical study, then the Investigator must complete the appropriate documentation to request sample destruction within the timeframe specified by the Sponsor and maintain the documentation in the site study records.

Pharmacogenetics Analyses

Generally the Sponsor will utilize two approaches to explore genetic variation in drug response.

1. Specific sections of DNA may be selected from areas of the genome (e.g., candidate genes) known to encode the drug target, drug metabolizing enzymes, areas associated with mechanisms underlying adverse events, and those linked to study disease and, thus, linked to drug response.

In addition, continuing research may identify other enzymes, transporters, proteins, or receptors that may be involved in response to the HIV medicines taken in this study. The genes that may code for these proteins may also be studied.

2. Genome-wide scans involving a large numbers of polymorphic markers (e.g., single nucleotide polymorphisms or SNPs) located throughout the genome. This approach is often employed when potential genetic effects are not well understood.

Typically the methods used to identify markers that are associated with drug response are:

Hardy-Weinberg Equilibrium Testing

The genotypic frequencies of each polymorphism will be evaluated for conformity to those expected under normal conditions by employing Hardy-Weinberg Equilibrium testing.

Comparison of Demographic and Baseline Characteristics by Genotype

Differences in Baseline clinical characteristics and potential contributing covariates may be summarized and compared among genotype (or haplotype) subgroups.

Evaluation of Genotypic Effects

Analyses may be carried out to evaluate the degree of association between subject genotype (or haplotype) and selected parameters (e.g., pharmacokinetics, efficacy and safety). Where such genotypic tests are inappropriate (for example, where the number of marker genotypes is too large and/or the frequency of individual genotypes too small), allelic tests may be conducted. Allelic tests evaluate whether the frequency of each marker allele is the same in responders and non-responders.

Evaluation of Treatment by Genotype and Gene-Gene Interaction

In addition to evaluating the main effects of the genotypes (haplotypes or alleles) on the selected parameters, the possibility of a treatment group by genotype (haplotype or allele) interaction will also be explored. If appropriate, the joint effects of multiple markers (gene-gene interactions) may also be evaluated.

Linkage Disequilibrium

For pairs of polymorphisms, the degree to which alleles from the two sites are correlated (linkage disequilibrium) may also be evaluated. If the genotypes at two polymorphic sites within a gene are shown to be statistically associated with a response to IP, the degree of linkage disequilibrium will aid interpretation in that it will indicate the extent to which the two sites are exerting independent effects.

Multiple Comparisons and Multiplicity

An adjustment to observed p-values may be made to limit erroneous conclusions due to multiple tests when multiple markers are evaluated (especially in the case of a genome scan for association),

Power and Sample Size Considerations

The ability to detect differential drug response among genotypes at a polymorphic site depends on the total number of subjects genotyped and the frequency distribution of the different genotypes. Consequently, genotyping analyses are plausible for those polymorphic sites where the number of subjects comprising the genotypic groups is sufficiently large; however, these frequencies will not be known until sufficient samples have been collected and genotyping is complete.

Estimates of sample sizes required to demonstrate genotype effects vary considerably, depending on the assumptions made about allele frequency, genetic effect size, and mechanism of inheritance [Cardon, 2000]. In the work by Palmer and Cookson [Palmer, 2001], which assumed a genotype relative risk of 1.5, it was estimated that more than 300 cases and 600 controls would be needed to conduct a genetic association analysis. In contrast, McCarthy and Hilfiker [McCarthy, 2000] showed that with a genotype relative risk of 2.16 and a relatively commonly occurring genotype, only 30 cases and 30 controls would be needed to demonstrate an association.

Published PGx examples include abacavir hypersensitivity reaction [Hetherington, 2002; Mallal, 2008] and tranilast induced hyperbilirubinemia [Roses, 2002] where genetic markers have been found to significantly associate with hypersensitivity reaction (abacavir) and hyperbilirubinemia (tranilast). These examples show that small sample sizes typically encountered in Phase I and Phase II studies may be sufficient to identify clinically relevant genetic associations.

Informed Consent

Subjects who do not wish to participate in the PGx research may still participate in the clinical study. PGx informed consent must be obtained prior to any blood being taken for PGx research.

Provision of Study Results and Confidentiality of Subject's PGx Data

The Sponsor may summarize the cumulative PGx research results in the clinical study report.

In general, the Sponsor does not inform the Investigator, subject, or anyone else (e.g., family members, study Investigators, primary care physicians, insurers, or employers) of the PGx research results that are not known to be relevant to the subject's medical care at the time of the study, because the information generated from PGx studies is preliminary in nature, and the significance and scientific validity of the results are undetermined at such an early stage of research, under any circumstances unless required by law.

References

Cardon LR, Idury RM, Harris TJR, Witte JS, Elston RC. Testing drug response in the presence of genetic information: sampling issues for clinical trials. *Pharmacogenetics* 2000; 10:503-10.

Hetherington S, Hughes AR, Mosteller M, Shortino D, Baker KL, Spreen W, Lai E, Davies K, Handley A, Dow DJ, Fling ME, Stocum M, Bowman C, Thurmond LM, Roses AD. Genetic variations in HLA-B region and hypersensitivity reactions to abacavir. *Lancet.* 2002; 359:1121-2.

Mallal S, Phillips E, Giampiero C, Molina J-M, Workman C, Tomzic J, Jagel-Guedes E, Rugina S, Kozyrev O, Flores Cid J, Hay P, Nolan D, Hughes S, Hughes A, Ryan S, Fitch N, Thorborn D, Benbow A. *HLA-B*5701* Screening for hypersensitivity to abacavir. N Engl J Med. 2008, 358: 568-79.

McCarthy JJ, Hilfiker R. The use of single-nucleotide polymorphism maps in pharmacogenomics. *Nat Biotechnol* 2000; 18:505-8.

Palmer LJ, Cookson WO. Using single nucleotide polymorphisms as a means to understanding the pathophysiology of asthma. Respir Res 2001; 2:102-12.

Roses AD. Genome-based pharmacogenetics and the pharmaceutical industry. Nat Rev Drug Discov 2002; 1:541-9.

The International Warfarin Pharmacogenetics Consortium. Estimation of the warfarin dose with clinical and pharmacogenetic data. N Engl J Med 2009; 360: 753-64.

11.5. Appendix 5: Protocol Changes

Changes

[redacted]

[redacted]

[redacted]

[redacted]

Section 1.1 (Background) previously read

There are over twenty Food and Drug Administration (FDA) approved antiretroviral agents available with which to construct a highly active antiretroviral therapy (HAART) regimen which has proven to reduce morbidity and mortality in individuals with human immunodeficiency virus infection (HIV) or acquired immunodeficiency syndrome (AIDS) [Palella, 1998]. The current standard of therapy is a combination of at least three antiretrovirals consisting of two nucleoside/nucleotide (NRTI) analogues plus either a non-nucleoside reverse transcriptase inhibitor (NNRTI), a protease inhibitor (PI), or an integrase inhibitor [DHHS, 2009]. Among the available agents, atazanavir (ATV) boosted with ritonavir (RTV) + tenofovir/emtricitabine (TDF/FTC) is currently a preferred initial regimen for antiretroviral-naïve patients to reduce viral load and improve CD4 lymphocyte counts.

Low-dose RTV is commonly added to PI-based regimens to provide pharmacokinetic enhancement of the parent PI and to reduce the risk of drug resistance. However, RTV is also associated with adverse effects including gastrointestinal upset and lipid and metabolic alterations that may increase future cardiovascular risk. In addition, RTV is a perpetrator of cytochrome P450-mediated drug interactions which can limit the use of other concomitant drugs [Norvir, Package Insert, 2009; Reyataz, Package Insert, 2009]. The addition of RTV to PIs not co-formulated with RTV adds to the patient's pill burden and is associated with extra medication costs. Therefore, there is continued interest among clinicians to find alternative treatment strategies that do not require the use of RTV but continue to offer the potency and high virologic resistance barrier of protease inhibitors.

Treatment induction with a highly potent combination regimen followed by simplification is one strategy that has demonstrated comparable safety and efficacy outcomes in several studies [Gatell, 2007; Delfraissy, 2008; Squires, 2009]. Regimen induction with a RTV-boosted PI provides rapid initial virologic suppression reducing risk for development of viral resistance while subsequent simplification optimizes tolerability and adherence, minimizes short and long-term toxicity, and may reduce drug-drug interactions.

The SWAN study was a multicenter, randomized, open-label trial of 419 patients on a stable, virologically suppressive, PI-based regimen for a mean of 40.3 months who were randomized 2:1 to either switch to an ATV-containing regimen with unchanged NRTIs or remain on the initial non-ATV PI-based regimen. ATV was administered without RTV except in approximately 9% of subjects whose NRTIs included TDF, in which case ATV was given with low-dose RTV due to the pharmacokinetic interaction of ATV and TDF which requires that when using ATV in combination with TDF it must be boosted by RTV [Gatell, 2007]. In a post-hoc analysis among 153 subjects who were initially stable on a virologically suppressive, Lopinavir (LPV)/RTV based regimen, 82 (53%) switched to an unboosted ATV-based regimen, 18 (12%) switched to an ATV/RTV based regimen, and the remaining 53 subjects (34.5%) continued on the original LPV/RTV based regimen. At Week 48, the rates of viral rebound were comparable; 11% in the combined ATV arm and 9% in the LPV/RTV arm [Gatell, 2006]. Rates of treatment failure for any reason, which included viral rebound, failure to receive the randomized treatment, or discontinuation of study therapy were similar in both groups (26% of patients on LPV/RTV versus 28% on ATV). In addition, subjects switched to ATV had a reduction in new onset gastrointestinal symptoms, improvements in lipid parameters, and a lower usage of lipid lowering agents compared to subjects remaining on the LPV/RTV arm [Gatell, 2006]. This post-hoc analysis demonstrated that subjects switching from a stable, virologically suppressive, LPV/RTV based regimen to a RTV-boosted or RTV-sparing ATV based regimen maintained similar virologic control with fewer lipid abnormalities and better gastrointestinal tolerability.

The ARIES study was a 144-week, phase IIIB, randomized, open-label, multi-center study in 419 randomized patients who had achieved virologic suppression during the initial 36-week induction phase. In the induction phase, patients received ATV/RTV + abacavir sulfate/lamivudine (ABC/3TC) over 36 weeks and those achieving virologic suppression were subsequently randomized (1:1) to a simplification regimen of ATV + ABC/3TC or remained on the induction regimen and then followed for 108 weeks. At week 84 (48 weeks from the start of randomization), 86% of patients randomized to the simplification arm vs. 81% of patients remaining on the induction arm maintained virologic suppression to HIV-1 RNA <50 copies/mL (c/mL) [Squires, 2009]. Virologic failure occurred in 0.5% of patients in the simplification arm compared to 3% of patients in the induction arm through 84 weeks. Results were similar among patients with entry viral loads above and below 100,000 c/mL. Patients on the simplification regimen had less hyperbilirubinemia and a reduction in total cholesterol and triglycerides.

ABC/3TC is a dual nucleoside combination that is also commonly used with RTV-boosted ATV, but may also be used with unboosted ATV in appropriate patient populations. Recent data have shown that the combination of unboosted ATV plus ABC/3TC effectively maintains virologic suppression (plasma HIV-1 RNA <50 c/mL) after initial induction with RTV-boosting of ATV in HIV-infected patients [Delfraissy, 2008; Squires, 2009]. In addition, ABC/3TC has a well-established safety profile and relatively few drug interactions [EPZICOM® Package Insert, 2009].

The current study will evaluate whether subjects receiving a RTV-containing regimen of ATV/RTV + TDF/FTC who have achieved virologic suppression can safely simplify to a

RTV-sparing regimen of ATV + ABC/3TC and maintain virologic suppression through 48 weeks.

This study will also evaluate changes in neurocognition (via a computerized test battery), and cardiovascular, renal and bone biomarkers as exploratory endpoints. As the life expectancy of HIV-1 infected patients continues to improve, long-term consequences of chronic HIV-infection and potentially specific antiretroviral therapies is of active research interest. Additionally, this study will increase our knowledge regarding the incidence of clinically suspected abacavir hypersensitivity reaction (ABC HSR) after excluding patients who carry the HLA-B*5701 allele.

And now reads:

There are over twenty Food and Drug Administration (FDA) approved antiretroviral agents available with which to construct a highly active antiretroviral therapy (HAART) regimen which has proven to reduce morbidity and mortality in individuals with human immunodeficiency virus infection (HIV) or acquired immunodeficiency syndrome (AIDS) [Palella, 1998]. The current standard of therapy is a combination of at least three antiretrovirals consisting of two nucleoside/nucleotide (NRTI) analogues plus either a non-nucleoside reverse transcriptase inhibitor (NNRTI), a protease inhibitor (PI), or an integrase inhibitor [DHHS, 2009]. Among the available agents, atazanavir (ATV) boosted with ritonavir (RTV) + tenofovir/emtricitabine (TDF/FTC) is currently a preferred initial regimen for antiretroviral-naïve patients to reduce viral load and improve CD4 lymphocyte counts.

Low-dose RTV is commonly added to PI-based regimens to provide pharmacokinetic enhancement of the parent PI and to reduce the risk of drug resistance. However, RTV is also associated with adverse effects including gastrointestinal upset and lipid and metabolic alterations that may increase future cardiovascular risk. In addition, RTV is a perpetrator of cytochrome P450-mediated drug interactions which can limit the use of other concomitant drugs [Norvir, Package Insert, 2010; Reyataz, Package Insert, 2010]. The addition of RTV to PIs not co-formulated with RTV adds to the patient’s pill burden and is associated with extra medication costs. Therefore, there is continued interest among clinicians to find alternative treatment strategies that do not require the use of RTV but continue to offer the potency and high virologic resistance barrier of protease inhibitors.

Treatment induction with a highly potent combination regimen followed by simplification is one strategy that has demonstrated comparable safety and efficacy outcomes in several studies [Gatell, 2007; Delfraissy, 2008; Squires, 2010a; Squires, 2010b]. Regimen induction with a RTV-boosted PI provides rapid initial virologic suppression reducing risk for development of viral resistance while subsequent simplification optimizes tolerability and adherence, minimizes short and long-term toxicity, and may reduce drug-drug interactions.

The SWAN study was a multicenter, randomized, open-label trial of 419 patients on a stable, virologically suppressive, PI-based regimen for a mean of 40.3 months who were randomized 2:1 to either switch to an ATV-containing regimen with unchanged NRTIs or remain on the initial non-ATV PI-based regimen. ATV was administered without RTV

except in approximately 9% of subjects whose NRTIs included TDF, in which case ATV was given with low-dose RTV due to the pharmacokinetic interaction of ATV and TDF which requires that when using ATV in combination with TDF it must be boosted by RTV [Gatell, 2007]. In a post-hoc analysis among 153 subjects who were initially stable on a virologically suppressive, Lopinavir (LPV)/RTV based regimen, 82 (53%) switched to an unboosted ATV-based regimen, 18 (12%) switched to an ATV/RTV based regimen, and the remaining 53 subjects (34.5%) continued on the original LPV/RTV based regimen. At Week 48, the rates of viral rebound were comparable; 11% in the combined ATV arm and 9% in the LPV/RTV arm [Gatell, 2006]. Rates of treatment failure for any reason, which included viral rebound, failure to receive the randomized treatment, or discontinuation of study therapy were similar in both groups (26% of patients on LPV/RTV versus 28% on ATV). In addition, subjects switched to ATV had a reduction in new onset gastrointestinal symptoms, improvements in lipid parameters, and a lower usage of lipid lowering agents compared to subjects remaining on the LPV/RTV arm [Gatell, 2006]. This post-hoc analysis demonstrated that subjects switching from a stable, virologically suppressive, LPV/RTV based regimen to a RTV-boosted or RTV-sparing ATV based regimen maintained similar virologic control with fewer lipid abnormalities and better gastrointestinal tolerability.

The ARIES study was a 144-week, phase IIIB, randomized, open-label, multi-center study in 419 randomized patients who had achieved virologic suppression during the initial 36-week induction phase. In the induction phase, patients received ATV/RTV + abacavir sulfate/lamivudine (ABC/3TC) over 36 weeks and those achieving virologic suppression were subsequently randomized (1:1) to a simplification regimen of ATV + ABC/3TC or remained on the induction regimen and then followed for 108 weeks. At week 84 (48 weeks from the start of randomization), 86% of patients randomized to the simplification arm vs. 81% of patients remaining on the induction arm maintained virologic suppression to HIV-1 RNA <50 copies/mL (c/mL) [Squires, 2010a]. Virologic failure occurred in 0.5% of patients in the simplification arm compared to 3% of patients in the induction arm through 84 weeks. Results were similar among patients with entry viral loads above and below 100,000 c/mL. Patients on the simplification regimen had less hyperbilirubinemia and a reduction in total cholesterol and triglycerides. At week 120 (84 weeks from start of randomization), virologic efficacy was sustained between groups resulting in 84% of ATV + ABC/3TC versus 83% of ATV/RTV + ABC/3TC subjects maintaining suppression to <50 c/mL [Squires ,2010b]. No differences in response rates by baseline viral load were observed between groups. The overall rate of virologic failure was 2% and comparable between groups. Subjects in the simplification arm continued to demonstrate favorable lipid benefits and maintained bilirubin reductions through 120 weeks.

ABC/3TC is a dual nucleoside combination that is also commonly used with RTV-boosted ATV, but may also be used with unboosted ATV in appropriate patient populations. Recent data have shown that the combination of unboosted ATV plus ABC/3TC effectively maintains virologic suppression (plasma HIV-1 RNA <50 c/mL) after initial induction with RTV-boosting of ATV in HIV-infected patients [Delfraissy, 2008; Squires, 2010b]. In addition, ABC/3TC has a well-established safety profile and relatively few drug interactions [EPZICOM® Package Insert, 2009].

The current study will evaluate whether subjects receiving a RTV-containing regimen of ATV/RTV + TDF/FTC who have achieved virologic suppression can safely simplify to a RTV-sparing regimen of ATV + ABC/3TC and maintain virologic suppression through 48 weeks.

This study will also evaluate changes in neurocognition (via a computerized test battery), and cardiovascular, renal and bone biomarkers as exploratory endpoints. As the life expectancy of HIV-1 infected patients continues to improve, long-term consequences of chronic HIV-infection and potentially specific antiretroviral therapies is of active research interest. Additionally, this study will increase our knowledge regarding the incidence of clinically suspected abacavir hypersensitivity reaction (ABC HSR) after excluding patients who carry the HLA-B*5701 allele.

Section 1.2 (Rationale) previously read:

Protease inhibitor (PI)-based therapies containing low-dose RTV have demonstrated favourable efficacy, safety, and barrier to resistance among antiretroviral naïve and treatment experienced patients. However, RTV is associated with long-term gastrointestinal and metabolic toxicities, added pill burden and cost, and requires refrigeration.

ATV is the only currently licensed PI that can be safely administered with or without RTV. Several trials [Delfraissy, 2008; Gatell, 2007; Squires, 2009] investigating an induction-simplification treatment strategy to achieve rapid initial viral load reduction with RTV-boosted ATV followed by simplification to unboosted ATV have demonstrated comparable efficacy. Although there are limited published data utilizing this strategy in clinical practice, a RTV-sparing strategy with ATV has shown improvement in lipids, bilirubin, low rate of virologic failure, and patient acceptance based on improved tolerability and fewer side effects [Santos, 2009; Pavie, 2009; Giuntini, 2010].

The need to find alternative treatment strategies that do not require the use of RTV is of continued interest among clinicians who perceive this as an unmet patient need. Data from this study will support this simplification strategy as a long term treatment option for subjects and clinicians who prefer a RTV-sparing regimen.

The benefit to patients would be a simplified and cost effective treatment regimen, with potentially fewer adverse effects and no need for refrigeration. The risks to this approach are potentially lower ATV trough levels that may lead to resistance development and the unknown long-term impact of switching TDF/FTC for ABC/3TC. However, this risk to patients may be small and mitigated due to the expected low rate of virologic failure observed following this strategy [Squires, 2009; Delfraissy, 2008], the ability to change to an alternative treatment regimen quickly at a relatively low level of viral replication, the expected emergence of mutation pattern(s) readily rescued by other current available PIs, and the shorter study duration.

Potential risks of using this simplification study design are that some subjects may experience viral rebound and potentially develop resistance mutations rendering the simplification regimen less effective [Malan, 2006; McGrath, 2006]. However, subjects

randomized to the simplification arm who experience viral rebound will have the opportunity to switch to an alternative regimen at the time of confirmed virologic failure. Overall the risk of virologic failure in the RTV-sparing arm is small and readily manageable compared to the benefit of protection from long-term complications of RTV-containing therapy such as hyperlipidemia, insulin resistance, lipoatrophy, and gastrointestinal intolerance and associated complexities for HIV-infected patients with other co-morbidities.

The long-term implications of this strategy are currently unknown, but any risk is likely to be small given the short-term exposure to resistant virus and that ATV-resistant virus has been documented *in vitro* to be susceptible to alternative PIs.

Additionally, there is a small risk for subjects that are simplified to the ABC/3TC regimen to experience a suspected ABC HSR reaction. However, all subjects enrolled in this study will be HLA-B*5701 negative and therefore the risk for ABC HSR will be substantially reduced as evidenced by data in other HLA-B*5701 negative subjects receiving ABC-containing regimens in the ARIES and PREDICT-1 studies [Squires, 2009; Mallal, 2008].

And now reads:

Protease inhibitor (PI)-based therapies containing low-dose RTV have demonstrated favourable efficacy, safety, and barrier to resistance among antiretroviral naïve and treatment experienced patients. However, RTV is associated with long-term gastrointestinal and metabolic toxicities, added pill burden and cost.

ATV is the only currently licensed PI that can be safely administered with or without RTV. Several trials [Delfraissy, 2008; Gatell, 2007; Squires, 2010a; Squires, 2010b] investigating an induction-simplification treatment strategy to achieve rapid initial viral load reduction with RTV-boosted ATV followed by simplification to unboosted ATV have demonstrated comparable efficacy. Although there are limited published data utilizing this strategy in clinical practice, a RTV-sparing strategy with ATV has shown improvement in lipids, bilirubin, low rate of virologic failure, and patient acceptance based on improved tolerability and fewer side effects [Santos, 2009; Pavie, 2009; Giuntini, 2010].

The need to find alternative treatment strategies that do not require the use of RTV is of continued interest among clinicians who perceive this as an unmet patient need. Data from this study will support this simplification strategy as a long term treatment option for subjects and clinicians who prefer a RTV-sparing regimen.

The benefit to patients would be a simplified and cost effective treatment regimen, with potentially fewer adverse effects and potential reduction in drug-drug interactions. The risks to this approach are potentially lower ATV trough levels that may lead to resistance development and the unknown long-term impact of switching TDF/FTC for ABC/3TC. However, this risk to patients may be small and mitigated due to the expected low rate of virologic failure observed following this strategy [Squires, 2010a; Squires, 2010b; Delfraissy, 2008], the ability to change to an alternative treatment regimen quickly at a

relatively low level of viral replication, the expected emergence of mutation pattern(s) readily rescued by other current available PIs, and the shorter study duration.

Potential risks of using this simplification study design are that some subjects may experience viral rebound and potentially develop resistance mutations rendering the simplification regimen less effective [Malan, 2006; McGrath, 2006]. However, subjects randomized to the simplification arm who experience viral rebound will have the opportunity to switch to an alternative regimen at the time of confirmed virologic failure. Overall the risk of virologic failure in the RTV-sparing arm is small and readily manageable compared to the benefit of protection from long-term complications of RTV-containing therapy such as hyperlipidemia, insulin resistance, lipoatrophy, and gastrointestinal intolerance and associated complexities for HIV-infected patients with other co-morbidities.

The long-term implications of this strategy are currently unknown, but any risk is likely to be small given the short-term exposure to resistant virus and that ATV-resistant virus has been documented *in vitro* to be susceptible to alternative PIs.

Additionally, there is a small risk for subjects that are simplified to the ABC/3TC regimen to experience a suspected ABC HSR reaction. However, all subjects enrolled in this study will be HLA-B*5701 negative and therefore the risk for ABC HSR will be substantially reduced as evidenced by data in other HLA-B*5701 negative subjects receiving ABC-containing regimens in the ARIES and PREDICT-1 studies [Squires, 2010a; Mallal, 2008].

<u>Section 3.1 (Study Design) previously read:</u>

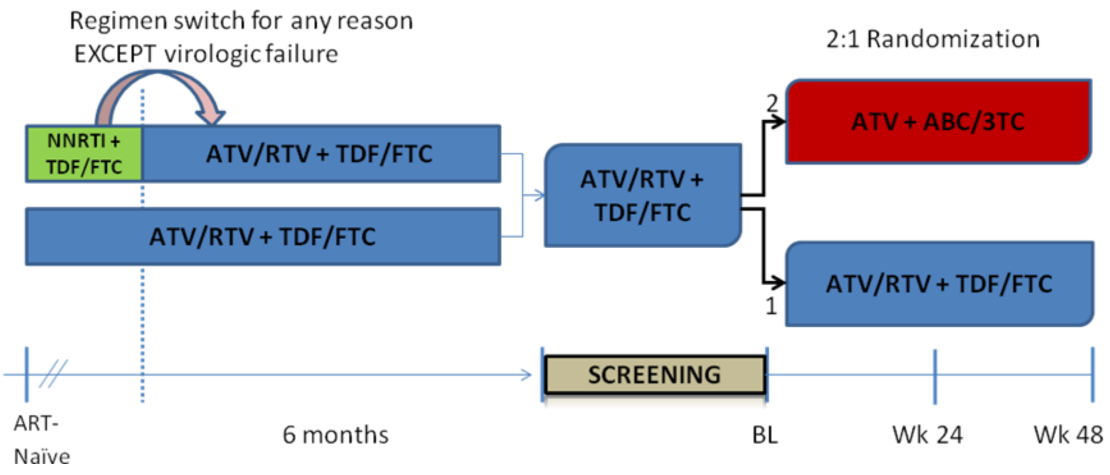

This is a phase IV, prospective, randomized, open-label, multicenter, non-inferiority study of the safety, efficacy, and tolerability of ATV + ABC/3TC once daily compared to ATV/RTV + TDF/FTC once daily for 48 weeks in HIV-1 infected, HLA-B*5701-negative subjects who are currently receiving a stable regimen of ATV/RTV + TDF/FTC once daily and are virologically suppressed (plasma HIV-1 RNA <50 c/mL).

ATV/RTV + TDF/FTC once daily should be the subjects initial or first and only switch regimen. First switch is defined as changing from an initial regimen of any licensed NNRTI + TDF/FTC to ATV/RTV + TDF/FTC. The switch must not be due to virologic failure.

A minimum of 300 subjects meeting eligibility criteria will be stratified by initial antiretroviral regimen received (NNRTI + TDF/FTC or ATV/RTV + TDF/FTC) and randomized 2:1 to receive one of the following anti-retroviral therapy (ART)-regimens below for 48 weeks. Subjects will have twice the chance to be randomized to the simplification arm than the continuation arm.

**Treatment Arm A: (Simplification)**	ATV 400 mg once daily + ABC/3TC 600 mg/300 mg once daily
**Treatment Arm B: (Continuation)**	ATV/RTV 300 mg/100 mg once daily + TDF/FTC 300 mg/200 mg once daily

Subjects who enter the Screening period of this study must continue receiving their ATV/RTV + TDF/FTC regimen up to, but not including, the Baseline visit (Day 1). Subjects will begin randomized treatment on Day 1.

This study consists of up to a 28-35 day Screening period, a 48 week Treatment period (Day 1 through Week 48) and a follow-up period (contact approximately 2-4 weeks after the Week 48 visit or Withdrawal visit).

Study participation is considered complete when a subject has completed study procedures through Week 48.

Supplementary study conduct information not mandated to be present in this protocol is provided in the accompanying Study Procedures Manual (SPM). The SPM will provide the site personnel with administrative and detailed technical information that does not impact subject safety.

And now reads:

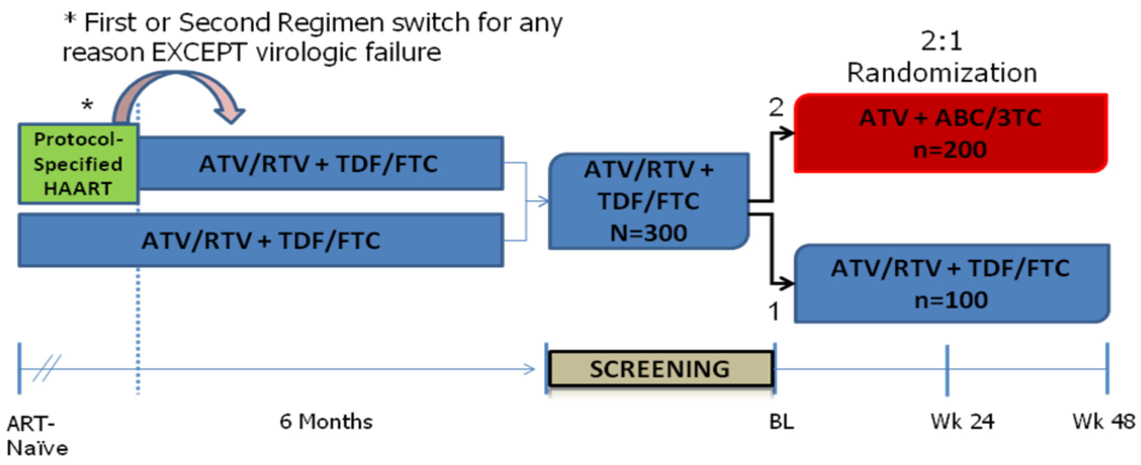

This is a phase IV, prospective, randomized, open-label, multicenter, non-inferiority study of the safety, efficacy, and tolerability of ATV + ABC/3TC once daily compared to ATV/RTV + TDF/FTC once daily for 48 weeks in HIV-1 infected, HLA-B*5701-negative subjects who are currently receiving a stable regimen of ATV/RTV + TDF/FTC once daily and are virologically suppressed (plasma HIV-1 RNA ≤75 c/mL).

ATV/RTV + TDF/FTC once daily must be the subject's initial, or first or second switch regimen. However, regimen switches must not have been due to virologic failure.

A minimum of 300 subjects meeting eligibility criteria will be stratified by initial antiretroviral regimen received (ATV/RTV + TDF/FTC as initial regimen OR as the first or second switch regimen) and randomized 2:1 to receive one of the following anti-retroviral therapy (ART)-regimens below for 48 weeks. Subjects will have twice the chance to be randomized to the simplification arm than the continuation arm.

Treatment Arm A: (Simplification) ATV 400 mg once daily + ABC/3TC 600 mg/300 mg once daily

Treatment Arm B: (Continuation) ATV/RTV 300 mg/100 mg once daily + TDF/FTC 300 mg/200 mg once daily

Subjects who enter the Screening period of this study must continue receiving their ATV/RTV + TDF/FTC regimen up to, but not including, the Baseline visit (Day 1). Subjects will begin randomized treatment on Day 1.

This study consists of a 35 day Screening period, a 48 week Treatment period (Day 1 through Week 48) and a follow-up period (contact approximately 2-4 weeks after the Week 48 visit or Withdrawal visit).

Study participation is considered complete when a subject has completed study procedures through Week 48.

Supplementary study conduct information not mandated to be present in this protocol is provided in the accompanying Study Procedures Manual (SPM). The SPM will provide the site personnel with administrative and detailed technical information that does not impact subject safety.

<u>Section 4.2 (Inclusion Criteria) previously read:</u>

Subjects eligible for enrolment in the study must meet all of the following criteria:

1. Adults ≥ 18 years of age.
2. Receiving a once-daily regimen of ATV/RTV (300 mg/100 mg) + TDF/FTC (300 mg/200 mg) as his/her INITIAL or FIRST AND ONLY SWITCH regimen for at least 6 months prior to or by the first day of Screening.
    - First switch is defined as changing from an initial regimen of any licensed NNRTI + TDF/FTC to ATV/RTV + TDF/FTC.
3. Virologically suppressed on ATV/RTV + TDF/FTC
    - Virologically suppressed is defined as HIV-1 RNA <50 c/mL at 2 consecutive timepoints, one of which is at Screening and the other at least 28 days prior to Screening
4. A female is eligible to enter and participate in the study if she is of:
    a. Non-childbearing potential (ie, physiologically incapable of becoming pregnant, including any female who is pre-menarchal or post-menopausal); or,
    b. Child-bearing potential, has a negative pregnancy test at Screening (serum β-Human chorionic gonadotropins (HCG) and Baseline (urine β-HCG) and agrees to one of the following methods of contraception (any contraception method must be used consistently and correctly, i.e., in accordance with both the approved product label and the instructions of a physician):
        - Complete abstinence from sexual intercourse from 2 weeks prior to administration of the Investigational Products, throughout the study, and for at least 2 weeks after discontinuation of all study medications
        - Double barrier method (male condom/spermicide, male condom/diaphragm, diaphragm/spermicide). Hormonal contraception will not be considered adequate for inclusion into this study.
        - Any intrauterine device (IUD) with published data showing that the expected failure rate is <1% per year.
        - Sterilization (female subject or male partner of female subject).

All subjects participating in the study should be counselled on the practice of safer sexual practices including the use of effective barrier methods (e.g. male condom/spermicide).

<u>And now reads:</u>

1. Adults $\geq$ 18 years of age.
2. Receiving a once-daily regimen of ATV/RTV (300 mg/100 mg) + TDF/FTC (300 mg/200 mg) for at least 6 months prior to the first day of Screening.
    - ATV/RTV+TDF/FTC must be the subject's initial regimen or first or second switch regimen.
        - Initial regimen is defined as the first regimen received by a previously antiretroviral naïve subject
        - Any change of antiretroviral therapy, whether of a single drug or multiple drugs simultaneously, is considered a regimen switch.
        - Subjects must not have switched due to virologic failure.
        - If ATV/RTV + TDF/FTC is a subject's first or second switch regimen, then the subject may <u>only</u> have received the following prior regimens:
            - Any currently licensed NNRTI in combination with either TDF/FTC or ZDV/3TC
            - RTV-boosted PI in combination with TDF/FTC or ZDV/3TC
            - An alternative prior regimen not listed above ONLY after consultation and assent from the Sponsor on a case-by-case basis
3. Virologically suppressed on ATV/RTV + TDF/FTC
    - Virologically suppressed is defined as HIV-1 RNA $\leq$75 c/mL at 2 consecutive timepoints, one of which is at Screening and the other at least 28 days prior to Screening
4. A female is eligible to enter and participate in the study if she is of:
    a. Non-childbearing potential (ie, physiologically incapable of becoming pregnant, including any female who is pre-menarchal or post-menopausal); or,
    b. Child-bearing potential, has a negative pregnancy test at Screening (serum β-Human chorionic gonadotropins (HCG) and Baseline (urine β-HCG) and agrees to one of the following methods of contraception (any contraception method must be used consistently and correctly, i.e., in accordance with both the approved product label and the instructions of a physician):
        - Complete abstinence from sexual intercourse from 2 weeks prior to administration of the Investigational Products, throughout the study, and for at least 2 weeks after discontinuation of all study medications
        - Double barrier method (male condom/spermicide, male condom/diaphragm, diaphragm/spermicide). Hormonal contraception will not be considered adequate for inclusion into this study.

- Any intrauterine device (IUD) with published data showing that the expected failure rate is <1% per year.
- Sterilization (female subject or male partner of female subject).

All subjects participating in the study should be counselled on the practice of safer sexual practices including the use of effective barrier methods (e.g. male condom/spermicide).

Section 4.2 (Exclusion Criteria) previously read:

Subjects meeting any of the following criteria must not be enrolled in the study:

1. Evidence of virologic failure at any time defined as two consecutive plasma HIV-1 RNA levels ≥200 c/mL after initial suppression to HIV-1 RNA <50 c/mL.
2. Any known HIV genotyping results indicating that the subject has virus containing any of the following HIV-1 mutations:
   - Reverse transcriptase mutations: K65R, K70E, L74V, M184I/V, or Y115F
   - Combination of two or more thymidine analog mutations: M41L, D67N, K70R, K219Q or E that include changes at either L210 or T215
   - Three or more of the following HIV-1 protease mutations associated with atazanavir resistance: D30, V32, M36, M46, I47, G48, I50, I54, A71, G73, V77, V82, I84, N88, and L90

   *NOTE: A Baseline genotype is NOT required to determine eligibility.
3. HLA-B*5701 positive.
4. Hypersensitivity to any component of the study drugs.
5. Pregnant or breastfeeding females.
6. Enrolled in one or more investigational drug protocols within 30 days of Screening.
7. An active Center for Disease Control and Prevention (CDC) Category C disease, except cutaneous Kaposi's sarcoma not requiring systemic therapy during the trial (see Appendix 1).
8. Ongoing clinically relevant hepatitis at Screening and/or positive for Hepatitis B (+ HbsAg).
9. Creatinine clearance <50 mL/min via the Cockroft-Gault method (Cockroft, 1976). A single repeat is allowed to determine eligibility.
10. Verified Grade 4 laboratory abnormality at Screening unless the Investigator can provide a compelling explanation (e.g. elevated Creatine Phosphokinase (CPK) due to exercise) for the laboratory result(s) and has the assent of the Sponsor. A single repeat is allowed to determine eligibility.
11. Any other laboratory abnormality or medical condition at Screening, which, in the opinion of the investigator, would preclude the subject's participation in the study (e.g. elevated liver function tests (LFTs) or pancreatitis, etc).
12. Immunization within 30 days prior to first dose of Investigational Product.

13. Any exposure to treatment with immunomodulating agents (such as systemic corticosteroids, interleukins, or interferons) or receipt of an HIV-1 immunotherapeutic vaccine within 90 days prior to Screening. Subjects using inhaled corticosteroids or short-course systemic corticosteroids (≤14 days) are eligible for enrollment.
14. Treatment with radiation therapy or cytotoxic chemotherapeutic agents within 90 days prior to Screening, or an anticipated need for these agents within the study period.
15. Treatment within 30 days prior to first dose of Investigational Product for or an anticipated need during the study of any medications which can have interactions with the study medications, TDF, FTC, ABC, 3TC, ATV and/or RTV, as described in current product labelling (e.g. use of proton pump inhibitors with ATV).

Specific information regarding warnings, precautions, contraindications, adverse events, and other pertinent information on the Investigational Product that may impact subject eligibility is provided in each specific product label.

And now reads:

Subjects meeting any of the following criteria must not be enrolled in the study:

1. Evidence of virologic failure at any time defined as two consecutive plasma HIV-1 RNA levels ≥200 c/mL after initial suppression to HIV-1 RNA ≤75 c/mL.
2. Any known HIV genotyping results indicating that the subject has virus containing any of the following HIV-1 mutations:
    - Reverse transcriptase mutations: K65R, K70E, L74V, M184I/V, or Y115F
    - Combination of two or more thymidine analog mutations: M41L, D67N, K70R, K219Q or E that include changes at either L210 or T215
    - Three or more of the following HIV-1 protease mutations associated with atazanavir resistance: D30, V32, M36, M46, I47, G48, I50, I54, A71, G73, V77, V82, I84, N88, and L90

    *NOTE: A Baseline genotype is NOT required to determine eligibility.
3. HLA-B*5701 positive.
4. Hypersensitivity to any component of the study drugs.
5. Pregnant or breastfeeding females.
6. Enrolled in one or more investigational drug protocols within 30 days of Screening.
7. An active Center for Disease Control and Prevention (CDC) Category C disease, except cutaneous Kaposi's sarcoma not requiring systemic therapy during the trial (see Appendix 1).
8. Ongoing clinically relevant hepatitis at Screening and/or positive for Hepatitis B (+ HbsAg).

9. Creatinine clearance <50 mL/min via the Cockroft-Gault method [Cockroft, 1976]. A single repeat is allowed to determine eligibility.
10. Verified Grade 4 laboratory abnormality at Screening unless the Investigator can provide a compelling explanation (e.g. elevated Creatine Phosphokinase (CPK) due to exercise) for the laboratory result(s) and has the assent of the Sponsor. A single repeat is allowed to determine eligibility.
11. Any other laboratory abnormality or medical condition at Screening, which, in the opinion of the investigator, would preclude the subject's participation in the study (e.g. elevated liver function tests (LFTs) or pancreatitis, etc).
12. Immunization within 30 days prior to first dose of Investigational Product.
13. Any exposure to treatment with immunomodulating agents (such as systemic corticosteroids, interleukins, or interferons) or receipt of an HIV-1 immunotherapeutic vaccine within 90 days prior to Screening. Subjects using inhaled corticosteroids or short-course systemic corticosteroids (≤14 days) are eligible for enrollment.
14. Treatment with radiation therapy or cytotoxic chemotherapeutic agents within 90 days prior to Screening, or an anticipated need for these agents within the study period.
15. Treatment within 30 days prior to first dose of Investigational Product for or an anticipated need during the study of any medications which can have interactions with the study medications, TDF, FTC, ABC, 3TC, ATV and/or RTV, as described in current product labelling (e.g. use of proton pump inhibitors with ATV).
16. Any previous abacavir-containing regimen

Specific information regarding warnings, precautions, contraindications, adverse events, and other pertinent information on the Investigational Product that may impact subject eligibility is provided in each specific product label.

Section 4.4.1 (Subject Withdrawal from Study) previously read:

A subject must be withdrawn from the study when:

- A subject becomes Hepatitis B positive (+ HbsAg).
- Subject requires the use of any prohibited study medication (see Prohibited Medications, Section 5.7.2).
- A subject is significantly non-compliant with the requirements of the protocol (based upon the discretion of the investigator).
- A subject becomes pregnant (see Pregnancy, Section 6.4.8).
- A subject has an adverse experience that would, in the investigator's judgment, make continued participation in the study an unacceptable risk.
- Subject experiences a toxicity that meets the criteria for withdrawal (see Toxicity Management, Section 6.4.4)

- Subjects who become prisoners or become involuntarily incarcerated for treatment of either a psychiatric or physical (e.g., infectious disease) illness.
- Subject has a plasma HIV-1 RNA ≥2000 c/mL at the confirmatory virologic failure timepoint.
- Subject has two consecutive HIV-1 RNA measurements ≥2000 c/mL at any time.
- The Sponsor discontinues the study.

A subject may voluntarily discontinue participation in this study at any time. The investigator may also, at his or her discretion, withdraw the subject from participating in this study at any time.

Management of Withdrawn Subjects

If a subject is withdrawn from the study for any reason, the investigator must make every effort to perform the evaluations noted in the Time and Events table (see Section 6.1).

All data from the withdrawal visit should be recorded, as they comprise an essential evaluation that should be done prior to discharging any subject from the study.

If a subject is withdrawn from the study due to an AE (See Section 6.4.5.1) or serious adverse event (SAE) (See Section 6.4.5.2), the procedures stated in Section 6.4.5 (AEs and SAEs) must be followed and the AE must be followed-up until resolution.

Withdrawn subjects will not be replaced.

<u>And now reads:</u>

A subject must be withdrawn from the study when:

- A subject becomes Hepatitis B positive (+ HbsAg).
- Subject requires the use of any prohibited study medication, unless explicit approval is given by the Sponsor in consultation with the Investigator (see Prohibited Medications, Section 5.7.2).
- A subject is significantly non-compliant with the requirements of the protocol (based upon the discretion of the investigator).
- A subject becomes pregnant (see Pregnancy, Section 6.4.8).
- A subject has an adverse experience that would, in the investigator’s judgment, make continued participation in the study an unacceptable risk.
- Subject experiences a toxicity that meets the criteria for withdrawal (see Toxicity Management, Section 6.4.4)
- Subjects who become prisoners or become involuntarily incarcerated for treatment of either a psychiatric or physical (e.g., infectious disease) illness.
- Subject has a plasma HIV-1 RNA ≥2000 c/mL at the confirmatory virologic failure timepoint.

- Subject has two consecutive HIV-1 RNA measurements ≥2000 c/mL at any time.
- The Sponsor discontinues the study.

A subject may voluntarily discontinue participation in this study at any time. The investigator may also, at his or her discretion, withdraw the subject from participating in this study at any time.

Management of Withdrawn Subjects

If a subject is withdrawn from the study for any reason, the investigator must make every effort to perform the evaluations noted in the Time and Events table (see Section 6.1).

All data from the withdrawal visit should be recorded, as they comprise an essential evaluation that should be done prior to discharging any subject from the study.

If a subject is withdrawn from the study due to an AE (See Section 6.4.5.1) or serious adverse event (SAE) (See Section 6.4.5.2), the procedures stated in Section 6.4.5 (AEs and SAEs) must be followed and the AE must be followed-up until resolution.

Withdrawn subjects will not be replaced.

Section 5.3 (Treatment Assignment) previously read:

Subjects will be assigned to study treatment in accordance with the randomization schedule. Subjects will be stratified by initial antiretroviral regimen received (NNRTI + TDF/FTC or ATV/RTV + TDF/FTC) and randomized 2:1 to receive one of the following anti-retroviral therapy (ART)-regimens below for 48 weeks. Subjects will have twice the chance to be randomized to the simplification arm than the continuation arm.

**Treatment Arm A: (Simplification)**	ATV 400 mg once daily + ABC/3TC 600 mg/300 mg once daily
**Treatment Arm B: (Continuation)**	ATV/RTV 300 mg/100 mg once daily + TDF/FTC 300 mg/200 mg once daily

If a subject is eligible for randomization, the investigator (or designee) will call RAMOS (Registration and Medication Ordering System) and the subject will be assigned a randomization number. The randomization code is on file with the Sponsor and with RAMOS.

Training on the use of RAMOS and detailed user worksheets will be provided by the Sponsor prior to study start.

Randomization numbers are unique and may not be reassigned to another study subject.

And now reads:

Subjects will be assigned to study treatment in accordance with the randomization schedule. Subjects will be stratified by initial antiretroviral regimen received (ATV/RTV + TDF/FTC as initial regimen OR as first or second switch regimen) and randomized 2:1

to receive one of the following anti-retroviral therapy (ART)-regimens below for 48 weeks. Subjects will have twice the chance to be randomized to the simplification arm than the continuation arm.

**Treatment Arm A: (Simplification)**	ATV 400 mg once daily + ABC/3TC 600 mg/300 mg once daily
**Treatment Arm B: (Continuation)**	ATV/RTV 300 mg/100 mg once daily + TDF/FTC 300 mg/200 mg once daily

If a subject is eligible for randomization, the investigator (or designee) will call RAMOS (Registration and Medication Ordering System) and the subject will be assigned a randomization number. The randomization code is on file with the Sponsor and with RAMOS.

Training on the use of RAMOS and detailed user worksheets will be provided by the Sponsor prior to study start.

Randomization numbers are unique and may not be reassigned to another study subject.

Section 6 (Study Assessments and Procedures) Previously read:

Written informed consent must be obtained from each potentially eligible subject (or his/her legal representative) by study site personnel **prior** to the initiation of any Screening procedures as outlined in this protocol. The consent form must have been approved by the Institutional Review Board / Independent Ethics Committee (IRB/IEC). After signing an informed consent, subjects will complete Screening assessments to determine subject eligibility.

Each subject being screened for study enrollment evaluation will be assigned a subject number. This number will be given sequentially in chronological order of subject presentation according to a numeric roster provided by the Sponsor.

Subjects who qualify must return within 28-35 days of the Screening visit to begin study treatment. Subjects not meeting all inclusion and exclusion criteria at initial screen may be re-screened at the discretion of the investigator after consultation with the Sponsor**.** A subject, who is randomized into the trial and subsequently withdraws from the study for any reason, may not be re-screened.

A single repeat test per analyte is allowed during the Screening period. **However, a repeat plasma HIV-1 RNA measurement is not allowed if the initial Screening value is >50 c/mL.**

And now reads:

Written informed consent must be obtained from each potentially eligible subject (or his/her legal representative) by study site personnel **prior** to the initiation of any Screening procedures as outlined in this protocol. The consent form must have been approved by the Institutional Review Board / Independent Ethics Committee (IRB/IEC). After signing an informed consent, subjects will complete Screening assessments to determine subject eligibility.

Each subject being screened for study enrollment evaluation will be assigned a subject number. This number will be given sequentially in chronological order of subject presentation according to a numeric roster provided by the Sponsor.

Subjects who qualify must return no more than 35 days from the day of the Screening visit to begin study treatment. Subjects not meeting all inclusion and exclusion criteria at initial screen may be re-screened once at the discretion of the investigator after consultation with the Sponsor**.** A subject, who is randomized into the trial and subsequently withdraws from the study for any reason, may not be re-screened.

A single repeat test per analyte is allowed during the Screening period. **However, a repeat plasma HIV-1 RNA measurement is not allowed if the initial Screening value is >75 c/mL.**

Section 6.1 (Time and Events Schedule) previously read:

Table 1 Time and Events Table

Procedures/Clinical Evaluation	Screen	Baseline	Treatment Week						Early W/D[a]	F/U[b]	Confirmation of VF[e,i]
			2	4	12	24	36	48			
**Study Assessments**											
Written Informed Consent (including PGx)	x										
Inclusion/Exclusion Criteria	x	x									
Subject Demography	x	x									
Medical History	x	x									
HIV-1 Associated Conditions		x	x	x	x	x	x	x	x		
CDC Classification	x	x	x	x	x	x	x	x	x		
HIV risk factors/mode of transmission		x									
Limited Physical Exam (Body Weight, Height)		x									
Dispense Investigational Product		x		x	x	x	x				
**Safety Assessments**											
Vital Signs (BP, HR)		x				x		x	x		
Concomitant Medication	x	x	x	x	x	x	x	x	x		x
Smoking History[c]		x[c]				x[c]		x[c]	x[c]		
Adverse Events	x[d]	x[d]	x	x	x	x	x	x	x	x	x
**Laboratory Assessments**											
Quantitative Plasma HIV-1 RNA	x[e]	x[e]	x[e]	x[e]	x[e]	x[e]	x[e]	x[e]	x[e]	x (if needed)	x
CD4+/CD8+ Lymphocyte Subsets	x	x		x	x	x	x	x	x	x (if needed)	x
Hematology	x	x	x	x	x	x	x	x	x	x (if needed)	
Chemistry[f]	x	x	x	x	x	x	x	x	x	x (if needed)	

**Procedures/Clinical Evaluation**	**Screen**	**Baseline**	**Treatment Week**						**Early W/D**[a]	**F/U**[b]	**Confirmation of VF**[e,i]
			**2**	**4**	**12**	**24**	**36**	**48**			
Fasting lipids and Fasting Glucose[f]		x		x		x		x	x[f]		
CrCl (via C-G) and GFR (via MDRD)	x	x	x	x	x	x	x	x	x	x	
HLA-B*5701 determination	x										
Hepatitis B antibody (HBsAg)	x										
Hep C (Anti- HCV Ab)		x									
Pregnancy Test[g]	x	x									
Urine Chemistry Analytes		x				x		x	x		
PGx Sampling[h]		x									
Plasma for storage (genotypic and phenotypic analyses)		x	x	x	x	x	x	x	x		x
**Exploratory Assessments**											
Blood CV Biomarkers		x				x		x	x		
Blood Bone Biomarkers		x				x		x	x		
Urine Biomarkers		x				x		x	x		
Serum Parathyroid hormone		x				x		x	x		
Neurocognitive Battery		x				x		x	x		

a. W/D – Study withdrawal. Withdrawal evaluations performed if subject discontinues prematurely from the study.
b. F/U – Study follow-up. Subjects will be contacted approximately 2-4 weeks after Week 48 or W/D by telephone for follow-up visit. If resolution of on-going AE(s) or confirmation of virologic failure is required the subject will return to the clinic for follow-up visit.
c. Smoking history will be used to calculate Framingham cardiovascular risk score which includes assessment of age, gender, total cholesterol, HDL, SBP, BP lowering medication use and smoking use.
d. Only SAEs related to study participation will be collected between obtaining written informed consent and administration of Investigational Products on Day 1.
e. A confirmatory HIV-1 RNA should be scheduled at least 28 days after a plasma HIV-1 RNA $\geq$400 c/mL result for confirmation of virologic failure.
f. Fasting is required (no food in previous 6-8 hours) except for the early W/D visit.
g. Required for females of childbearing potential only. Serum pregnancy test to be performed at Screening; Urine pregnancy test to be performed at Baseline. Pregnancy test may be performed at the discretion of the investigator if pregnancy is suspected at any time during the trial.
h. PGx sample collection may be performed at any time starting at Baseline although it should be performed at the earliest time point possible.
i. Confirmation of virologic failure. Subjects should be asked about potential compliance issues, illness or recent immunizations. It is recommended that subjects be adherent to study medications for at least 28 days before returning for a confirmatory HIV-1 RNA.

<u>And now reads:</u>

6.1 Time and Events Schedule

Table 1 Time and Events Table

Procedures/Clinical Evaluation	Screen	Baseline	Treatment Week						Early W/D[a]	F/U[b]	Confirmation of VF[e,i]
			2	4	12	24	36	48			
**Study Assessments**											
Written Informed Consent (including PGx)	x										
Inclusion/Exclusion Criteria	x	x									
Subject Demography	x	x									
Medical History	x	x									
HIV-1 Associated Conditions		x	x	x	x	x	x	x	x		
CDC Classification	x	x	x	x	x	x	x	x	x		
HIV risk factors/mode of transmission		x									
Historical nadir CD4 value[k]		X[k]									
Limited Physical Exam (Body Weight, Height)		x									
Dispense Investigational Product		x		x	x	x	x				
**Safety Assessments**											
Vital Signs (BP, HR)		x				x		x	x		
Concomitant Medication	x	x	x	x	x	x	x	x	x		x
Smoking History[c]		x[c]				x[c]		x[c]	x[c]		
Adverse Events	x[d]	x[d]	x	x	x	x	x	x	x	x	x
**Laboratory Assessments**											
Quantitative Plasma HIV-1 RNA	x[e]	x[e]	x[e]	x[e]	x[e]	x[e]	x[e]	x[e]	x[e]	x (if needed)	x
CD4+/CD8+ Lymphocyte Subsets	x	x		x	x	x	x	x	x	x (if needed)	x
Hematology	x	x	x	x	x	x	x	x	x	x (if needed)	
Chemistry	x	x	x	x	x	x	x	x	x	x (if needed)	
Fasting lipids and Fasting Glucose[f]		x		x		x		x	x		

**Procedures/Clinical Evaluation**	**Screen**	**Baseline**	**Treatment Week**						**Early W/D**[a]	**F/U**[b]	**Confirmation of VF**[e,i]
			**2**	**4**	**12**	**24**	**36**	**48**			
CrCl (via C-G) and GFR (via MDRD)	x	x	x	x	x	x	x	x	x	X (if needed)	
HLA-B*5701 determination	x										
Hepatitis B antibody (HBsAg)	x										
Hep C (Anti- HCV Ab)		x									
Pregnancy Test[g]	x	x						X	X		
Urine Chemistry Analytes		x				x		x	x		
PGx Sampling[h]		x									
Plasma for storage (genotypic and phenotypic analyses)		x	x	x	x	x	x	x	x		x
**Exploratory Assessments**											
Blood CV Biomarkers		x				x		x	x		
Blood Bone Biomarkers		x				x		x	x		
Urine Biomarkers		x				x		x	x		
Serum Parathyroid hormone		x				x		x	x		
CogState Neurocognitive Battery[j]		x				x		x	x		

a. W/D – Study withdrawal. Withdrawal evaluations performed if subject discontinues prematurely from the study.
b. F/U – Study follow-up. Subjects will be contacted approximately 2-4 weeks after Week 48 or W/D by telephone for follow-up visit. If resolution of on-going AE(s) or confirmation of virologic failure is required the subject will return to the clinic for follow-up visit.
c. Smoking history will be used to calculate Framingham cardiovascular risk score which includes assessment of age, gender, total cholesterol, HDL, SBP, BP lowering medication use and smoking use.
d. Only SAEs related to study participation will be collected between obtaining written informed consent and administration of Investigational Products on Day 1.
e. A confirmatory HIV-1 RNA should be scheduled at least 28 days after a plasma HIV-1 RNA $\geq$400 c/mL result for confirmation of virologic failure.
f. Fasting is required (no food in previous 6-8 hours) except for the early W/D visit.
g. Required for females of childbearing potential only. Serum pregnancy test to be performed at Screening, Week 48 and Early Withdrawl; Urine pregnancy test to be performed at Baseline. Pregnancy test may be performed at the discretion of the investigator if pregnancy is suspected at any time during the trial.
h. PGx sample collection may be performed at any time starting at Baseline although it should be performed at the earliest time point possible.
i. Confirmation of virologic failure. Subjects should be asked about potential compliance issues, illness or recent immunizations. It is recommended that subjects be adherent to study medications for at least 28 days before returning for a confirmatory HIV-1 RNA.
j. A pre-baseline neurocognitive test is required to ensure that the subject is familiar with the testing modality. This test will be performed on the same day as the baseline test.
k. This value can be recorded at any time during the study using the subject's medical history

Section 6.4.2 (Exploratory Evaluations) previously read:

Exploratory assessments will consist of the following investigations:

- Neurocognitive assessment via computerized questionnaire will be assessed at Baseline and Weeks 24, 48 and W/D.
- Blood or urine samples for biomarker assessments will be collected at Baseline and Weeks 24, 48 and W/D.

And now reads:

Exploratory assessments will consist of the following investigations:

- The pre-Baseline nadir CD4+ cell count will be collected. This may be collected at any time during the study.
- Neurocognitive assessment via computerized questionnaire will be assessed at Baseline and Weeks 24, 48 and W/D.
    - A pre-baseline neurocognitive assessment is required to ensure that the subject is familiar with the testing modality. The pre-baseline assessment will be performed on the same day as the baseline assessment. The pre-baseline assessment data will not be collected.
- Blood or urine samples for biomarker assessments will be collected at Baseline and Weeks 24, 48 and W/D.

Section 6.4.4.6 (Renal toxicity) subheading *Decline in renal function* previously read:

The Cockroft-Gault equation for estimating creatinine clearance (CrCl) will be used to determine changes in dosing for both TDF/FTC and ABC/3TC in the event of declining renal function.

For subjects who experience progression to an estimated CrCl (calculated by Cockroft-Gault equation) to <50 mL/min judged by the investigator to be attributed to study medication, the offending agent(s) must be discontinued. No dose-reduction of the offending agent(s) will be allowed.

For subjects who experience progression to an estimated GFR to <50 mL/min judged by the investigator to be NOT study drug-related, the investigator will choose from one of the following two management options for study medications:

1. Discontinue study medication(s) and switch to another agent in the same class and remain on study.
2. Dose reduce study medication (s) as indicated in prescribing information and remain on study.

*Note: The Sponsor will provide ATV, RTV, ABC/3TC, TDF/FTC, 3TC/ZDV or FPV for the purpose of constructing an alternative treatment regimen for decline in renal

function. Reimbursement will be offered for the cost associated with use of any agent not provided by the Sponsor.

And now reads:

The Cockroft-Gault equation for estimating creatinine clearance (CrCl) will be used to determine changes in dosing for both TDF/FTC and ABC/3TC in the event of declining renal function.

For subjects who experience progression to an estimated CrCl (calculated by Cockroft-Gault equation) to <50 mL/min judged by the investigator to be attributed to study medication, the offending agent(s) must be discontinued. No dose-reduction of the offending agent(s) will be allowed.

For subjects who experience progression to an estimated CrCl (calculated by Cockroft-Gault equation) to <50 mL/min to be NOT study drug-related, the investigator will choose from one of the following two management options for study medications:

1. Discontinue study medication(s) and switch to another agent in the same class and remain on study.
2. Dose reduce study medication (s) as indicated in prescribing information and remain on study.

*Note: The Sponsor will provide ATV, RTV, ABC/3TC, TDF/FTC, 3TC/ZDV or FPV for the purpose of constructing an alternative treatment regimen for decline in renal function. Reimbursement will be offered for the cost associated with use of any agent not provided by the Sponsor.

Section 8.3.5.1 (Efficacy Anayses) subheading *Primary Analysis* previously read:

The primary efficacy objective of the study is to establish the non-inferiority of the EPZICOM arm to the Truvada arm in proportion of subjects with plasma HIV-1 RNA <50 c/mL at Week 24.

Statistical hypothesis testing on the difference in the proportion of subjects with HIV-1 RNA <50 c/mL at Week 24 between the two treatment arms being -0.12 or less versus the alternative that the difference is > -0.12 will be performed at a one-sided 0.025 significance level. This is equivalent to constructing a two-sided 95% confidence interval on the difference in proportions and comparing the lower bound of the 95% CI to -0.12. The null hypothesis will be rejected and hence the non-inferiority will be established if the lower limit of the confidence interval for the difference (EPZICOM arm minus Truvada) in proportion of subjects with HIV-1 RNA <50 c/mL at Week 24 is > - 0.12. However, if the lower limit of the 95% CI is ≤ -0.12, it cannot be ruled out that the EPZICOM arm is inferior to the Truvada arm. The Cochran-Mantel-Haenszel (CMH) test will be used in the analysis. Odds ratios, confidence intervals, and p-values will be computed: (1) stratifying by initial antiretroviral regimen received (NNRTI + TDF/FTC or ATV/RTV + TDF/FTC), (2) stratifying by center, and (3) without stratification.

And now reads:

The primary efficacy objective of the study is to establish the non-inferiority of the EPZICOM arm to the Truvada arm in proportion of subjects with plasma HIV-1 RNA <50 c/mL at Week 24.

Statistical hypothesis testing on the difference in the proportion of subjects with HIV-1 RNA <50 c/mL at Week 24 between the two treatment arms being -0.12 or less versus the alternative that the difference is > -0.12 will be performed at a one-sided 0.025 significance level. This is equivalent to constructing a two-sided 95% confidence interval on the difference in proportions and comparing the lower bound of the 95% CI to -0.12. The null hypothesis will be rejected and hence the non-inferiority will be established if the lower limit of the confidence interval for the difference (EPZICOM arm minus Truvada) in proportion of subjects with HIV-1 RNA <50 c/mL at Week 24 is > - 0.12. However, if the lower limit of the 95% CI is ≤ -0.12, it cannot be ruled out that the EPZICOM arm is inferior to the Truvada arm. The Cochran-Mantel-Haenszel (CMH) test will be used in the analysis. Odds ratios, confidence intervals, and p-values will be computed: (1) stratifying by initial antiretroviral regimen received (ATV/RTV + TDF/FTC as initial regimen or ATV/RTV + TDF/FTC as first or second switch regimen), (2) stratifying by center, and (3) without stratification.

The following two references were added to the reference section:

Squires KE, Young B, DeJesus E, et al. Similar efficacy and tolerability of atazanavir compared with atazanavir/ritonavir, each with abacavir/lamivudine after initial suppression with abacavir/lamivudine plus ritonavir-boosted atazanavir in HIV-1 infected patients. AIDS 2010;13:2019-27.

Squires K, DeJesus E, Bellos N, et al. Sustained virologic efficacy of atazanavir (ATV) versus atazanavir/ritonavir (ATV/ r), each in combination with abacavir/lamivudine (ABC/3TC)over 120 weeks: the ARIES trial. In: Program and abstracts of the 10th Annual Interscience Conference on Antimicrobial Agents and Chemotherapy (ICAAC); September 12-15, 2010; Boston, MA. Poster H-204.
